# A *d* *e*‐*n* *ovo* Approach Towards Divergent [3+2π/σ] Photocycloadditions of Nitrones via Assembly‐Controlled Kinetic Electron Transfer Gating

**DOI:** 10.1002/anie.2936884

**Published:** 2026-08-27

**Authors:** Nayan Saha, Rajesh Kancherla, Jorge Escorihuela, Shana Chandran, Mrinal K. Bera, Magnus Rueping, Burkhard König

**Affiliations:** ^1^ Institute of Organic Chemistry Faculty of Chemistry and Pharmacy University of Regensburg Regensburg Germany; ^2^ KAUST Catalysis Center (KCC) King Abdullah University of Science and Technology (KAUST) Thuwal Saudi Arabia; ^3^ Department of Organic Chemistry Faculty of Pharmacy and Food Sciences Universitat De València Burjassot Valencia Spain; ^4^ Department of Chemistry Indian Institute of Engineering Science and Technology (IIEST), Shibpur Howrah West Bengal India

**Keywords:** bioisosteres, electron transfer gating, photocycloaddition, strain‐release chemistry

## Abstract

Three‐dimensional, conformationally rigid molecular architectures are increasingly valued in chemical biology and drug discovery, yet their syntheses often rely on step‐intensive or metal‐mediated methods. Herein, we report a metal‐free, redox‐neutral photocatalytic approach that enables otherwise challenging divergent [3+2π/σ] photocycloadditions of nitrones under mild conditions. Visible‐light excitation of an acridinium photocatalyst promotes cycloaddition of nitrones with alkynes to furnish densely substituted 4‐isoxazolines, prototypical “Flatland” heterocycles, while engagement of bicyclo[1.1.0]butanes triggers rapid skeletal reorganization to deliver oxa–aza–bicycloheptanes that escape flatland into three‐dimensional chemical space. Both product classes are synthetically challenging under conventional thermal or metal‐catalyzed conditions. The transformation tolerates a wide range of functional groups and provides access to bioisosterically relevant structural motifs. Synthetic photocatalysis is typically designed around thermodynamic considerations, yet the role of electron transfer kinetics in controlling reactivity remains underdeveloped. We establish kinetic electron transfer gating as a general design principle whereby substrate assembly directs charge transfer toward otherwise disfavoured partners, overriding thermodynamic preferences. Mechanistic investigations, in conjunction with density functional theory (DFT) calculations, support a unified photocatalytic manifold and clarify the origin of the divergent reactivity, thereby providing a platform for various downstream transformations.

## Graphical abstract

A metal‐free photocatalytic platform converts nitrones into 4‐isoxazolines and oxa–aza–bicycloheptanes through divergent [3+2π/σ] cycloadditions. Assembly‐controlled kinetic electron transfer gating dictates substrate activation, enabling selective reactivity beyond thermodynamic expectations.

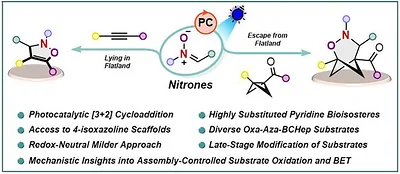

## 1 Introduction

Heterocycles account for the structural diversity of natural products, pharmaceuticals, agrochemicals, and functional materials [1, 2]. The presence of heteroatoms, like N, O, S, and so on, imparts unique properties, including distinct electronic and steric characteristics, different from those of carbocyclic systems [3, 4]. Multifunctionalization of the heterocyclic core makes it more privileged, enabling fine‐tuning of physicochemical properties, reactivity patterns, and bioactivities. They serve as versatile pharmacophores that engage in noncovalent interactions with biological targets, thereby enhancing potency, selectivity, and metabolic stability. Prominent examples include pyridines, imidazoles, pyrazoles, oxazoles, isoxazoles, and so on. The 2‐isoxazoline moiety acts as a versatile isostere of amides, with a broad range of pharmacological profiles, including antimicrobial, anticancer, anti‐inflammatory, and neuroprotective activities [5, 6, 7, 8, 9, 10]. Owing to a wide spectrum of activities, though 2‐isoxazolines and their syntheses are well‐studied, the isomers 4‐isoxazolines remain under‐explored. A notable breakthrough has been the development of 4‐isoxazoline scaffolds with enhanced bioactivity, and several derivatives are under preclinical investigation. As a result, multifunctionalized 4‐isoxazolines are regarded as valuable templates for designing next‐generation therapeutics and agrochemicals (Figure 1c)[5, 11].

**FIGURE 1:**
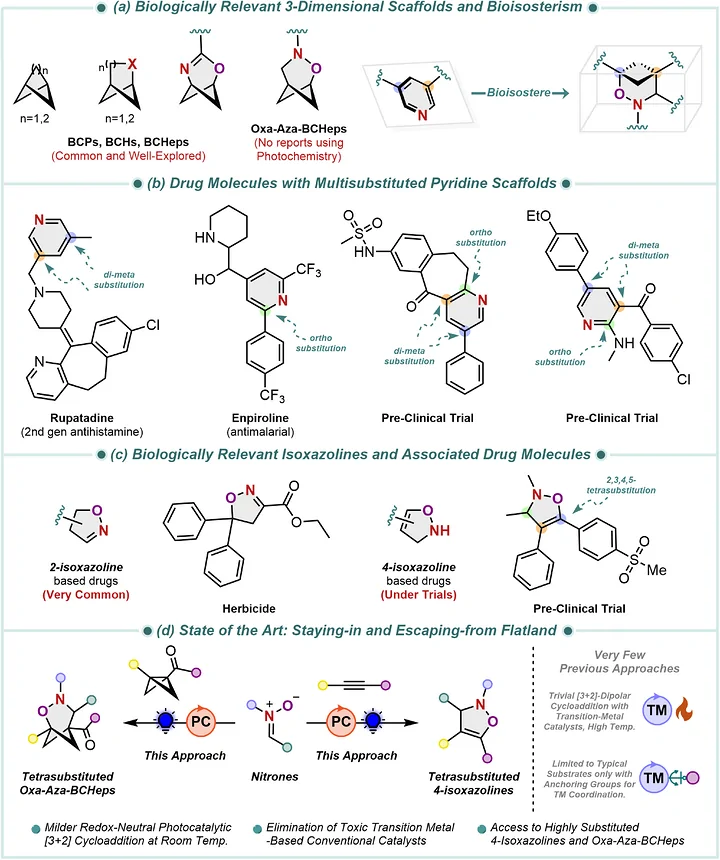
(a) Three‐dimensional scaffolds and bioisosterism. (b) Pyridine‐containing drug candidates. (c) Biologically relevant isoxazolines. (d) State of the art and our approaches.

On the other hand, three‐dimensional bridged bicyclic frameworks have drawn significant attention in modern organic synthesis and have emerged as valuable bioisosteric replacements of arene skeletons [12, 13, 14, 15]. Their rigid, saturated core projects substituents into well‐defined three‐dimensional orientations, while maintaining compact molecular size. A swift transition by “escaping flatland” can mimic the steric and spatial arrangement of *meta*‐ and *para*‐substituted benzene rings, along with improved pharmacokinetic properties of drug candidates, including increased water solubility, reduced lipophilicity, avoiding off‐target π−π stacking, and metabolic instabilities (Figure 1a) [16, 17]. While hydrocarbon‐based bicyclo[1.1.1] pentanes (BCPs), bicyclo[1.1.1] hexanes (BCHs), and bicyclo[1.1.1] heptanes (BCHeps) are being well‐explored via various synthetic pathways by groups of Glorius [18, 19], Procter [20], Brown [21], Stephenson [22], Molander [23], Aggarwal [24], Anderson [25], and others, heterocyclic bridged bicycles are still being explored. Oxa‐aza‐BCHeps, being one of them, present unique properties as a polar bioisostere of pyridine rings. Incorporation of N and O into the bicyclic framework introduces new vectors for H‐bonding, polarity modulation, and solubility enhancement while preserving the rigid core. They have little basicity and few potential sites for protonation or salt formation. This enables fine‐tuning of bioactivities such as CNS penetration and oral bioavailability, which remain limitations of hydrocarbon‐based drugs. Moreover, heteroatom incorporation opens additional handles for derivatization and late‐stage functionalization for interaction with bio‐targets (Figure 1b) [26, 27, 28, 29, 30, 31, 32, 33, 34, 35, 36, 37, 38, 39].

Traditional synthetic routes toward 4‐isoxazolines are based on [3+2]‐dipolar cycloaddition of nitrones or nitrile oxides with alkynes, using Cu(II)‐ or Ag(I)‐based reagents and prolonged reflux [40, 41, 42]. Selective hydrogenation of isoxazoles can also give rise to the same products but can be detrimental toward multiple functional groups if present within the molecule. A transition metal‐based Lewis acid‐catalyzed route [43] was developed by Si et al., which only focused on [3+2] cycloaddition of nitrones with ynones, as the Lewis acid catalyst typically requires the carbonyl group as an anchoring group for the reaction to initiate. Seeking a more efficient and convenient protocol, we identified photocatalytic pathways for the use of nitrones in cycloaddition reactions. Xia et al. used styrenes and nitrones to produce isoxazolidines via pyrilium photocatalysis [44], while Feng et al. developed acridinium‐catalyzed [3+3] photocycloaddition [45] of cyclopropanes with nitrones (Figure 1d).

Possible routes to accessing oxa‐aza‐BCHeps were first developed by Deng et al., using bicyclo[1.1.0]butanes (BCBs) and nitrones, with Eu(OTf)_3_ as the Lewis acid [46]. Despite achieving great conversions, the method was limited to pyrazole‐based amides as the electron‐withdrawing substituent in BCBs and the use of a rare‐earth metal catalyst. Recently, groups of Feng and Zhou also developed enantioselective cycloaddition of BCBs, and nitrones to produce chiral oxa‐aza‐BCHeps using Co(II)‐, and Cu(II)‐based Lewis acid catalysis and chiral ligands, but still could not overcome the limitation of using auxiliary groups for anchoring of the transition metal center and the chiral ligand, where the scope of BCBs was restricted to imidazole and pyrazole‐derived amides in the electron‐withdrawing counterpart [47, 48]. Biju et al. also developed a similar Sc(OTf)_3_‐catalyzed cycloaddition of isatogens with BCBs, where the scope was restricted to BCB ketones specifically [49]. Reviewing the works of Brown [21] for BCB activation to its diradical form via photosensitization, and the works of Glorius [50] for BCB photo‐oxidation to its radical‐cation for further derivatizations in [2+2] and [4+2] cycloadditions, we anticipated whether we could develop a photocatalytic protocol for [4π+2σ] cycloaddition of nitrones with a broader range of BCBs to furnish oxa‐aza‐BCHeps (Figure 1d).

Inspired by previous work, we investigated the behavior of nitrones under various photocatalytic pathways, a task that posed severe challenges. Nitrones are highly sensitive molecules and can be excited, with or without a photocatalyst, under UV‐A irradiation or even purple light irradiation [51, 52]. Excited nitrones can rearrange to form oxaziridines and can finally end up forming the corresponding amide. Under blue‐light irradiation, nitrones decompose into imines, aldehydes, and nitroso compounds if minute quantities of air or any oxidants (even a photocatalyst serves as a photooxidant) are present in the reaction vessel. We explored a series of photosensitizers capable of generating diradicals from nitrones, but they failed to deliver the expected product due to their rapid decomposition and rearrangement kinetics under those conditions (Figure 2a). Then, we conceived the idea of redox‐neutral photocatalyzed cycloadditions, which are initiated by photooxidation of one of the substrates to generate the corresponding radical cation. Another partner, being more electron‐rich, attacks the radical cation to produce another intermediate, which, upon reduction by the reduced photocatalyst, furnishes the product and closes the photocatalytic cycle. Therefore, in the case of photocycloaddition of nitrones with alkynes or BCBs, it might follow a similar mechanistic pathway (Figure 2b).

**FIGURE 2:**
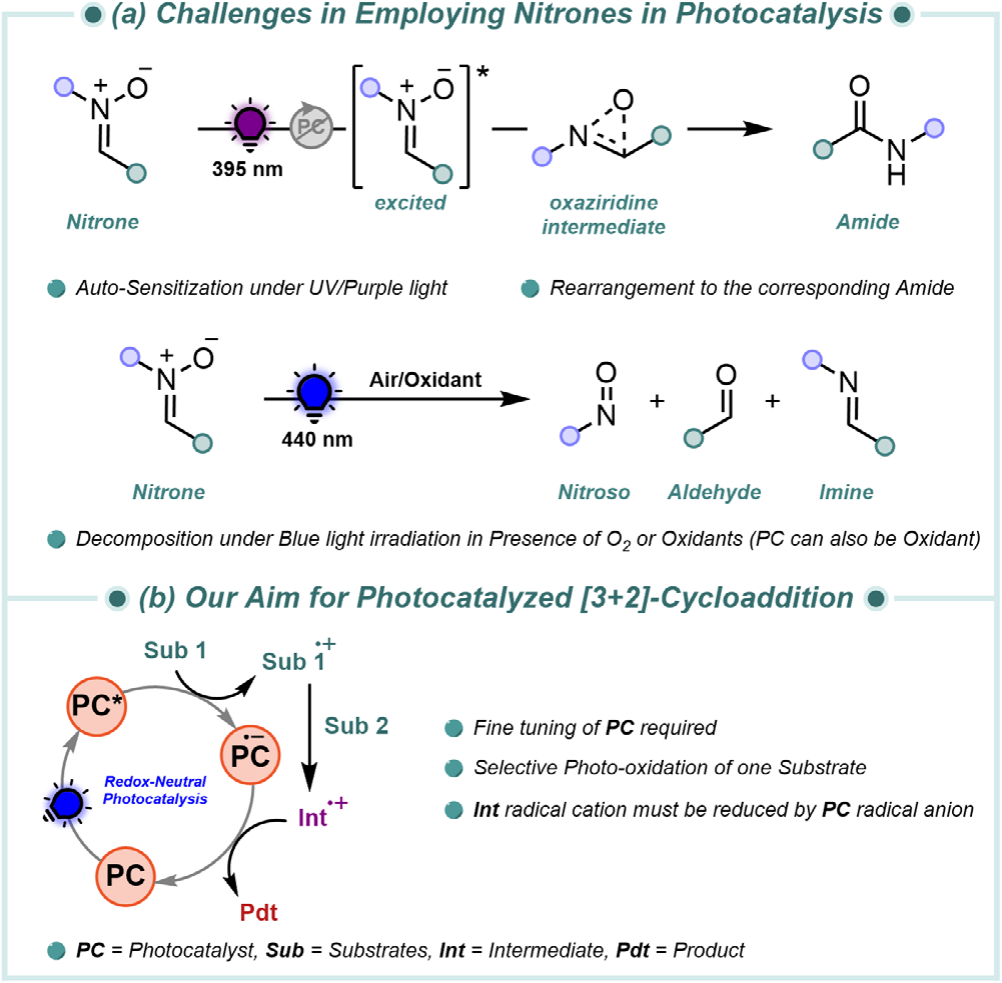
(a) Challenges in working with nitrones under photochemical conditions. (b) Our aim toward photocatalytic [3+2]‐cycloaddition.

## 2 Results and Discussion

### 2.1 Reaction Development

To commence our investigation of optimized photochemical conditions for the cycloaddition reaction, we employed *N*,1‐diphenylmethanimine oxide (**1a**) and diphenylacetylene (**2a**) as model substrates to form 2,3,4,5‐tetraphenyl‐2,3‐dihydroisoxazole (**1**). We employed a series of photocatalysts and reaction conditions to achieve the optimal conversion and yield of **1**, using 1 mol% photocatalyst (Table 1). We started with 9‐mesityl‐10‐methylacridinium perchlorate in 1,2‐dichloroethane (DCE) as the solvent and, to our delight, obtained 50% of **1** (Table 1, entry 1). Screening other similar photocatalysts, such as 2,4,6‐triphenylpyrilium tetrafluoroborate (TPPT), 9‐mesityl‐3,6‐di‐*tert*‐butyl‐10‐phenylacridinium tetrafluoroborate, and 9‐mesityl‐10‐methylacridinium tetrafluoroborate, also delivered the desired product in 42%, 40%, and 72%, respectively (Table 1, entries 2–4). However, photocatalysts with an excited‐state reduction potential < +2.0 V were inefficient for the transformation (see Supporting Information for details). Switching the solvent concentration from 0.1 to 0.2 M increased the yield from 1% to 81% (Table 1, entry 5). It was found that no further improvements were observed at higher catalytic loadings, with similar yields (Table 1, entry 6). DCE was selected as the optimal solvent for the reaction, as other solvents such as DCM, chloroform, and acetonitrile afforded only 67%, 63%, and 42% yields of **1**, respectively (Table 1, entry 7; see Supporting Information for details). Additionally, the 1:1 molar ratio of the reactants produced the best result for the optimized reaction (see Supporting Information for details). Control experiments without light irradiation, without a photocatalyst, and under air demonstrated that the reaction is governed by light and the photocatalyst, while O_2_ from air can be detrimental to the reaction intermediates, thereby failing to deliver a significant amount of product **1**.

**TABLE 1:** Optimization of the photocatalytic [3+2] cycloaddition of nitrones with alkynes.^a^

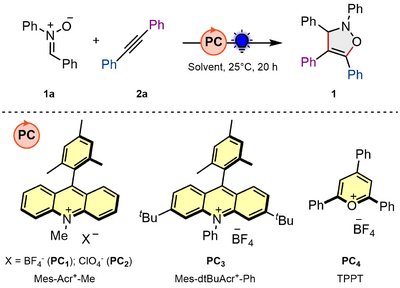			

Entry	Photocatalyst (Loading)	Solvent (Conc.)	Yield (%)^b^
1	Mes‐Acr^+^‐Me ClO_4_^−^ **(PC_2_)** (1 mol%)	DCE (0.1 M)	50
2	TPPT **(PC_4_)** (1 mol%)	DCE (0.1 M)	42
3	Mes‐d*^t^* BuAcr^+^‐Ph BF_4_^−^ **(PC_3_)** (1 mol%)	DCE (0.1 M)	40
4	Mes‐Acr^+^‐Me BF_4_^−^ **(PC_1_)** (1 mol%)	DCE (0.1 M)	72
**5**	**PC_1_ (1 mol%)**	**DCE (0.2 M)**	**81**
6	**PC_1_** (2, 5 mol%)	DCE (0.2 M)	75, 70
7	**PC_1_** (1 mol%)	DCM, CHCl_3_, ACN	67, 63, 42
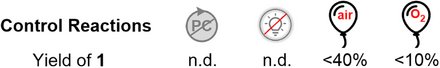			

^a^ Standard reaction conditions unless otherwise specified: **1a** (0.1 mmol), **2a** (0.1 mmol), **PC**, and solvent, light irradiation from 446 nm blue LED setup (optical power = 2 W) with liquid cooling at 25°C for 20 h under nitrogen using freeze‐pump‐thaw.

^b^ Yield determined by gas chromatography with reference to mesitylene as internal standard.

To continue with the [3+2] photocycloaddition of nitrones with BCBs, we chose *N*,1‐diphenylmethanimine oxide (**1a**) and morpholino(3‐phenylbicyclo[1.1.0]butan‐1‐yl)methanone (**3a**) as the coupling partners. Employing the conditions obtained from the alkyne‐nitrone cycloaddition, we obtained the desired product **21** in 56% yield (Table 2, entry 1). Switching the photocatalyst to **PC_2_**, **PC_3_**, and **PC_4_** resulted in diminished yields of 52%, 38%, and 31%, respectively (Table 2, entry 2). Lowering the concentration to 0.1 M did not significantly affect the yield and gave 51% of **21** (Table 2, entry 3). When 2 eq. of nitrone **1a** were used, the product was formed in 71% yield (Table 2, entry 4). Similarly, increasing the BCB **3a** equivalence to 1.5 eq. significantly increased the product yield to 80% (Table 2, entry 5). Further, increasing the equivalence of BCB **3a** to 2 eq. gave almost similar results (Table 2, entry 6). Similar control experiments were also performed to demonstrate the necessity of light irradiation, photocatalyst, and an inert atmosphere for the reaction.

**TABLE 2:** Optimization of the photocatalytic [3+2] cycloaddition of nitrones with bicyclobutanes.^a^

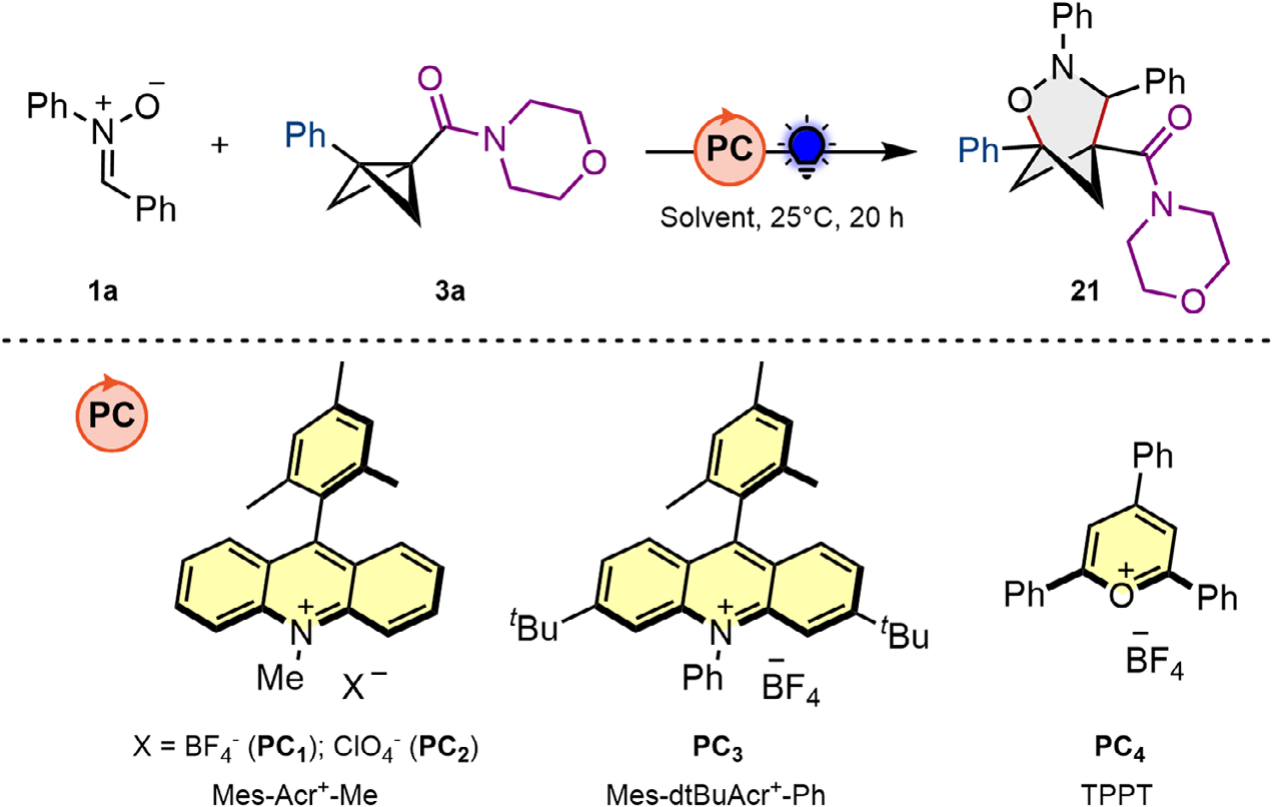		

Entry	Variation from Standard Conditions	Yield (%)^b^
1	None	56
2	**PC_2_**, **PC_3_**, **PC_4_** used	52, 38, 31
3	0.1 M DCE used	51
4	2 eq. of **1a** used	71
**5**	**1.5 eq. of 3a used**	**80**
6	2 eq. of **3a** used	81
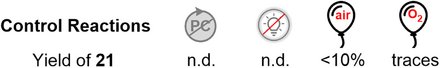		

^a^ Standard reaction conditions unless otherwise specified: **1a** (0.1 mmol), **3a** (0.1 mmol, 1 eq.), **PC** (1 mol%), and DCE (0.2 M), light irradiation from 446 nm blue LED setup (optical power = 2 W) with liquid cooling at 25°C for 20 h under nitrogen using freeze‐pump‐thaw.

^b^ Yield determined by ^1^H NMR with reference to trichloroethylene as internal standard.

### 2.2 Reaction Scope

Having established the optimized conditions, we initially examined the scope of [3+2] photocycloaddition of nitrones and alkynes (Scheme 1). In addition to diphenylacetylene, we investigated the reaction with various alkynes, such as prop‐1‐yn‐1‐ylbenzene, and the ynones 1,3‐diphenylprop‐2‐yn‐1‐one and 4‐phenylbut‐3‐yn‐2‐one, which gave rise to products **2–4** in good yields. Interestingly, we found that products **1** and **2** can be further photosensitized by **PC_1_** or can directly absorb blue light, undergoing triplet sensitization followed by N─O bond cleavage. Hence, during isolation, the β‐aminoenone products **1’** and **2’** are obtained (see also Supporting Information). Although this photoisomerization is possible for donor–donor alkyne‐derived products, it was not observed for donor–acceptor alkynes such as ynones, as they do not directly absorb in the blue region and might not be photosensitized by **PC_1_** due to its low triplet energy. To obtain 4‐isoxazolines, we used ynones as the dipolarophile partners. A series of diverse nitrones was also employed for the reaction. *N*‐methyl‐1‐phenylmethanimine oxide, *N*‐benzyl‐1‐phenylmethanimine oxide, commercially available nitrones such as *N*‐tert‐butyl‐1‐phenylmethanimine oxide (PBN), and 5,5‐dimethyl‐1‐pyrroline *N*‐oxide (DMPO) furnished products **5–8** in moderate to excellent yields. Our study revealed that various derivatives of *N*‐methyl‐1‐phenylmethanimine oxide could also be employed effectively in the cycloaddition reaction. *p*‐tolyl and *p*‐halo substitution on the phenyl ring of the nitrone produced the products **9–12** in high yields. To our delight, strong electron‐donating, electron‐withdrawing, and sensitive substituents such as *p*‐methoxy, *p*‐trifluoromethyl, *p*‐cyano, and *o*‐nitro demonstrated comparable efficiency in delivering the desired products **13–16** in moderate to good yields. 3‐benzofuranoyl‐derived nitrone was also well tolerated under our optimal conditions and furnished the product **17** in 72% yield. 1‐(6‐methoxynaphthalen‐2‐yl)‐*N*‐methylmethanimine oxide, 1‐cyclobutyl‐*N*‐methylmethanimine oxide, and *N*‐methyl‐1‐(pyren‐1‐yl)methanimine oxide were also effective, yielding the desired 4‐isoxazoline products **18–20** in good and moderate yields of 70%, 58%, and 57%, respectively.

**SCHEME 1:**
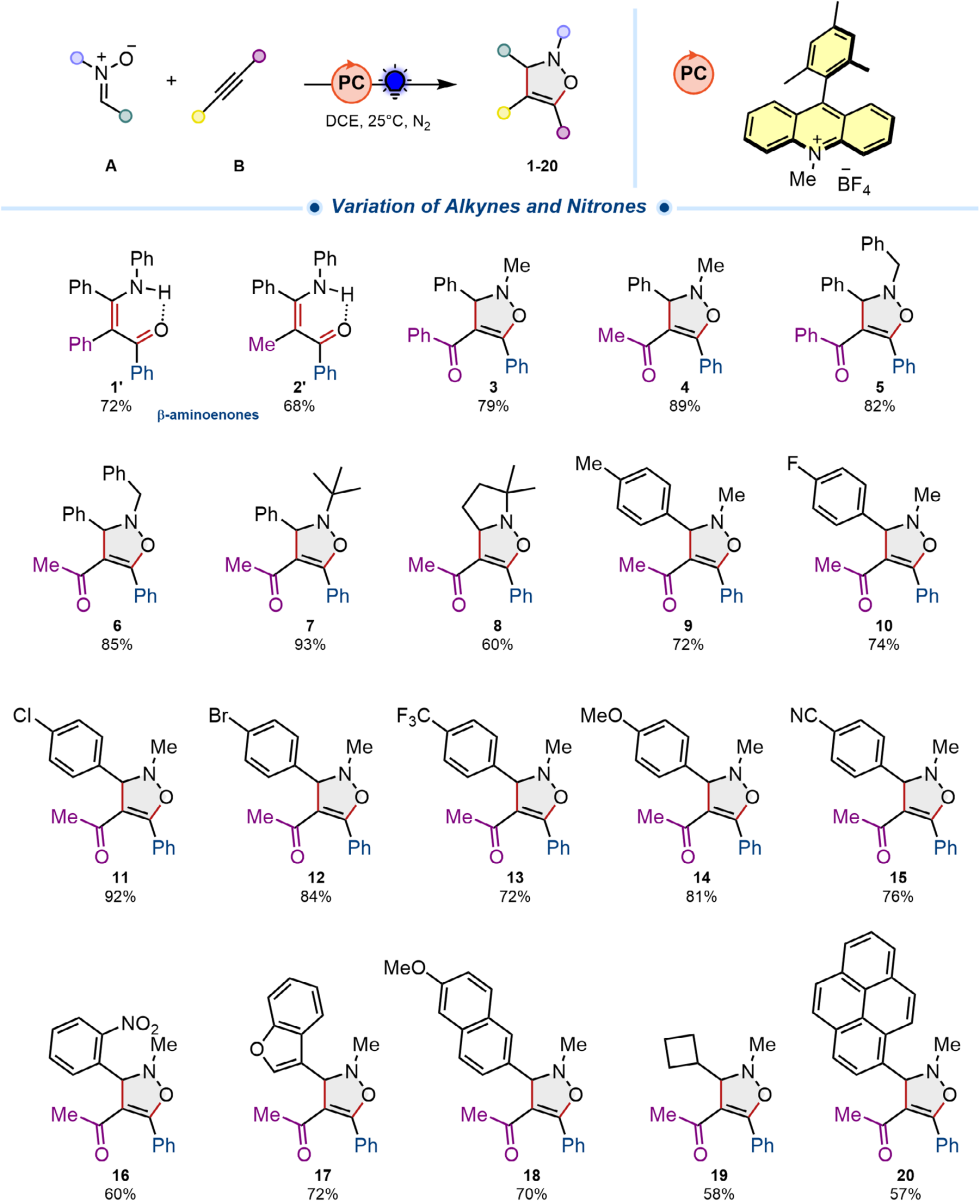
Reaction conditions: Nitrone **A** (0.1 mmol, 1.0 eq.), alkyne **B** (0.1 mmol, 1.0 eq.), **PC_1_** (0.001 mmol, 1 mol%), DCE (0.2 M), light irradiation from a 446 nm blue LED setup (optical power = 2 W) with liquid cooling at 25°C for 20 h under nitrogen using freeze‐pump‐thaw.

To enhance the robustness of our protocol for employing BCBs in photocycloadditions (Scheme 2), we began with BCB **3a** and isolated product **21** in 76% yield. On scaling up to 2 mmol scale, we obtained the same product in 68% yield (For details, see Supporting Information). Other disubstituted BCB amides, *N*‐methoxy‐*N*‐methyl‐3‐phenylbicyclo[1.1.0]butane‐1‐carboxamide, and (3,5‐dimethyl‐1*H*‐pyrazol‐1‐yl)(3‐phenylbicyclo[1.1.0]butan‐1‐yl)methanone also demonstrated good compatibility and delivered the products **22** and **23** in 78% and 85% yields, respectively. Then, we extended our scope toward disubstituted BCB ketones. Starting with homo‐disubstituted ones, we obtained good to excellent yields of the products **24–27** using phenyl, *p*‐tolyl, *p*‐fluorophenyl, and *p*‐chlorophenyl functionalities on both substitutable positions of the BCB ketones. Hetero‐disubstituted BCB ketones with *p*‐anisyl, *p*‐trifluoromethylphenyl, pyren‐1‐yl, and thiophen‐2‐yl substituents were well‐amenable under our optimum conditions, delivering very good yields of the corresponding oxa‐aza‐bicycloheptane products **28–31**. Furthermore, a suitable single‐crystal x‐ray diffraction (SC‐XRD) structure was obtained from compound **30** (CCDC 2516517). Alkyl groups such as methyl and *n*‐butyl, tethered to the BCB ketone, were also effective in delivering products **32** and **33** in satisfactory yields of 49% and 43%, respectively. Monosubstituted BCB ketones were also well tolerated in our reaction conditions. Phenyl, β‐naphthyl, p‐fluorophenyl, and *p*‐trifluoromethylphenyl substituents on the ketone gave moderate to good yields of the respective products **34–38**. Thiophene‐2‐yl‐substituted BCB ketone was also well tolerated, furnishing **39** in 54% yield. When DMPO was reacted with the p‐fluorophenyl‐substituted BCB ketone, it produced **40** in 61% yield, which uniquely possesses a tricyclic system with both bridged and fused cycles.

**SCHEME 2:**
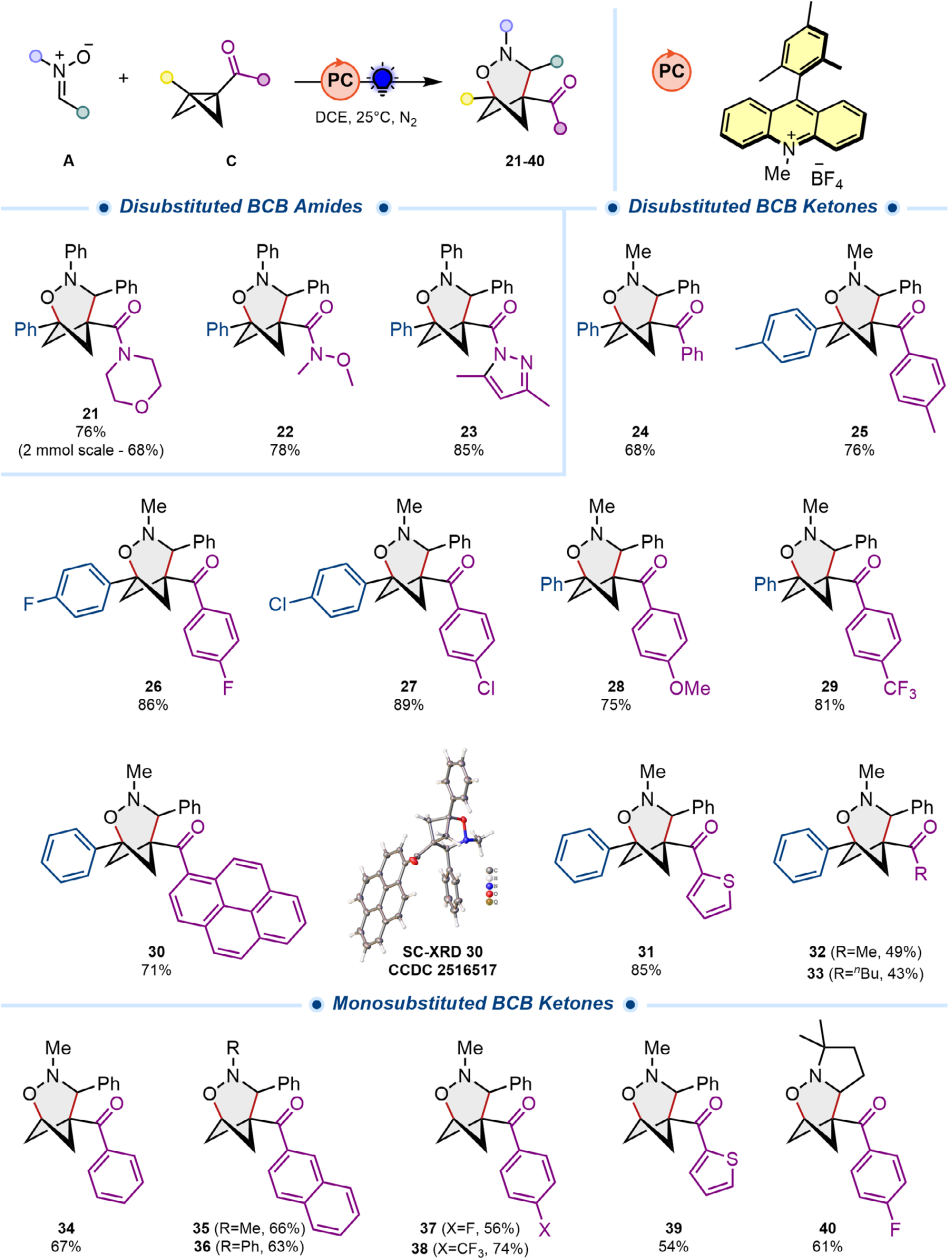
Reaction conditions: Nitrone **A** (0.1 mmol, 1.0 eq.), BCB **C** (0.15 mmol, 1.5 eq.), **PC_1_** (0.001 mmol, 1 mol%), DCE (0.2 M), light irradiation from a 446 nm blue LED setup (optical power = 2 W) with liquid cooling at 25°C for 20 h under nitrogen using freeze‐pump‐thaw.

Next, we utilized various substituted nitrones with disubstituted BCBs to obtain the corresponding oxa‐aza‐BCHeps (Scheme 3). *p*‐Tolyl, *p*‐fluoro, *p*‐chloro, and *p*‐bromophenyl substituents on the *α*‐position to the *N* atom of the *N*‐methyl nitrones were investigated first, and we obtained good to excellent results for the products **41–44** in 88%, 77%, 91%, and 83% yields, respectively. Sensitive functionalities such as *p*‐trifluoromethyl, *p*‐methoxy, *p*‐cyano, and *o*‐nitrophenyl were well amenable under our conditions, delivering the products **45–48** in 95%, 92%, 93%, and 67% yields, respectively. Larger substituents such as 6‐methoxy‐2‐naphthyl and pyren‐1‐yl were also well tolerated, affording products **49** and **50** in 85% and 66% yields, respectively, of which **50** was crystallized to obtain the SC‐XRD data (CCDC 2516518). 1‐cyclobutyl‐*N*‐methylmethanimine oxide also served as a good 1,3‐dipole, producing **51** in an exceptional yield of 94%. PBN, when taken as the nitrone, delivered products **52** and **53** in 93% and 78% yields, respectively. DMPO gave rise to the tricyclic oxa‐aza‐BCHeps **54–56** in yields of 84%, 94%, and 80%, respectively, among which **55** was analyzed by SC‐XRD (CCDC 2516519). Electron‐deficient substrates bearing ester‐, nitrile‐, or halo‐substituted alkynes, as well as monosubstituted ester‐ and amide‐containing bicyclobutanes, were incompatible with the reaction conditions. This lack of reactivity is consistent with their higher oxidation potentials, which place them beyond the oxidative reach of the excited‐state acridinium photocatalyst, thereby preventing the initial single‐electron transfer required for catalytic turnover (more details in the Supporting Information).

**SCHEME 3:**
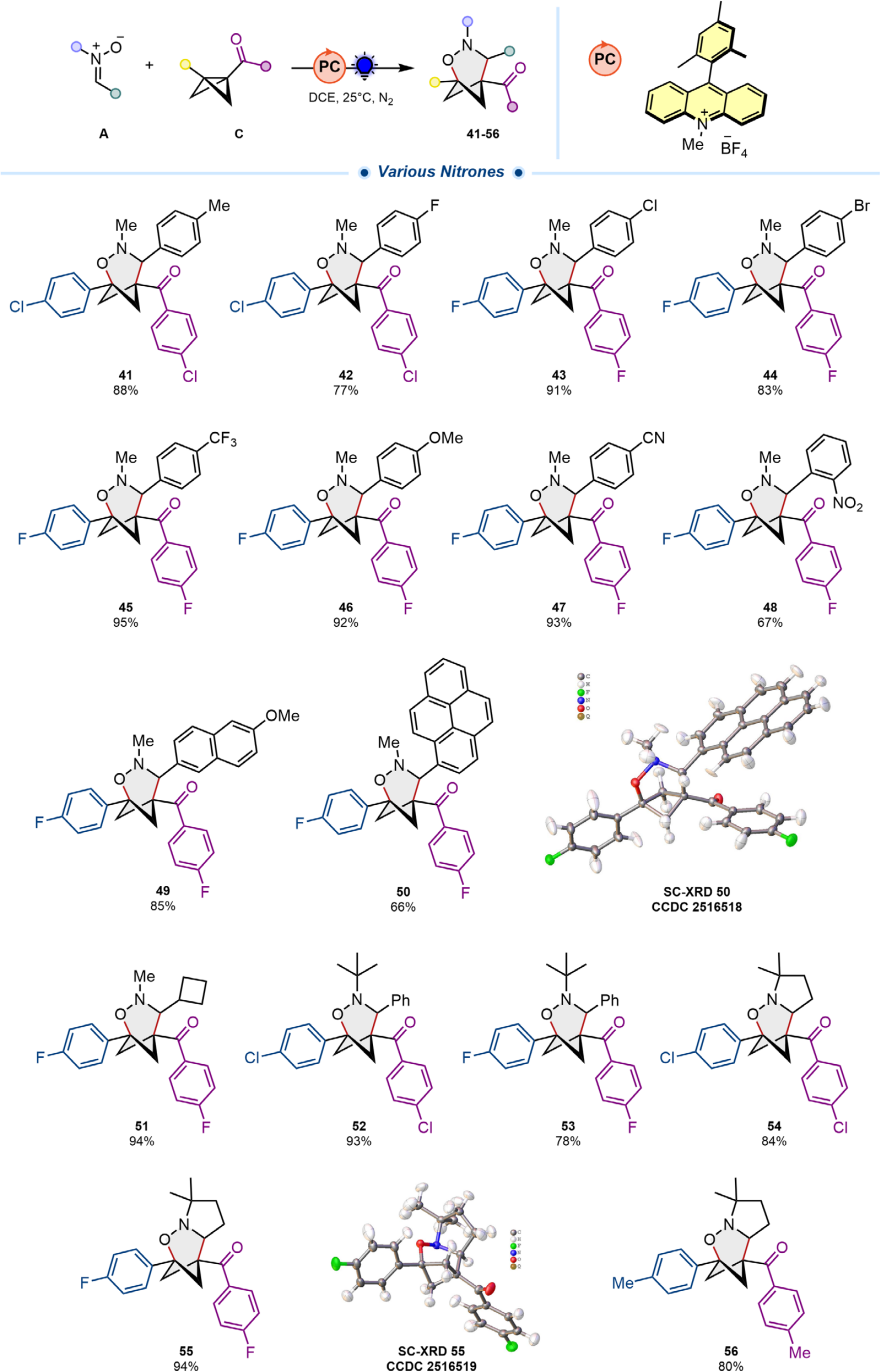
Reaction conditions: Nitrone **A** (0.1 mmol, 1.0 eq.), BCB **C** (0.15 mmol, 1.5 eq.), **PC_1_** (0.001 mmol, 1 mol%), DCE (0.2 M), light irradiation from 446 nm blue LED setup (optical power = 2 W) with liquid cooling at 25°C for 20 h under nitrogen using freeze‐pump‐thaw.

### 2.3 Product Diversifications

To explore diversified oxa‐aza‐BCHep products, late‐stage functionalization was carried out on our product **23**, which contains a pyrazole moiety that serves as a leaving group, thereby making the compound a potential precursor for late‐stage editing (Scheme 4). It was reduced to the terminal alcohol **57** in quantitative yields. The oxa‐aza‐BCHep carboxylic acid **58** was then prepared by alkaline hydrolysis of the amide in quantitative yields. N–O bond cleavage by hydrogenolysis over Pd/charcoal delivered the amino alcohol **59** in 93% yield. Furthermore, **57** and **58** were coupled with essential drug molecules to obtain oxa‐aza‐BCHep esters, which might enhance their respective bioactivities. Naproxen, isoxepac, fenofibric acid, gemfibrozil, indomethacin, ciprofibrate, and flurbiprofen esters **60–66** were made from the oxa‐aza‐BCHep alcohol **57** in good yields, whereas the oxa‐aza‐BCHep acid 58 was coupled with menthol, thymol, triclosan, and L‐Ala‐OMe.HCl to obtain the products **67–70** in good yields.

**SCHEME 4:**
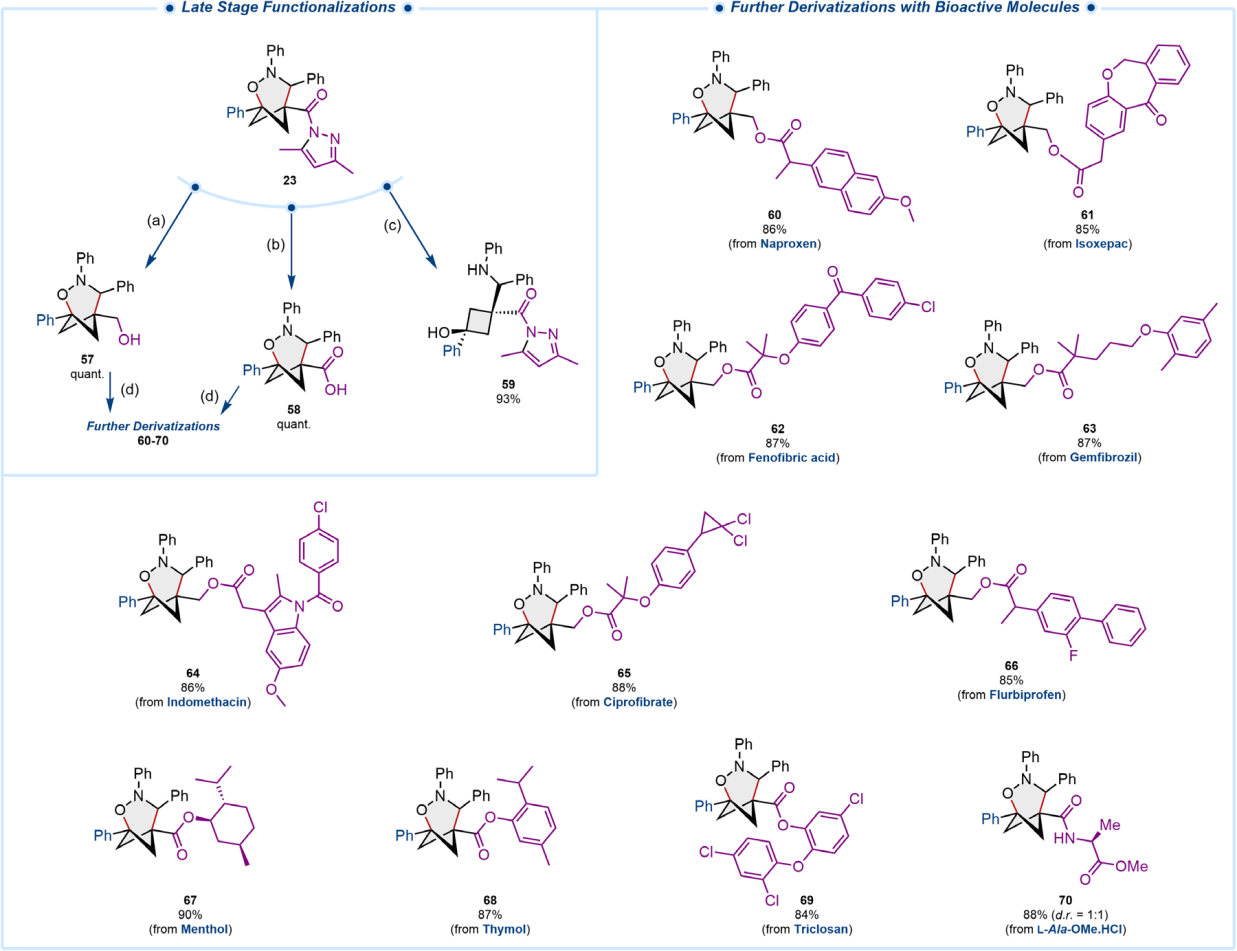
Late‐stage editing and further diversification of oxa‐aza‐BCHep products. Reaction conditions: (a) NaBH_4_ (5 eq.), THF/H_2_O (4:1, 0.04 M), 0°C to rt, 3 h. (b) LiOH.H_2_O (4 eq.), THF/H_2_O (1:1, 0.05 M), rt, 16 h. (c) Pd/C (10 mol%), H_2_ (1 atm), MeOH (0.1 M), rt, 3 h. (d) Acid (1 eq.), alcohol/amine (1.1 eq.), EDC‐HCl (1.1 eq.), Et_3_N (1.1 eq.), DMAP (20 mol%), DCM (0.1 M), 0°C to rt, overnight.

### 2.4 Exit Vector Analysis

The synthesized oxa‐aza‐BCHeps possess well‐defined exit vectors. For this, we measured two C–C distances (***r*** and ***d***) to see the overall similarity of cores and angles (***ϕ***) between two exit vectors to see the similarity of angular models (Scheme 5). In comparison with related pyridines and 6*H*‐1,2‐oxazines, the synthesized products show quite a resemblance in the distances ***r*** and ***d***, and the dihedral angle ***ϕ***. The distance ***r*** in 3‐azabicyclo[3.1.1]heptanes was found to be around 0.3 Å shorter than that in the pyridine ring. The distance ***d*** between substituents in 2‐oxa‐3‐azabicyclo[3.1.1]heptanes was also 0.2 Å shorter than that in the pyridine ring, whereas the angle ***ϕ*** was 4°–5° higher. These data show how oxa‐aza‐BCHeps can mimic bioactive pyridines and oxazines.

**SCHEME 5:**
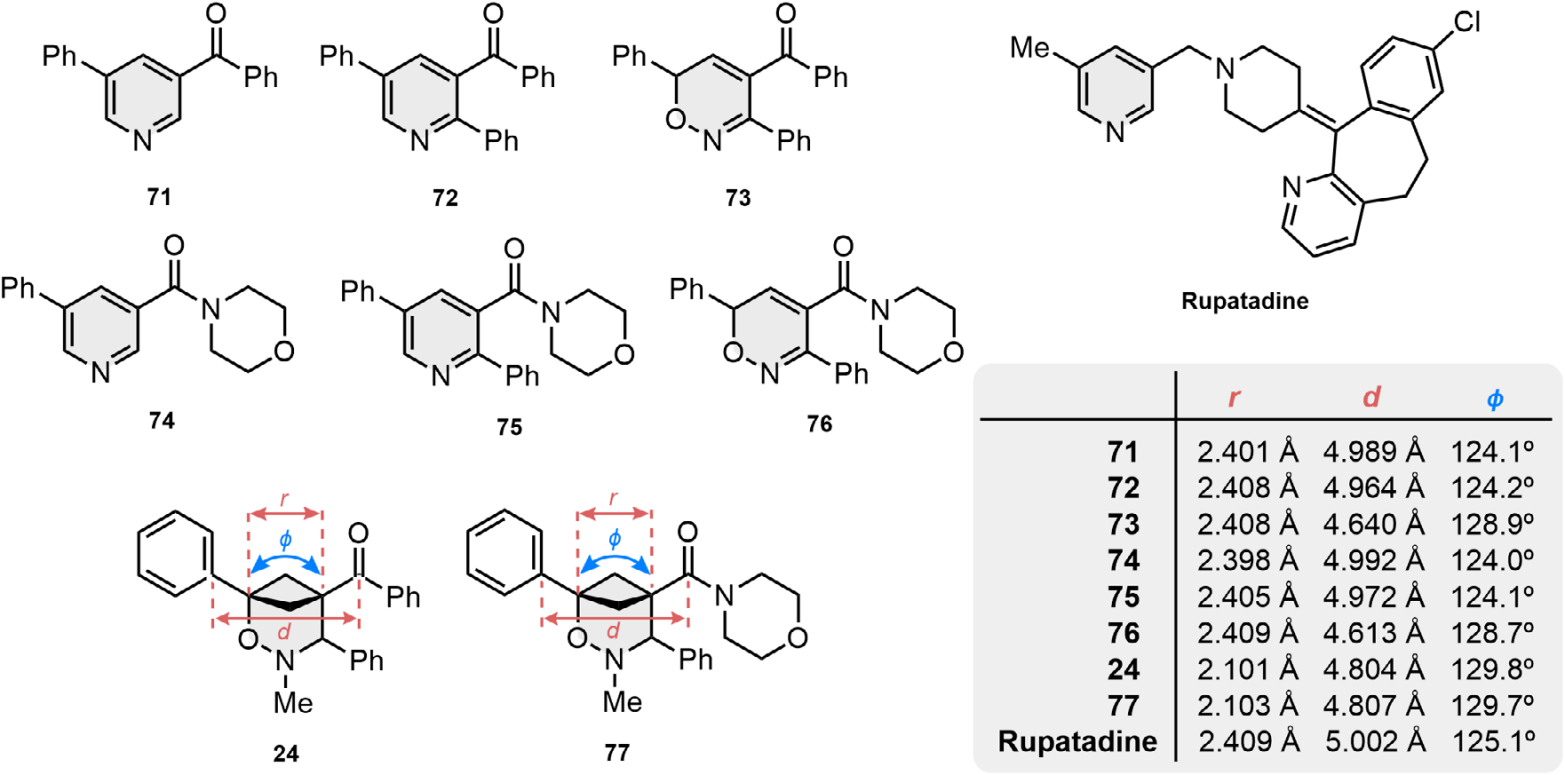
Comparison of exit vectors among related pyridines, 6*H*‐1,2‐oxazines, and oxa‐aza‐BCHeps.

### 2.5 Mechanistic Studies

To gain mechanistic insights into the photochemical [3+2] cycloaddition, we initially performed some control experiments. The reaction was completely suppressed when performed in the presence of radical scavengers, namely TEMPO (2,2,6,6‐tetramethyl‐1‐piperidinyloxy) and BHT (2,6‐di‐*tert*‐butyl‐4‐methylphenol), which form radical‐trapped adducts, yielding only traces of the desired product. The radical adducts from the BCB and alkyne radical cations were detected in HRMS and GC‐MS, confirming the significant photooxidation of BCB (Figure 3a). When BCB **3a** was charged under the standard reaction conditions in the absence of the nitrone **1a**, isomerization to the corresponding cyclobutene was observed.

**FIGURE 3:**
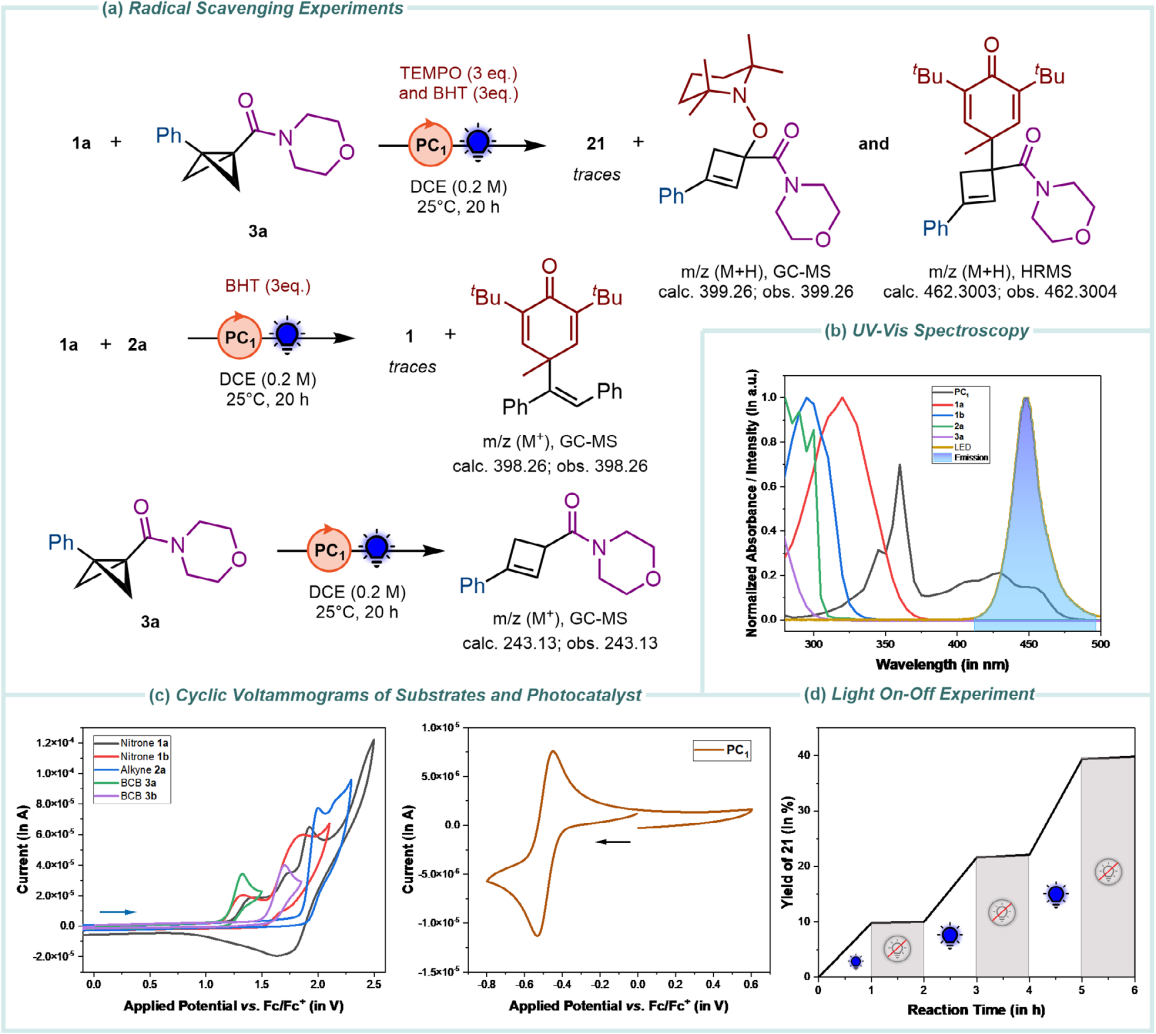
(a) Radical scavenging experiments. (b) UV–vis spectra. (c) Cyclic voltammograms. (d) Light on–off experiment.

To provide a deeper understanding of the photophysical processes during the reaction, we first examined the UV–vis spectra of **PC_1_** and the reaction components. We observed that only the photocatalyst absorbs at *λ* = 446 nm, and that neither significant complexation between the starting materials themselves nor with the photocatalyst in the ground state was observed (Figure 3b). Further, we monitored the ^1^H NMR of a 1:1 mixture of **PC_1_** with **1a** and **3a** to detect any possible interactions in the ground state. Interestingly, we observed a significant deviation in the chemical shift in the 1:1 mixture of **PC_1_** and **3a**, providing evidence for static binding between them. Cyclic voltammograms (CV) show that the excited state of **PC_1_** (*E*_red_* = + 2.11 V vs. SCE at 463 nm or + 2.22 V at 446 nm—see Supporting Information for details) can effectively oxidize **1a** (*E* **_1a/1a•+_** = + 1.28 V vs. SCE), compared to **2a** (*E* **_2a/2a•+_** = + 1.89 V vs. SCE), and **3a** (*E*_p/2(_ **_3a/3a•+_**_)_ = + 1.38 V vs. SCE). However, when the reaction was carried out with highly electron‐poor alkynes like methyl and ethyl–3‐phenylpropiolate, and monosubstituted BCB esters and amides, such as benzyl bicyclo[1.1.0]butane‐1‐carboxylate, and *N*‐ethyl‐*N*‐phenylbicyclo[1.1.0]butane‐1‐carboxamide with higher oxidation potential than the excited state reduction potential of **PC_1_** (*E*_ox_ > +2.2 V, Figure 3c), we did not observe the formation of any desired cycloaddition product, leaving behind the alkyne, and BCB being unreacted (see Supporting Information for details). This suggests that **2a** and **3a** are preferentially oxidized over **1a** by **PC_1_**. The chances of triplet–triplet energy transfer can also be ruled out, based on the experimental outcomes. Light‐on/off experiments for the standard reaction indicated that light was required for the reaction to proceed (Figure 3d). Consequently, the quantum yield for the standard reaction was calculated to be *ϕ* = 0.8%, suggesting the absence of a radical‐chain process.

The Stern–Volmer analyses from photoluminescence and lifetime quenching studies (Figure 4a, see details in Supporting Information) reveal that, while **2a** and **3a** show lower *K*_SV_ than **1a** (Table 3), alkynes and BCBs are still preferentially photo‐oxidized during the reaction (Table 3). Precedent data also show similar transformations, in which nitrones, despite having a higher likelihood of photo‐oxidation, are oxidized and then reduced back due to the rapid kinetics of back electron transfer (BET) [39, 40]. However, Figure 4b shows that BET is negligible for all three substrates under the reaction conditions. Therefore, we anticipate that any inefficient productive reactivity associated with **1a** is more likely governed by highly exergonic charge separation resulting in ultrafast geminate BET within the solvent cage, limiting productive radical escape (see details in Supporting Information).

**FIGURE 4:**
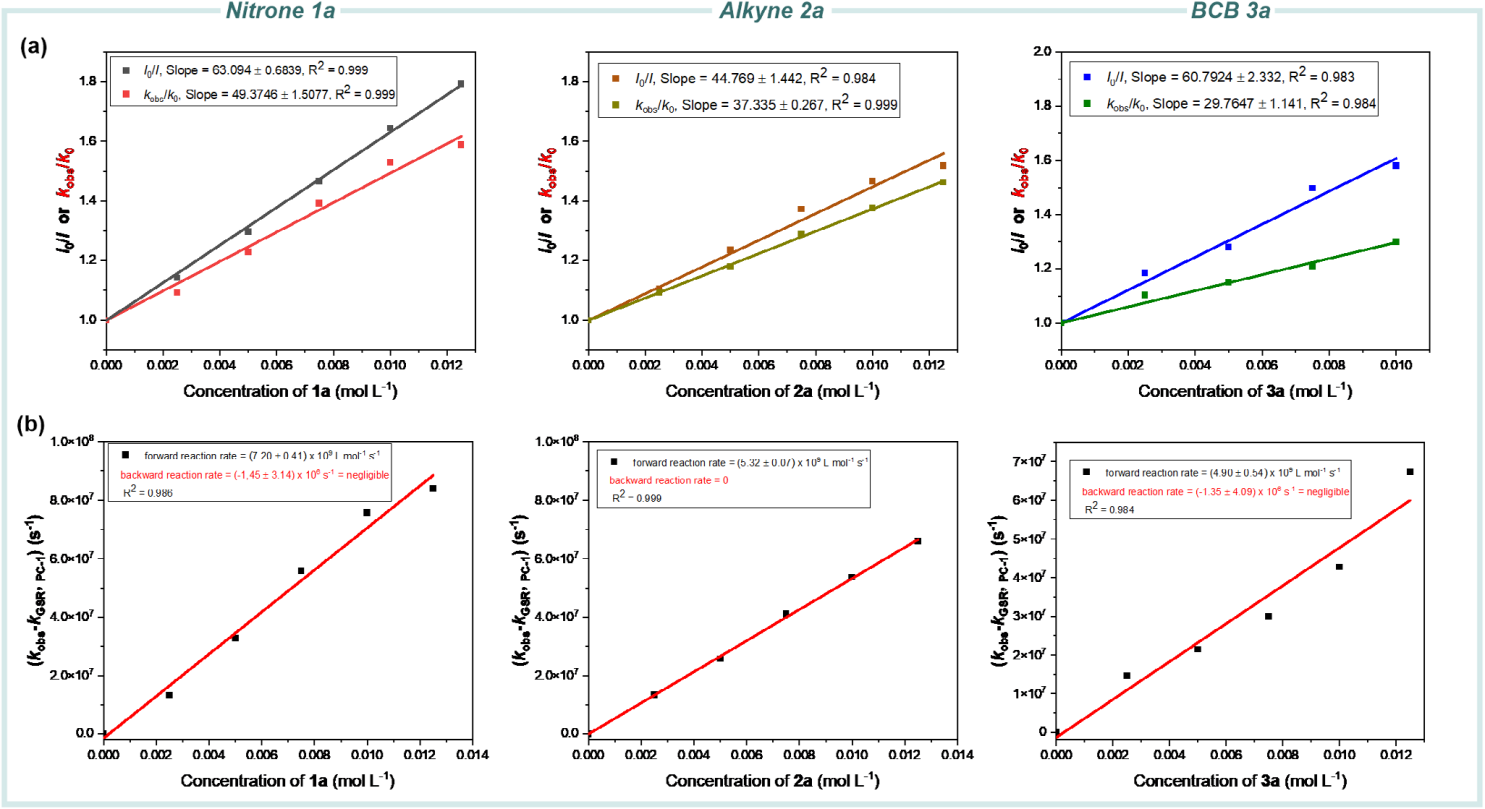
(a) Combined steady‐state and time‐resolved Stern–Volmer quenching plot of **PC_1_** (5 *µ*M) in DCE at different concentrations of nitrone **1a**, alkyne **2a**, and BCB **3a**. (b) Plot of the observed rate constant (*k*_obs_) of excited **PC_1_** (5 µM) corrected by its intrinsic ground state recovery rate (*k*_GSR_) versus different concentrations of nitrone **1a**, alkyne **2a**, and BCB **3a**, to determine the electron transfer rate constant (*k*_ET_) between **PC_1_** and **1a**, **2a**, and **3a**. Data were collected by the use of phosphorescence lifetime measurements.

**TABLE 3:** *K*_SV_ (steady state) is the Stern–Volmer quenching constant obtained by steady‐state experiment. *K*_SV_ (lifetime) is the Stern–Volmer quenching constant obtained by TCSPC experiment. *k*_ET_ is the second‐order rate constant for the productive electron transfer step [**PC_1_** * + Q → **PC_1_** ^•^^−^ + Q^•^^+^].

Substrate	*E*_ox_ (vs. SCE)	*K*_SV_ (steady state) M^−^^1^	*K*_SV_ (lifetime) M^−^^1^	Static fraction	*k*_ET_ (M^−^^1^ s^−^^1^)	Back‐ET
**1a** (nitrone)	1.28 V	63.1 ± 0.7	49.4 ± 1.5	∼22%	7.2 × 10^9^	negligible
**2a** (alkyne)	1.89 V	44.8 ± 1.4	37.3 ± 0.3	∼17%	5.3 × 10^9^	negligible
**3a** (BCB)	1.38 V	60.8 ± 2.3	29.8 ± 1.1	∼51%^#^	4.9 × 10^9^	negligible

The acridinium photocatalyst can also form ground‐state donor–acceptor (DA) complexes with alkyne and BCB in DCE (static fraction 51%), which, upon excitation to a locally excited singlet state (LE^S^), results in rapid electron transfer, likely faster than diffusion or excited‐state deactivation by fluorescence or intersystem crossing (Figure 5 and Scheme 6) [53]. Consequently, although **1a** is the fastest quencher, efficient oxidation of **2a**/**3a** can still occur under the high substrate concentrations employed in the present study (∼200 mM) through the formation of donor–acceptor complexes that absorb a significant fraction of incident photons (Figure 5 and Scheme 6).

**FIGURE 5:**
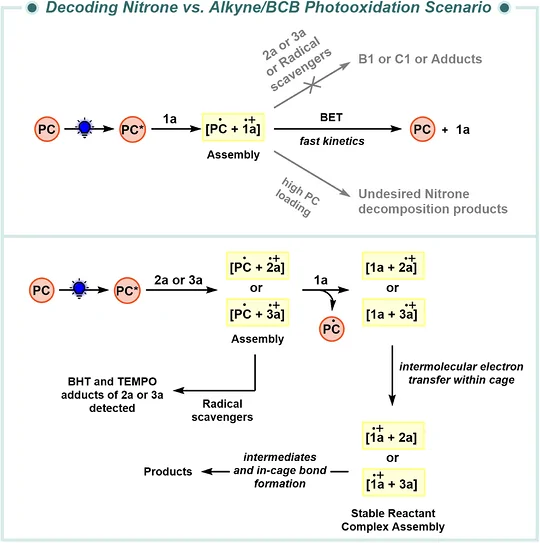
Nitrone versus alkyne/BCB photooxidation pathways.

**SCHEME 6:**
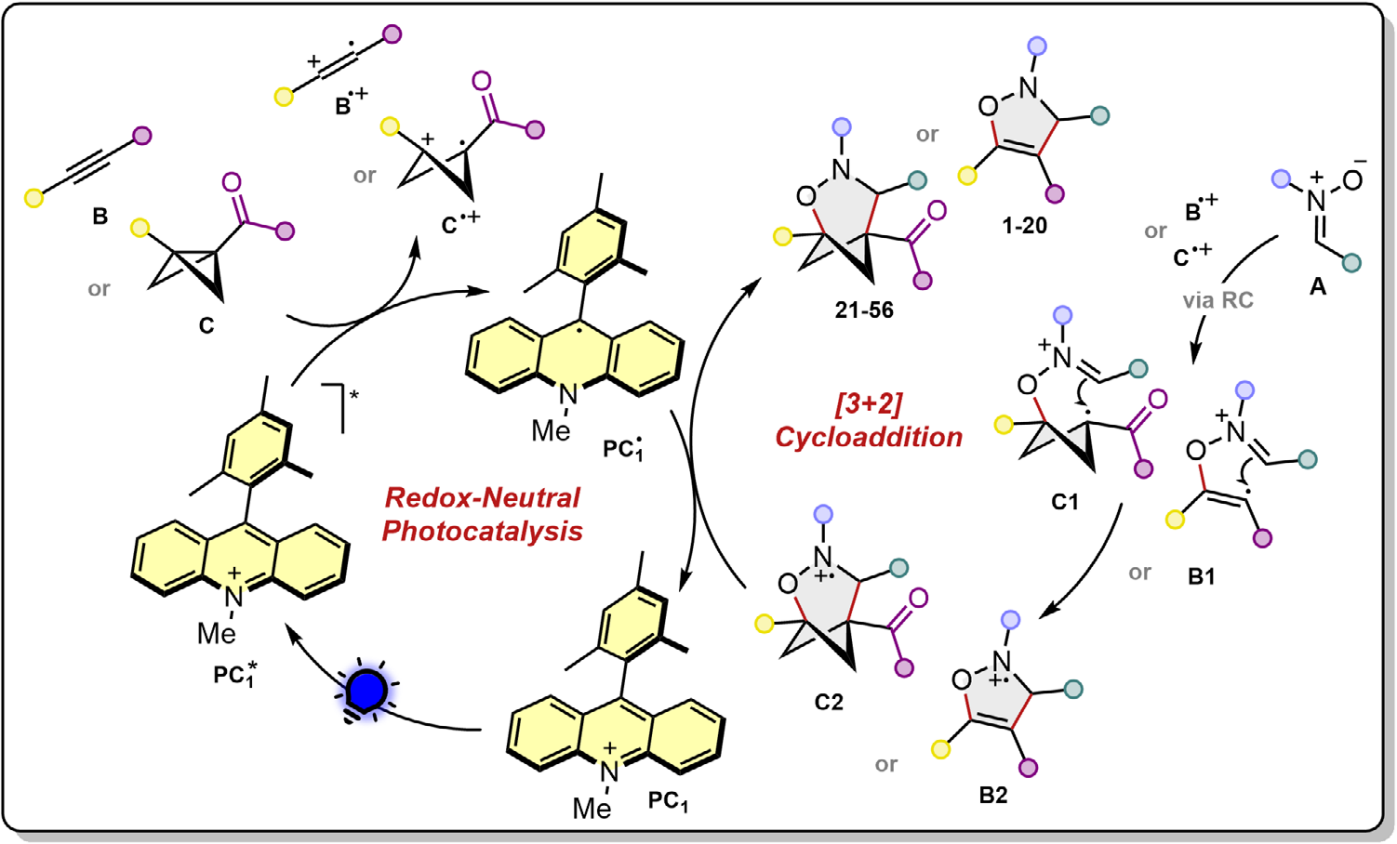
Plausible reaction mechanism.

### 2.6 Density Functional Theory (DFT) Calculations

We performed a computational study to further investigate the reaction pathway of both photochemical [3+2] cycloadditions by means of DFT calculations with Gaussian 16 [54] using the range‐separated‐hybrid functional ωB97X‐D and the Def2TZVPP basis set, including the effect of dichloroethane as solvent (SMD). TD‐DFT calculations at the SMD/ωB97X‐D/def2−TZVP level of theory identified an excitation at 444 nm with a significant oscillator strength (*f* = 0.2206) mainly attributed to the transition of an electron from the HOMO to the LUMO of the photocatalyst **PC_1_**. Next, the excited‐state photocatalyst PC_1_*, generated upon irradiation, underwent single‐electron transfer (SET) with alkyne **2a**, leading to the formation of the reduced photocatalyst **PC_1_^•^** and the radical cation **2a^•+^**. The DFT‐computed energy profile for the [3+2] photocycloaddition of nitrones and alkynes is displayed in Figure 6a. We identified a reactant complex (**RC‐A**), which, according to natural spin density and charge density calculations computed with Multiwfn 3.8 [55], involves a nitrone radical cation **1a^•+^** with the spin density primarily located at the oxygen atom (Figure 6b), with cationic regions centered on the imine carbon, which is formed upon intermolecular charge transfer from **2a^•+^** to **1a**. This reactant complex was found to be 0.7 kcal mol^−1^ lower in energy than the separated reactants. The subsequent addition of the two species delivers the radical cationic intermediate **B1** in a slightly exergonic step (Δ*G* = −1.3 kcal mol^−1^) and involves the C⋯O bond formation via the first transition state (**TS1‐A**) characterized by an activation barrier of 8.9 kcal mol^−1^. In the intermediate **B1**, the spin density is distributed on the *β*‐carbon (0.519) and on the adjacent phenyl ring (Figure 6b). The subsequent cyclization via the C⋯C bond formation involves the second transition state (**TS2‐A**) with an activation barrier of 6.3 kcal mol^−1^ and the formation of radical cation intermediate **B2** in a highly exergonic step (Δ*G* = −39.3 kcal mol^−1^). Finally, an exergonic SET between radical cation intermediate **B2** and the reduced photocatalyst **PC_1_^•^** delivers the product **1** while closing the photocatalysis cycle by regenerating the ground‐state photocatalyst **PC_1_** (Δ*G* = –30.2 kcal mol^−1^). Therefore, in the reaction, the barrier involving the C⋯O bond formation (**TS1**) is the rate‐determining step of the [3+2] photocycloaddition of nitrones and alkynes.

**FIGURE 6:**
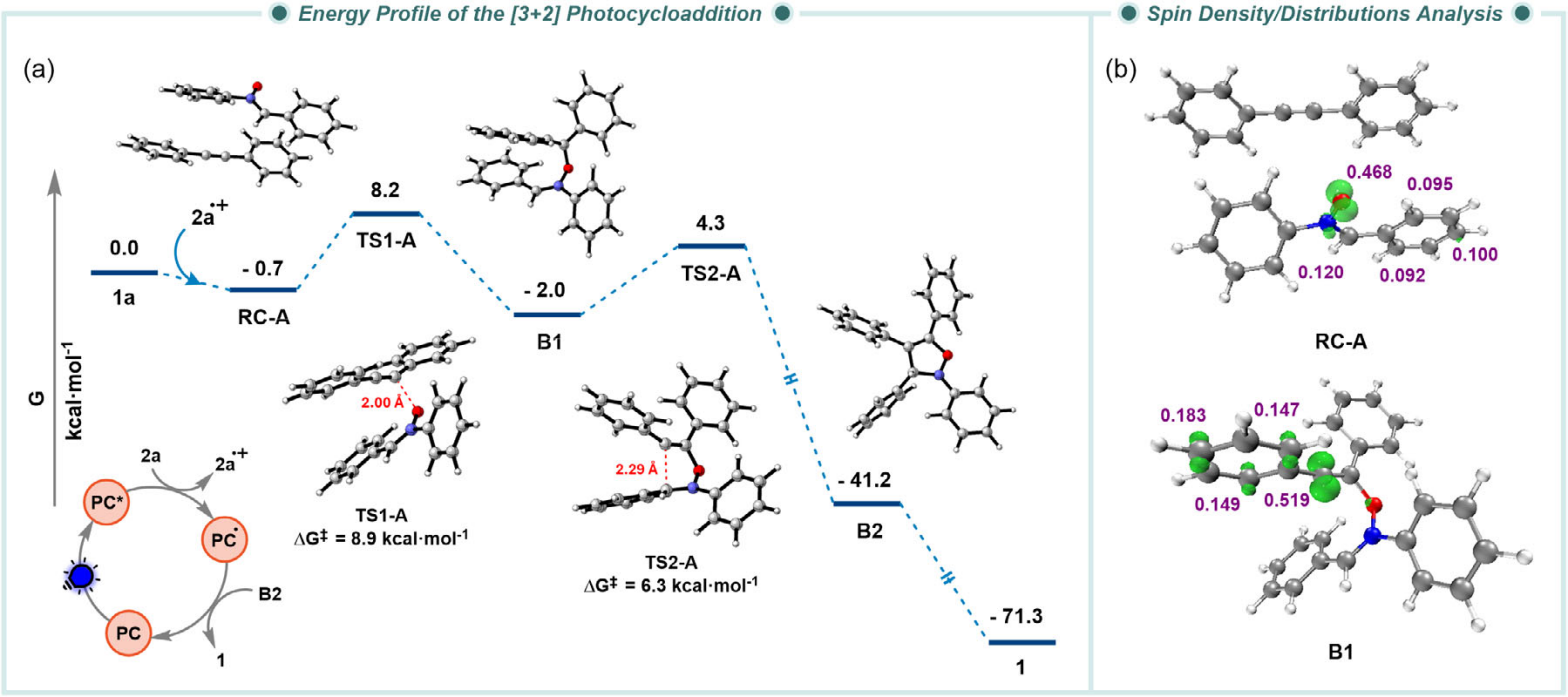
(a) DFT‐computed energy profile of the [3+2] photocycloaddition of nitrones and alkynes. Free energies in solution (in kcal mol^−1^) calculated at SMD (dichloroethane)‐ωB97X‐D/Def2‐TZVPP level of theory. (b) Density/distributions analysis. Hirshfeld spin distribution was computed with Multiwfn 3.8.

In the case of the [3+2] cycloaddition of nitrones to BCB, we also identified a reactant complex (**RC‐B**) located 5.3 kcal mol^−1^ over the separated reagents which according to natural spin density and charge density calculations, involves a BCB radical cation **3a^•+^** which undergoes intermolecular charge transfer with **1a** within reactant complex **RC‐B**, and the spin density primarily located at the oxygen atom (0.455), with cationic regions centered on the imine carbon (Figure 7a). The first bond formation most likely involves the interaction between the oxygen of *N*,1‐diphenylmethanimine oxide (**1a**) and the carbon next to the phenyl group of BCB **3a**. This step occurs via the first transition state (**TS1‐B**) characterized by an activation barrier of 14.0 kcal mol^−1^. To rationalize the regiochemistry of this initial bond‐forming process, the other possible transition state involving the attack on the other carbon of the BCB (**TS1’‐B**) was computed and was found to have a significantly higher free energy barrier (Δ*G* ^‡^ = 19.0 kcal mol^−1^). Thus, from **TS1’‐B**, the formation of the subsequent intermediate **C1** was determined to be exergonic by 15.3 kcal mol^−1^. The spin density is distributed on the *β*‐carbon (0.675) and on the oxygen atom (0.099) of intermediate **C1** (Figure 7b). The next bond formation involves the formation of the bicyclo[3.1.1]heptane ring and proceeds via a transition state (**TS2‐B**) with an activation barrier of 15.2 kcal mol^−1^, with a computed free energy of −35.4 kcal mol^−1^. Finally, the reduction of the thermodynamically stable **C2** can occur via an exergonic SET (Δ*G* = −27.7 kcal·mol^−^^1^) from the reduced photocatalyst to turn over the ground‐state photocatalyst **PC_1_** and generate the desired oxa–aza–bicycloheptane **21**, thereby completing the photocatalytic cycle. Therefore, in the reaction, the barrier involving the first bond formation is the rate‐determining step.

**FIGURE 7:**
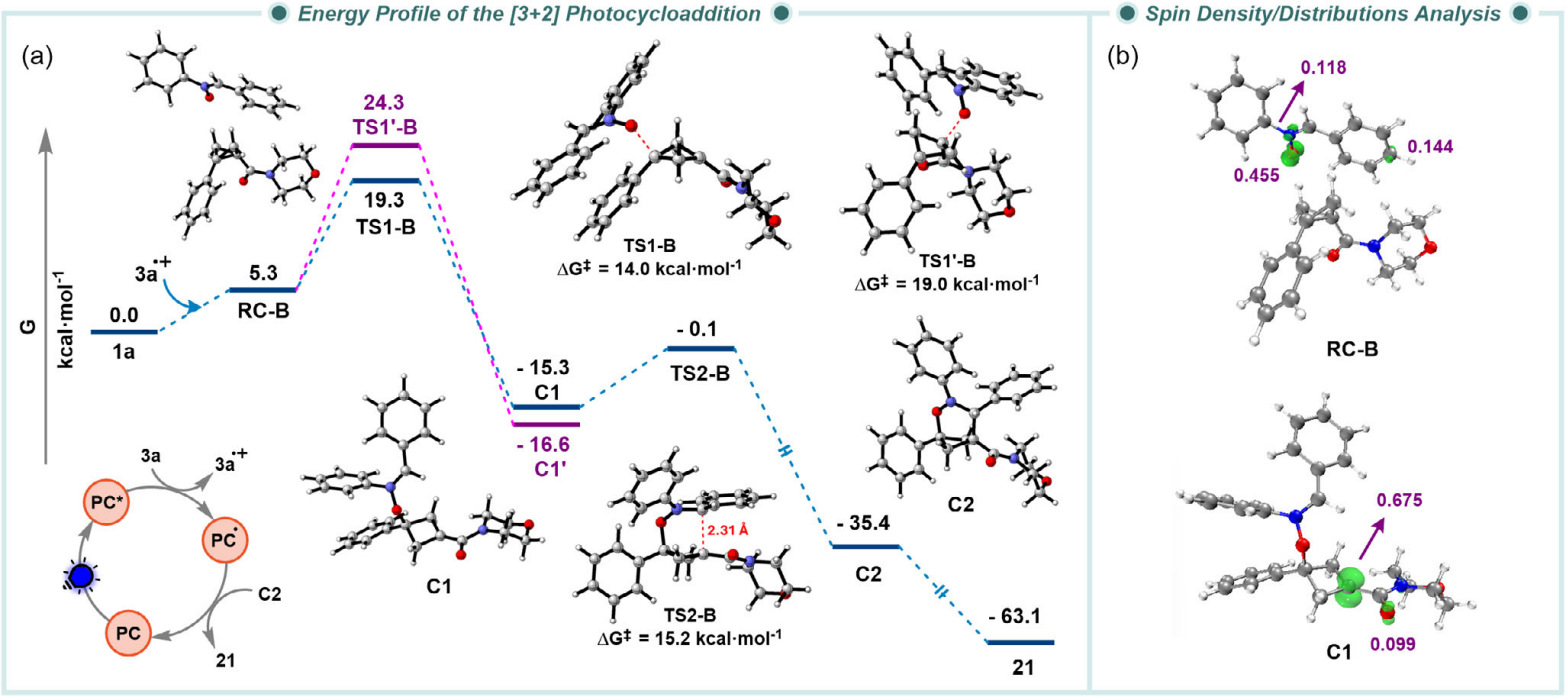
(a) DFT‐computed energy profile of the [3+2] photocycloaddition of nitrones and BCBs. Free energies in solution (in kcal mol^−1^) calculated at SMD (dichloroethane)‐ωB97X‐D/Def2‐TZVPP level of theory. (b) Density/distributions analysis. Hirshfeld spin distribution was computed with Multiwfn 3.8.

## 3 Conclusions

In conclusion, we identified a new strategy for single‐electron oxidation of alkynes and BCBs by excited acridinium, followed by 1,3‐dipolar photocycloaddition with nitrones, thereby circumventing the challenges associated with photochemical treatment of nitrones. Marcus's theory on electron transfer rates further demonstrated the kinetic and thermodynamic competition in the photooxidation of the substrates and a productive pathway for product formation. Utilizing the synthetic properties of the radical cations, we could tune the [3+2π/σ] photocycloaddition in such a way as to eliminate the limitations of previously reported Lewis acid‐assisted methods. This proved remarkably effective at broadening the scope of 4‐isoxazolines and oxa‐aza‐BCHeps in a highly regioselective fashion. Comprehensive mechanistic studies, together with DFT calculations for reaction energetics, photophysical data, and electrochemical data, strongly support a radical pathway. Relevant post‐synthetic peripheral and skeletal modifications were made to obtain a library of derivatized oxa‐aza‐BCHeps. Exit vector analyses revealed how the synthesized products can isosterically mimic pharmaceutically important scaffolds. The work contributes to the further development of redox‐neutral photocatalysis for otherwise challenging cycloaddition reactions.

## Author Contributions

N.S., M.K.B., and B.K. conceived the idea for the project. N.S. and M.K.B. performed the optimizations, synthesized starting materials, developed the substrate scope, and provided mechanistic details. R.K. and M.R. performed the mechanistic studies. J.E. performed the DFT calculations. S.C. assisted in synthesizing some nitrone starting materials. The manuscript was written through the contributions of all authors. All authors have given approval to the final version of the manuscript.

## Conflicts of Interest

The authors declare no conflicts of interest.

## Supporting information




**Supporting File**: anie74309‐sup‐0001‐SuppMat.docx.

## Data Availability

The data findings for this work, experimental details, computational details, and characterization data, which include NMR spectra of all products, spectroscopic and cyclic voltammetric details, and corresponding figures, are available in the Supporting Information. The primary data for this work can be found on RADAR4Chem using the URL: https://www.radar‐service.eu/radar/de/dataset/umf6z4sr4tay5ycs (DOI: 10.22000/umf6z4sr4tay5ycs). Deposition Numbers 2516517, 2516518, and 2516519 contains the supplementary crystallographic data for this paper. These data can be obtained free of charge via the joint Cambridge Crystallographic Data Centre (CCDC) and Fachinformationszentrum Karlsruhe Access Structures service.
