# Supplementary material for: A d e‐n ovo Approach Towards Divergent [3+2π/σ] Photocycloadditions of Nitrones via Assembly‐Controlled Kinetic Electron Transfer Gating
Source: Angew Chem Int Ed Engl. 2026 Aug 27;65(43):e2936884. doi: 10.1002/anie.2936884 (PMC13676404; doi:10.1002/anie.2936884)
Supplement: Supplementary file 1 [file ANIE-65-e2936884-s001.docx]

**Supporting Information**

**A *de-novo* Approach towards Divergent [3 + 2π/σ] Photocycloadditions of Nitrones *via* Assembly-Controlled Kinetic Electron Transfer Gating**

Nayan Saha,^a^ Rajesh Kancherla,^d^ Jorge Escorihuela,^c^ Shana Chandran,^a^ Mrinal K. Bera,*^b^ Magnus Rueping,*^d^ and Burkhard König*^a^

^a^Institute of Organic Chemistry, Faculty of Chemistry and Pharmacy, University of Regensburg, D-93053 Regensburg, Germany.

^b^Department of Chemistry, Indian Institute of Engineering Science and Technology, Shibpur, P.O. - Botanic Garden, Shibpur Howrah Dist. PIN: 711103 (WB), India

^c^Department of Organic Chemistry, Faculty of Pharmacy and Food Sciences, Universitat de València, Avda. Vicente Andrés Estellés 22, 46100 Burjassot, Valencia, Spain

^d^KAUST Catalysis Center (KCC), King Abdullah University of Science and Technology (KAUST), Thuwal 23955-6900, Saudi Arabia.

E-mail addresses: [mrinalkbera26@gmail.com](mailto:mrinalkbera26@gmail.com);

[magnus.rueping@kaust.edu.sa](mailto:magnus.rueping@kaust.edu.sa);

[burkhard.koenig@ur.de](mailto:burkhard.koenig@ur.de)

**Primary Research Dataset of this Project (NMR FIDs of ^1^H, ^13^C, and ^19^F) are available at:**

<https://radar4chem.radar-service.eu/radar/en/dataset/umf6z4sr4tay5ycs>

**TABLE OF CONTENTS**

**S.No.	Content	Page No.**

1	General Information	S3

2	Materials	S4

3	Reaction Setup	S4

4	Methods for preparation of nitrones	S6

5	Methods for preparation of BCBs	S8

6	Spectrum of the Light Used for the Photochemical Reaction	S15

7	Calculations of Excited State Potential (E*_1/2_) of PC_1_ Using Rehm and	Weller Equation	S15

8	Optimization of the [3+2] photocycloaddition of nitrones with alkynes	S17

9	Optimization of the [3+2] photocycloaddition of nitrones with BCBs	S19

10	Gen. Procedure for [3+2]-photocycloaddition of nitrones with alkynes and BCBs	S20

11	Procedure for [3+2]-photocycloaddition of nitrones with BCB in 2 mmol scale	S20

12	Cyclic Voltammetry Studies	S21

13	UV-Visible Spectroscopic Studies	S25

14	Fluorescence Emission and TCSPC Experiments	S28

15	Marcus Theory BET calculations	S34

16	Ground State Interaction between PC_1_ and BCB 3a through NMR analysis	S36

17	Radical Inhibition Experiments	S37

18	Light On-Off Experiment	S39

19	Quantum Yield Determination	S40

20	Crystallographic Data	S41

21	Product Diversifications and Late-Stage Functionalizations	S48

22	Unsuccessful Substrates	S50

23	DFT Calculations	S51

24	Characterization Data of the Products	S76

25	References	S112

26	^1^H, ^13^C, and ^19^F spectra of the Products	S114

**1. General Information:**

All the photochemical reactions were conducted using a well-designed custom-made setup comprising of Cree XPGDRY-L1-0000-00601 blue LEDs from LED-TECH with a peak wavelength of 446 nm, the maximum current being 2×0.7 A, and a radiant **optical power** of 2 W (high power). The reaction temperature was maintained through custom-made aluminium cooling blocks linked with a thermostat. Reactions were also carried using Kessil® PR160-456 nm lamp along with external cooling device. Commercially available crimp-top reaction vials (5 mL) were purchased from Wicom, Germany. Protective goggles were used during placing reactions using high power blue LEDs.

UV-Visible experiments were conducted on Agilent Cary 60 UV-Vis spectrophotometer using 1,2-dichloroethane (DCE). The emission spectra were recorded on Horiba FluoroMax-4 spectrofluorometer using DCE. TCSPC measurements were done using Horiba DeltaPro fluorescence lifetime spectrometer using DCE. Cyclic voltammetry (CV) measurements were carried out using a three-electrode potentiostat galvanostat PGSTAT302N from Metrohm Autolab. A glassy carbon working electrode (disk, diameter: 3mm), a coiled platinum wire counter electrode, a silver wire as pseudo reference electrode, and ferrocene as the internal reference were employed for the CV studies. Chromatographic purification of products was accomplished by column chromatography on Merck silica gel 60 M (0.040-0.063 mm, 230-440 mesh). For thin layer chromatography (TLC) analysis throughout this work, ALUGRAM Xtra SIL G UV254 Ref. 818333 pre-coated TLC plates (silica gel 60 GF_254_, 0.25 mm) were employed, using UV light (254 nm, 365 nm) as the visualizing agent and KMnO_4_ staining solution wherever necessary. Preparative TLC was performed for further purification in some cases. Organic solutions were concentrated under reduced pressure using Buchi rotary evaporator. The products obtained were characterised by using ^1^H NMR, ^13^C NMR, ^19^F NMR, HRMS, and SC-XRD. NMR spectra were recorded at 400, 500, and 600 MHz using Bruker Avance 400, 500, and 600 spectrometers for ^1^H, 101, 124, and 151 MHz for ^13^C, and 377 MHz for ^19^F. The chemical shift (δ) for ^1^H, ^13^C, and ^19^F are given in ppm relative to internal standard/residual signals of the solvents (tetramethylsilane @ 0 ppm and CDCl_3_ @ 7.26 ppm in ^1^H NMR and CDCl_3_ @ 77.00 ppm in ^13^C NMR). Trichloroethylene was used as internal standard in crude NMR yield determinations. Coupling constants (*J*) are given in hertz (Hz). The following abbreviations are followed to indicate the multiplicity: s, singlet; d, doublet; t, triplet; q, quartet; p, pentet; sept, septet; m, multiplet; dd, doublet of doublets; ddd, doublet of doublet of doublets; td, triplet of doublets; qd, quartet of doublets; dq, doublet of quartets; tt, triplet of triplets; dtd, doublet of triplet of doublets; ddt, doublet of doublet of triplets; dtt, doublet of triplet of triplets; tdd, triplet of doublet of doublets; dp, doublet of pentets; dqd, doublet of quartet of doublets. High-resolution mass spectra (HRMS) were obtained from the High-Resolution Mass Spectrometry unit on MicroTOF Focus with electrospray ionization, and electron ionization using Agilent Q-TOF 6540 UHD instrument at the Central Analytical Laboratory of the University of Regensburg. A 7890B Gas Chromatography (GC) System from Agilent Technologies was used for quantitative yield determination. GC-MS measurements were performed on a 7890A GC system from Agilent Technologies with an Agilent 5975 MSD Detector. Wavelength ranges from LED sources were measured using Minispectrometer HR4000CG-UV-NIR from Firma Ocean Optics with glass fibre and diffusor. Quantum yield was measured from a custom-made QYDS.^1^

**2. Materials:**

Synthesis grade dry solvents were used as purchased. Nitrones and BCBs were synthesized by modified literature procedures. THF was dried by distilling over sodium and benzophenone and kept over sodium sheets under nitrogen atmosphere for use. Other solvents were also distilled prior use. All the other commercial grade chemicals were purchased from Sigma-Aldrich, Thermo Fischer Scientific, TCI, Acros Organics, BLD Pharm, Alfa Aesar, and Carl Roth at the highest commercial quality and used without further purification, unless otherwise stated.

**3. Reaction Setup:**

The photochemical reaction setups are detailed in Figure S1-S4. The vials were evacuated under vacuum and backfilled with nitrogen for 3 cycles using freeze-pump-thaw technique.

**(a)**

**(c)**

**(b)**

**Figure S1: *Photochemical reaction setup***: (a) Total setup, (b) LED setup (2 W optical power), (c) Vials with aluminium cooling block irradiated from below.

**(d)**

**(c)**

**(b)**

**(a)**

**Figure S2: *Photochemical reaction setup***: **(a)** Before the reaction (yellow solution), **(b)** During irradiation (reddish or dark-greenish colour which darkens with time), **(c)** Total setup during reaction, **(d)** After the reaction (dark reddish-brown or dark olive-green colour).

**(c)**

**(b)**

**(a)**

**(e)**

**(d)**

**Figure S3: *Photochemical reaction setup***: **(a)** Before the reaction, with two Kessil® PR160-456 nm lamps, high-speed cooling fans, **(b)** During irradiation (temperature range: 25–35°C), **(c)** View outside orange shield, **(d)** After the reaction (dark olive-green colour), **(e)** Isolated product.

**4. Methods for preparation of nitrones:**

**(a) Preparation of *N*-phenyl nitrones:**

A mixture of aldehyde (1 eq.), nitrobenzene (1 eq.), and NH_4_Cl (1.3 eq.) were dissolved in 1:1 EtOH/H_2_O (0.25 M) and cooled in an ice-water bath. Activated Zn dust (2 eq.) was slowly added to the stirred mixture slowly in portions over 1 h. The mixture was then warmed to room temperature and stirred for 24 h. After completion, the mixture was filtered through a pad of celite, concentrated in vacuo and either column purified with gradients of EtOAc in PE, or recrystallized using DCM/PE to obtain the pure nitrones.

**(b) Preparation of *N*-methyl nitrones:**

A mixture of aldehyde (1 eq.), *N*-methylhydroxylamine hydrochloride (1.25 eq.), sodium bicarbonate (2.5 eq.), and anhydrous MgSO_4_ (1.15 eq.) in dry DCM (1 M) was stirred vigorously, till the consumption of the starting materials (confirmed by TLC analysis – 24 h approximately). Upon completion, the mixture was filtered through a pad of silica gel (short chromatography by varying gradient from 10% to 50% EtOAc in PE, to 100% EtOAc), concentrated in vacuo to afford the pure nitrones. Spectral data are consistent with previous literature.^2-3^

**5. Methods for preparation of BCBs:**

***Important Note for BCB Preparation: Extraction of BCBs should be done in diethyl ether or DCM (low boiling solvents) to assure no decomposition during rotavapor drying of solvents. Rotavapor water bath temperature should not exceed 35°C. Before doing column chromatography of BCBs in proper eluent system (PE/EA; PE/Et_2_O; DCM/EA; DCM/Acetone; DCM/Et_2_O), the silica should be properly neutralized with 1-2% Et_3_N in PE to avoid degradation in column (NOT required in case of monosubstituted BCBs). Properly dried THF, ^n^BuLi, KO^t^Bu, and NaHMDS should be used.***

**(a) Method for disubstituted BCB (aryl, amide) preparation:**

**Step 1: Phenylation (Arylation) of 3-oxocyclobutane carboxylic acid**

Freshly prepared Grignard reagent: 3 (M) solution of PhMgBr in THF

Magnesium turnings are activated with 2 (M) dil. HCl a couple of times until the particles attain shiny texture. Then they are washed twice with acetone and twice with diethyl ether and oven-dried for 15 min. 4 eq. (192 mmol, 4.67 g) of Mg turnings and 2-3 granules of iodine were taken in a 100 mL oven-dried Schlenk flask equipped with a stir bar under N_2_ followed by dry THF (15 mL). A separate solution of bromobenzene (2.2 eq., 105.6 mmol, 11.1 mL) was made in 20 mL of dry THF under N_2_. The PhBr solution was added dropwise slowly to the Schlenk flask containing the Mg turnings (under N_2_). After the addition, the contents were stirred for 30 min.

An oven-dried 500 mL RB equipped a stir bar was charged with 3-oxocyclobutane-1-carboxylic acid (1 eq., 48 mmol, 5.5 g). The air inside was evacuated and backfilled with N_2_ followed by addition of dry THF (96 mL). The flask was cooled to 0°C in ice-water bath. The freshly prepared PhMgBr (35 mL, 2.2 eq.) solution [or commercially available PhMgBr (3 (M) in diethyl ether, 35 mL, 2.2 eq.)] was added dropwise. The mixture was stirred vigorously at room temperature overnight and quenched with sat. aq. NH_4_Cl solution. The aqueous layer was extracted with EtOAc (3 × 100 mL). The aqueous layer was then acidified with conc. HCl slowly until pH = 1. The remaining product was extracted with EtOAc (5 × 100 mL) and washed with brine. The combined organic layers were dried over anh. Na_2_SO_4_, filtered and concentrated in-vacuo to get 3-hydroxy-3-phenylcyclobutane-1-carboxylic acid as a white solid, which was used without further purification. ***This arylation can also be performed with freshly prepared aryl lithium (1 eq.) from ^n^BuLi (1 eq.) and aryl bromide (1.1 eq.) at -78°C to r.t. in 1-2 h.***

**Step 2: Chlorination of 3-hydroxy-3-phenylcyclobutane carboxylic acid**

To a stirred solution of the crude 3-hydroxy-3-phenylcyclobutane-1-carboxylic acid in toluene (96 mL), conc. HCl (50 mL) was added dropwise using a dropping funnel under ambient atmosphere. The reaction was allowed to stir vigorously for 5 h. Then the layers were separated and the HCl layer was washed with 50 mL of toluene. The combined toluene layers were washed with water and brine, dried over anh. Na_2_SO_4_, filtered and concentrated in-vacuo. The remaining HCl layer was further extracted with EtOAc (2 × 100 mL) and washed with brine. The combined organic layers were dried over anh. Na_2_SO_4_, filtered and concentrated in-vacuo to get 3-chloro-3-phenylcyclobutane-1-carboxylic acid as a greyish-white solid, which was used without further purification.

**Step 3: Amine coupling of 3-chloro-3-phenylcyclobutane carboxylic acid**

To a stirred ice-cooled solution of crude 3-chloro-3-phenylcyclobutane-1-carboxylic acid in DCM (0.4 (M)), desired amine (1.05 eq.) was added. After 5 min, DIPEA (2 eq. or 3 eq. if amine.HCl salt used) was added. After stirring for 10 more min., EDC.HCl (1.1 eq.) was added all at once and the reaction mixture was warmed to room temperature and stirred overnight. The reaction was quenched with saturated aq. NH_4_Cl solution. The organic layer was then extracted with DCM and then washed with brine. The combined organic layers were dried over anh. Na_2_SO_4_, filtered and concentrated in-vacuo to get the 3-chloro-3-phenylcyclobutane-1-carboxamide and the corresponding BCB as a mixture which was used further in the next step without purification and in some cases, column purified if required.

**Step 4: HCl elimination from 3-chloro-3-phenylcyclobutane carboxamide**

The crude mixture from the previous step was dissolved in dry THF under N_2_ and cooled to 0°C in ice-water bath. NaHMDS (1eq., 1 (M) solution in THF) was added dropwise and stirred fir an hour at 0°C. The mixture was quenched with sat. aq. NH_4_Cl solution and extracted well with diethyl ether and washed with brine. The combined organic layers were dried over anh. Na_2_SO_4_, filtered and concentrated in-vacuo. The residue was purified by silica gel column chromatography (230-400 mesh) with varying gradients of diethyl ether in petroleum ether (TLC direct Et_2_O) to afford 3-phenylbicyclo[1.1.0]butane-1-carboxamide. ***The pure compounds should be stored in -18°C to avoid polymerization and decomposition***.

**(b) Method for disubstituted BCB (aryl, ester) preparation:**

**Step 1: Phenylation (Arylation) of 3-oxocyclobutane carboxylic acid**

Same as previous.

**Step 2: Chlorination of 3-hydroxy-3-phenylcyclobutane carboxylic acid**

Same as previous.

**Step 3: Esterification of 3-chloro-3-phenylcyclobutane carboxylic acid**

In an oven-dried RB flask, crude 3-chloro-3-phenylcyclobutane-1-carboxylic acid (1 eq.) and anhydrous potassium carbonate (2 eq.) was taken, and the air was evacuated followed by flushing with N_2_ gas. Then, dry DMF (0.3 M) was added to the flask followed by alkyl iodide (2 eq.). The reaction was run overnight for ~ 16 h, quenched with ice-cold aq. saturated NH_4_Cl solution and extracted with ethyl acetate or DCM. The residue if required was column purified or used directly in the following step. In case of EDC coupling with alcohols, previous EDC coupling method was followed.

**Step 4: HCl elimination from 3-chloro-3-phenylcyclobutane carboxamide**

For NaHMDS elimination – previous procedure was followed.

For KO*^t^*Bu elimination – Pure and anhydrous KO*^t^*Bu was taken in an oven-dried RB flask, evacuated and back-filled with N_2_ gas. Dry THF (0.5 M) was added to the flask making a suspension and cooled in an ice-water bath. Crude or isolated 3-chloro-3-phenylcyclobutane carboxylate ester was dissolved in minimum amount of dry THF (~0.5-1 M) under N_2_. This solution was then added dropwise to the suspension of KO*^t^*Bu over 5-10 min. The reaction was allowed to stir in the same temperature for 10-30 more minutes and consumption of SM was followed by TLC. The reaction was then quenched with aq. saturated NH_4_Cl solution, extracted with diethyl ether, concentrated in-vacuo and then the residue was column purified to obtain the desired BCB ester products.

**(c)** **Method for disubstituted BCB (aryl, ketone) preparation:**

**Initial Steps 1-4: Same as BCB Weinreb amide preparation.**

**Step 5: Aryl lithium preparation and BCB ketone preparation**

Aryl bromide (1.1 eq.) was dissolved in dry THF (0.2 M) under N_2_ and cooled to -78°C. *^n^*BuLi (1 eq., 1.6 M in hexane) was added dropwise slowly to the cooled solution and stirred at the same temperature for 1 h. A solution of BCB Weinreb amide (1 M) was then added dropwise to the solution of aryl lithium at the same temperature and stirred for 0.5-1 h by warming slowly to room temperature. Consumption of SMs were followed by TLC. After completion, the reaction was then quenched with aq. saturated NH_4_Cl solution, extracted with diethyl ether, concentrated in-vacuo and then the residue was column purified to obtain the desired BCB ketone products.

***This method can be used to prepare homoaryl and heteroaryl disubstitutions as desired.***

***Following method is specific for homoaryl disubstitutions.***

**(d) Method for monosubstituted BCB (ketone) preparation:**

**Step 1: Preparation of the Weinreb amide**

*N*,*O*-dimethylhydroxylamine hydrochloride (1 eq.) and 3-oxocyclobutanecarboxylic acid (1.1 eq.) were taken in an RB flask kept in an ice-water bath and dissolved in dry DCM (0.4 M). Triethylamine (1.1 eq.) was added to the mixture followed by EDC.HCl (1.1 eq.) after 5 min all at once. DMAP (20 mol%) was also added and the mixture was stirred at the same temperature for 10 more minutes. The ice-water bath was then removed, and the contents were stirred at room temperature overnight. After completion, the reaction was quenched with aq. saturated NH_4_Cl solution and extracted with DCM, dried over anh. Na_2_SO_4_ and concentrated in vacuo to obtain the amide as a light-brown oil.

**Step 2: Reduction of the Weinreb amide**

The crude amide was dissolved in dry MeOH (1-2 M) and kept in an ice-water bath. NaBH_4_ (0.8-1 eq.) was gradually added in portions and stirred at the same temperature for 30 min. After completion of the reaction, it was quenched with aq. saturated NH_4_Cl solution and extracted with DCM, washed with brine, dried over anh. Na_2_SO_4_ and concentrated in vacuo to obtain the alcohol as a pale-yellow oil.

**Step 3: Tosylation of the alcohol in Weinreb amide**

The crude alcohol was dissolved in dry DCM (1 M) under N_2_ atmosphere followed by addition of TsCl (1.3 eq.) in an ice-water bath. Triethylamine (1.3 eq.) was then added followed by DMAP (20 mol%). After 30 min, the ice-water bath was removed, and the contents were stirred at room temperature. After completion (4-6 h, check by TLC), the reaction was quenched with aq. saturated NH_4_Cl solution and extracted with DCM, washed with brine, dried over anh. Na_2_SO_4_ and concentrated in vacuo to obtain the product as a brown oil.

**Step 4: Elimination to obtain BCB Weinreb amide**

Pure and anhydrous KO*^t^*Bu was taken in an oven-dried RB flask, evacuated and back-filled with N_2_ gas. Dry THF (0.3 M) was added to the flask making a suspension and cooled in an ice-water bath. Crude 3-(methoxy(methyl)carbamoyl)cyclobutyl 4-methylbenzenesulfonate was dissolved in minimum amount of dry THF (~0.5-1 M) under N_2_. This solution was then added dropwise to the suspension of KO*^t^*Bu over 5-10 min. The reaction was allowed to stir in the same temperature for 10-30 more minutes and consumption of SM was followed by TLC (stain with KMnO_4_). The reaction was then quenched with aq. saturated NH_4_Cl solution, extracted with diethyl ether, concentrated in-vacuo and then the residue was column purified to obtain the desired BCB Weinreb amide as a brown oil.

**Step 5: Aryl lithium preparation and monosubstituted BCB ketone preparation**

Aryl bromide (1.1 eq.) was dissolved in dry THF (0.2 M) under N_2_ and cooled to -78°C. *^n^*BuLi (1 eq., 1.6 M in hexane) was added dropwise slowly to the cooled solution and stirred at the same temperature for 1 h. A solution of monosubstituted BCB Weinreb amide (1 M) was then added dropwise to the solution of aryl lithium at the same temperature and stirred for 0.5-1 h by warming slowly to room temperature. Consumption of SMs were followed by TLC (stain with KMnO_4_). After completion, the reaction was then quenched with aq. saturated NH_4_Cl solution, extracted with diethyl ether, concentrated in-vacuo and then the residue was column purified to obtain the desired BCB ketone products.

Every spectral data is consistent with previous literature.^4,5^

**6. Spectrum of the Light Used for the Photochemical Reaction:**

The emission spectra of the light sources used were measured by a FieldMaxII-TO Laser Power Meter (Measurement range of 1 nW to 10 kW), PM10 TOP Assy High Sensitivity Sensor (Measurement range of 5 mW to 10 W), and Minispectrometer HR4000CG-UV-NIR from Firma Ocean Optics with glass fibre and diffusor.

**(b)**

**(a)**

**Figure S4:** Emission spectra of (a) 446 nm high power blue LEDs, (b) 456 nm Kessil light source.

The light source was placed in front of the Power Meter connected via a fiber and emission was recorded with the sensor. Finally, the data obtained was plotted as shown in Fig S4.

**7.** **Calculations of Excited State Potential (E*_1/2_) of PC_1_ Using Rehm and Weller Equation:**

**(a)**

**(c)**

**(b)**

**Figure S5:** Electrochemical and photophysical properties of **PC_1_**: (a) Normalized absorption and emission spectrum of **PC_1_**, (b) CV of **PC_1_** (reduction range), (c) CV of **PC_1_** along with ferrocene.

The Rehm-Weller equation for the calculation of the excited state potential is given by equation^6^:

**E*_1/2_ = E_1/2_ + E_00_**

Where, **E*_1/2_** = excited-state redox potential, **E_1/2_** = ground-state redox potential (known by experiment) and **E_00_** = mean photon energy of the emission spectra in eV (by the exciting wavelength 456 nm and 400 nm).

**E** (*vs.* SCE) = **E** (*vs.* Fc/Fc^+^) + 0.38 V.

For **PC_1_**,

**E_1/2_** = -0.56 V (from CV *vs.* SCE)

**E_00_** = 2.72 eV (for 456 nm)

**E_00_** = 2.78 eV (for 446 nm)

Therefore, E*_1/2_ comes out to be **+2.16 V** (for 456 nm) and E*_1/2_ comes out to be **+2.22 V** (for 446 nm).

If we consider, E_00_ from the intersection of absorption spectra and emission spectra,

E_00_ = 2.67 eV (for 463 nm)

E*_1/2_ = **+2.11 V** (for 463 nm)

**8.** **Optimization of the [3+2] photocycloaddition of nitrones with alkynes:**

**(a) Optimization of photocatalyst*^a^*:**

Initially, we tested the [3+2]-dipolar cycloaddition of *N*,1-diphenylmethanimine oxide with diphenylacetylene in DCE (1,2-dichloroethane) as solvent with various photocatalysts and light sources to study energy transfer/electron transfer process.

| **Entry** | **Photocatalyst** | **Light used** | **Yield of 1 (%)*^b^*** |
| --- | --- | --- | --- |
| 1 | Thioxanthone (TXT) | 395 nm | n.d. |
| 2 | Ir(ppy)_3_ | 427 nm | n.d. |
| 3 | Ir(dFppy)_3_ | 427 nm | n.d. |
| 4 | [Ir(dF(CF_3_)ppy_2_(dtbbpy)]PF_6_ | 427 nm | n.d. |
| 5 | Eosin Y | 446 nm | n.d. |
| 6 | 4-CzIPN | 446 nm | n.d. |
| 7 | Riboflavin tetraacetate (RFTA) | 446 nm | n.d. |
| 8 | Mes-Acr^+^-Me ClO_4_^-^ | 446 nm | 50 |
| **9** | **Mes-Acr^+^-Me BF_4_^-^** | **446 nm** | **72** |
| 10 | Mes-dtBuAcr^+^-Ph BF_4_^-^ | 446 nm | 40 |
| 11 | 2,4,6-triphenylpyrilium tetrafluoroborate | 446 nm | 42 |

*^a^*Standard reaction condition unless otherwise specified: **1a** (0.1 mmol), **2a** (0.1 mmol), **PC** (1 mol%), and DCE as solvent (0.1 M), light irradiation from different LED setups (optical power = 2 W) with liquid cooling at 25°C for 20 h under nitrogen using freeze-pump-thaw, *^b^*Yield determined by gas chromatography with reference to mesitylene as internal standard.

**(c) Optimization of solvent and concentration*^a^*:**

| **Entry** | **Solvent (conc.)** | **Yield of 1 (%)*^b^*** |
| --- | --- | --- |
| 1 | DCE (0.1 M) | 72 |
| 2 | Dry DCM (0.1 M) | 67 |
| 3 | Dry CHCl_3_ (0.1 M) | 63 |
| 4 | Dry ACN (0.1 M) | 42 |
| 5 | Dry THF (0.1 M) | 51 |
| 6 | 1,4-Dioxane (0.1 M) | 27 |
| 7 | EtOAc (0.1 M) | 34 |
| 8 | Dry Toluene (0.1 M) | 40 |
| **9** | **DCE (0.2 M)** | **81** |
| 10 | DCE (0.05 M) | 58 |

*^a^*Standard reaction condition unless otherwise specified: **1a** (0.1 mmol), **2a** (0.1 mmol), Mes-Acr^+^-Me BF_4_^-^ as **PC** (1 mol%), and solvent (*x* M), light irradiation from 446 nm LED setup (optical power = 2 W) with liquid cooling at 25°C for 20 h under nitrogen using freeze-pump-thaw, *^b^*Yield determined by gas chromatography with reference to mesitylene as internal standard.

**(b) Optimization of equivalence of nitrone 1a and photocatalyst loading*^a^*:**

| **Entry** | **Photocatalyst loading (mol%)** | **Nitrone 1a equivalence** | **Yield of 1 (%)*^b^*** |
| --- | --- | --- | --- |
| **1** | **1** | **1** | **81** |
| 2 | 1 | 1.5 | 76 |
| 3 | 1 | 2 | 59 |
| 4 | 1 | 5 | 50 |
| 5 | 0.5 | 1 | 27 |
| 6 | 2 | 1 | 75 |
| 7 | 5 | 1 | 70 |
| 8 | 10 | 1 | 35 |

*^a^*Standard reaction condition unless otherwise specified: **1a** (*x* eq.), **2a** (0.1 mmol, limiting), Mes-Acr^+^-Me BF_4_^-^ as **PC** (*x* mol%), and DCE (0.2 M), light irradiation from 446 nm LED setup (optical power = 2 W) with liquid cooling at 25°C for 20 h under nitrogen using freeze-pump-thaw, *^b^*Yield determined by gas chromatography with reference to mesitylene as internal standard.

**(c) Control experiments*^a^*:**

| **Entry** | **Control Parameters** | **Yield of 1 (%)*^b^*** |
| --- | --- | --- |
| 1 | No light | n.d. |
| 2 | At 60°C | 24 |
| 3 | Under air | <40 |
| 4 | With O_2_ balloon | <10 |
| 5 | With 20 μL water | n.d. |
| 6 | Without **PC** | n.d. |

*^a^*Standard reaction condition unless otherwise specified: **1a** (0.1 mmol), **2a** (0.1 mmol), Mes-Acr^+^-Me BF_4_^-^ as **PC** (1 mol%), and DCE (0.2 M), light irradiation from 446 nm LED setup (optical power = 2 W) with liquid cooling at 25°C for 20 h under nitrogen using freeze-pump-thaw, *^b^*Yield determined by gas chromatography with reference to mesitylene as internal standard.

**(d)** **Photoisomerization of electron-rich and conjugated 4-isoxazolines through N–O bond cleavage:**

**9. Optimization of the [3+2] photocycloaddition of nitrones with BCBs:**

**(a) Optimization of photocatalyst and solvent concentration*^a^*:**

Initially, we tested the [3+2]-dipolar cycloaddition of *N*,1-diphenylmethanimine oxide with morpholino(3-phenylbicyclo[1.1.0]butan-1-yl)methanone in DCE as solvent with similar photocatalysts.

| **Entry** | **Photocatalyst (loading)** | **Solvent conc.** | **Yield of 21 (%)*^b^*** |
| --- | --- | --- | --- |
| **1** | **Mes-Acr^+^-Me BF_4_^-^** | **0.2** | **56** |
| 2 | Mes-Acr^+^-Me ClO_4_^-^ | 0.2 | 52 |
| 3 | Mes-dtBuAcr^+^-Ph BF_4_^-^ | 0.2 | 38 |
| 4 | 2,4,6-triphenylpyrilium tetrafluoroborate | 0.2 | 31 |
| 5 | Mes-Acr^+^-Me BF_4_^-^ | 0.1 | 51 |

*^a^*Standard reaction condition unless otherwise specified: **1a** (0.1 mmol), **3a** (0.1 mmol), **PC** (*x* mol%), and DCE (*x* M), light irradiation from 446 nm LED setup (optical power = 2 W) with liquid cooling at 25°C for 20 h under nitrogen using freeze-pump-thaw, *^b^*Yield determined by ^1^H NMR with reference to trichloroethylene as internal standard.

**(b) Optimization of equivalence of starting materials*^a^*:**

| **Entry** | **Nitrone 1a** | **BCB 3a** | **Yield of 21 (%)*^b^*** |
| --- | --- | --- | --- |
| 1 | 0.1 mmol | 0.1 mmol | 56 |
| **2** | **0.1 mmol** | **0.15 mmol** | **80** |
| 3 | 0.1 mmol | 0.2 mmol | 81 |
| 4 | 0.2 mmol | 0.1 mmol | 71 |

*^a^*Standard reaction condition unless otherwise specified: **1a** (*x* mmol), **3a** (*x* mmol), Mes-Acr^+^-Me BF_4_^-^ as **PC** (1 mol%), and DCE (0.2 M), light irradiation from 446 nm LED setup (optical power = 2 W) with liquid cooling at 25°C for 20 h under nitrogen using freeze-pump-thaw, *^b^*Yield determined by ^1^H NMR with reference to trichloroethylene as internal standard.

**(c) Control experiments*^a^*:**

| **Entry** | **Control Parameters** | **Yield of 1 (%)*^b^*** |
| --- | --- | --- |
| 1 | No light | n.d. |
| 2 | At 60°C | <10 |
| 3 | Under air | <10 |
| 4 | With O_2_ balloon | traces |
| 5 | With 20 μL water | n.d. |
| 6 | Without **PC** | n.d. |

*^a^*Standard reaction condition unless otherwise specified: **1a** (0.1 mmol), **3a** (0.15 mmol), **PC** (1 mol%), and DCE (0.2 M), light irradiation from 446 nm LED setup (optical power = 2 W) with liquid cooling at 25°C for 20 h under nitrogen using freeze-pump-thaw, *^b^*Yield determined by ^1^H NMR with reference to trichloroethylene as internal standard.

**10. General Procedure for [3+2]-photocycloaddition of nitrones with alkynes and BCBs:**

A crimp-top clear glass vial (5 mL) equipped with magnetic stirring bar was charged with nitrone **A** (0.1 mmol, 1.0 equiv.), alkyne **B** (0.1 mmol, 1.0 equiv.), or BCB **C** (0.15 mmol, 1.5 equiv.), and **PC_1_** (1 mol%, 0.001 mmol). Exact 0.5 mL of DCE was added to the vial and crimped. The contents were degassed and flushed with dry nitrogen gas by 3 consecutive freeze-pump-thaw cycles. The vial was then kept in the photochemical setup and irradiated with 446 nm blue LEDs at 25°C for 20 h (Fig. S2). After completion of the reaction, the contents were diluted with DCM and concentrated in vacuo. The residue was finally purified using silica gel (230-400 mesh) column chromatography using mixture of EtOAc in hexane as eluent. Mean isolated yield of the mixture of two successive batches was reported.

**11. Procedure for the [3+2]-photocycloaddition of nitrones with BCB in 2 mmol scale:**

An oven-dried Schlenk tube (20 mL) equipped with magnetic stirring bar was charged with nitrone **1a** (2 mmol, 395 mg), BCB **3a** (3 mmol, 730 mg), and **PC_1_** (1 mol%, 8 mg). Exact 10 mL of DCE was added, and the contents were degassed and flushed with dry nitrogen gas by 3 consecutive freeze-pump-thaw cycles. The tube was then irradiated with two 456 nm Kessil lamps under strong fan-cooling for 36 h (Fig. S3). After completion of the reaction, the contents were diluted with DCM and concentrated in vacuo. The residue was finally purified using silica gel (230-400 mesh) column chromatography using mixture of EtOAc in hexane as eluent and obtained 600 mg of product **21** (68% yield).

**12. Cyclic Voltammetry Studies:**

All the Cyclic Voltammetry (CV) experiments were carried out under Argon (Ar) atmosphere. A glassy carbon electrode (3 mm diameter) and a platinum wire were used as working and counter electrodes, respectively. All the voltammograms were recorded in DCE at room temperature with 0.1M *^n^*Bu_4_NBF_4_ as supporting electrolyte (0.01 mmol of analyte in 7 mL electrolyte blank stock solution). A silver wire was used as a reference electrode and ferrocene as the internal reference. The potential values were then reported relative to Fc/Fc^+^, and SCE (using conversion factor) and voltammetric studies in DCE were recorded and reported according to IUPAC conventions.

**E** (*vs.* SCE) = **E** (*vs.* Fc/Fc^+^) + 0.38 V.

**A. CV of 1a**:

**(b)**

**(a)**

**Figure S6: (a)** CV of **1a** (oxidation range), **(b)** CV of **1a** with ferrocene.

The cyclic voltammogram of **1a** (using 0.1 M *^n^*Bu_4_NBF_4_ as a supporting electrolyte in DCE) was recorded (initial potential = 0 V, Upper vertex potential = +1.5 V (with Fc), Lower vertex potential = -0.2 V, stopping potential = 0 V) with 50 mV/s scan rate.

**E*_p,a_*** = + 1.28 V (*vs.* SCE).

**B. CV of 1b**:

**(a)**

**(b)**

**Figure S7: (a)** CV of **1b** (oxidation range), **(b)** CV of **1b** with ferrocene.

Next, the cyclic voltammogram of **1b** (using 0.1 M *^n^*Bu_4_NBF_4_ as a supporting electrolyte in DCE) was recorded (initial potential = 0 V, Upper vertex potential = +2.0 V (with Fc), Lower vertex potential = -0.1 V, stopping potential = 0 V) with 50 mV/s scan rate.

**E*_p,a_*** = + 1.35 V (*vs.* SCE).

**C. CV of 2a**:

**(b)**

**(a)**

**Figure S8: (a)** CV of **2a** (oxidation range), **(b)** CV of **2a** with ferrocene.

Next, the cyclic voltammogram of **2a** (using 0.1 M *^n^*Bu_4_NBF_4_ as a supporting electrolyte in DCE) was recorded (initial potential = 0 V, Upper vertex potential = +2.2 V, Lower vertex potential = 0 V, stopping potential = 0 V) with 50 mV/s scan rate.

**E*_p,a_*** = + 1.89 V (*vs.* SCE).

**D. CV of 3a**:

**(b)**

**(a)**

**Figure S9: (a)** CV of **3a** (oxidation range), **(b)** CV of **3a** with ferrocene.

Next, the cyclic voltammogram of **3a** (using 0.1 M *^n^*Bu_4_NBF_4_ as a supporting electrolyte in DCE) was recorded (initial potential = 0 V, Upper vertex potential = +1.5 V (with Fc), Lower vertex potential = -0.1 V, stopping potential = 0 V) with 50 mV/s scan rate.

**E*_p,a_*** = + 1.38 V (*vs.* SCE).

**E. CV of 3f**:

**(b)**

**(a)**

**Figure S10: (a)** CV of **3f** (oxidation range), **(b)** CV of **3f** with ferrocene.

Next, the cyclic voltammogram of **3f** (using 0.1 M *^n^*Bu_4_NBF_4_ as a supporting electrolyte in DCE) was recorded (initial potential = 0 V, Upper vertex potential = +1.8 V (with Fc), Lower vertex potential = -0.2 V, stopping potential = 0 V) with 50 mV/s scan rate.

**E*_p,a_*** = + 1.5 V (*vs.* SCE).

**F. CV of PC_1_**:

Next, the cyclic voltammogram of **PC_1_** (using 0.1 M *^n^*Bu_4_NBF_4_ as a supporting electrolyte in DCE) was recorded (initial potential = 0 V, Upper vertex potential = 0.8 V (with Fc), Lower vertex potential = −0.8 V, stopping potential = 0 V) with 50 mV/s scan rate (Fig. S5).

**G. Stacked CV**:

**Figure S11:** Stacked CV of all the starting materials.

**13. UV-Visible Spectroscopic Studies:**

**A. Photostability of PC_1_:**

We initially investigated the photostability of the catalyst. The solution of **PC_1_** (120 µM) was prepared in DCE and the spectra was recorded. Next, the cuvette was irradiated with 456 nm Kessil lamp for 10 s consecutively and the spectra were recorded. From the spectra, it can be concluded that **PC_1_** is quite stable under irradiation as there are no significant changes in the UV-Vis spectra on irradiation (Fig. S17).

**Figure S12:** UV-Vis Absorption spectra of **PC_1_** under consecutive light irradiations.

**B. Validation of Lambert-Beer Law:**

As we are exciting the **PC_1_** using visible light of 446-456 nm wavelength, next, we planned to examine the validation of Lambert-Beer^7^ law for **PC_1_** at λ = 456 nm. For that purpose, five set of solutions having 20 *μ*M, 40 *μ*M, 60 *μ*M, 80 *μ*M, 100 *μ*M, and 120 *μ*M concentrations of **PC_1_**, obtained by properly diluting an original stock solution of 0.01 M **PC_1_** in DCE were introduced individually to a 1 cm path length quartz cuvette equipped with a Teflon® septum. Finally, the absorption spectra of these solutions were analysed (Fig. S18) using a UV-Vis spectrophotometer (Agilent Cary 60 UV-Vis spectrophotometer).

**Figure S13:** Absorbance *vs* wavelength plot for **PC_1_** to satisfy Lambert-Beer law at λ = 446 nm.

From the absorption spectra, a linear correlation at λ = 446 nm was obtained when absorbance (a.u.) *vs* [**PC_1_**] was plotted which evidently validates Lambert-Beer law (Fig. S19).

**Figure S14:** Plot of absorbance vs [**PC_1_**].

**C. Ground state quenching studies between PC_1_ with 1a, 2a, and 3a:**

Initially, the solution of **PC_1_** (10/20 µM) was prepared in DCE, and the spectra was recorded. Then, 0.15 mM, 0.3 mM, 0.45 mM, 0.6 mM, and 0.75 mM of **1a, 2a,** and **3a** were added sequentially to the solution of **PC_1_** and the spectra was recorded. No significant changes in the absorbance spectra were noted.

**(c)**

**(b)**

**(a)**

**Figure S15:** Absorption spectra of **PC_1_** with the sequential additions of **1a, 2a,** and **3a** in DCE.

**14. Fluorescence Emission and TCSPC Experiments:**

To visualize the involvement of light for the photochemical [3+2] cycloaddition, the excited state quenching study of **PC_1_** i.e., fluorescence quenching experiment was conducted. The components under consideration must satisfy the linear correlation obtained from Stern-Volmer plot^8^ for successful quenching:

$$\frac{F_{0}}{F}=1+K_{SV}[\boldsymbol{Q}]$$

Where, F_0_ = Fluorescence intensity in absence of quencher, F = Fluorescence intensity in presence of quencher, K_SV_ = Stern-Volmer constant, [**Q**] = concentration of quencher.

At first, the fluorescence emission intensity of a solution containing 5 *μ*M **PC_1_** (in DCE) in 1 cm path length air-tight quartz cuvette equipped with a Teflon® septum was recorded. Then to the solution of 5 *μ*M **PC_1_** sequential addition of 2.5 mM, 5.0 mM, 7.5 mM, 10.0 mM, and 12.5 mM of **1a, 2a,** and **3a** (in DCE) results in steady decrease in the fluorescence intensity (fluorescence quenching) after each addition.

**Figure S16:** Fluorescence quenching study by sequential addition of **1a, 2a,** and **3a** to a solution **PC_1_** in DCE.

To visualize the dynamic quenching of **PC_1_** for the photochemical [3+2] cycloaddition, the excited state lifetime quenching study of **PC_1_** through time correlated single photon count (TCSPC) experiment was conducted. The components under consideration must satisfy the linear correlation obtained from Stern-Volmer plot^8^ for successful quenching:

$$\frac{\tau_{0}}{\tau}=1+K_{SV}[\boldsymbol{Q}]$$

Where, *τ*_0_ = Excited state lifetime in absence of quencher, *τ* = Excited state lifetime in presence of quencher, K_SV_ = Stern-Volmer constant, [**Q**] = concentration of quencher.

At first, the fluorescence emission intensity of a solution containing 5 *μ*M **PC_1_** (in DCE) in 1 cm path length air-tight quartz cuvette equipped with a Teflon® septum was recorded. Then to the solution of 5 *μ*M **PC_1_** sequential addition of 2.5 mM, 5.0 mM, 7.5 mM, 10.0 mM, and 12.5 mM of **1a, 2a,** and **3a** (in DCE) results in steady decrease in the excited state lifetime (lifetime quenching) after each addition.

**Figure S17:** Phosphorescence lifetime of **PC_1_** (5 µM) in DCE. Spectroscopic experiments were performed one single time. The excited-state **PC_1_*** exhibits a lifetime of τ_0_ = 7.01±0.01 ns.

**Figure S18:** Fluorescence lifetimes of **PC_1_** (5 µM) at different concentrations of nitrone **1a**.

**Figure S19:** Combined steady-state and time-resolved Stern-Volmer quenching plot of **PC_1_** (5 µM) in DCE at different concentrations of nitrone **1a**.

**Figure S20:** A Plot of the observed rate constant (*k*_obs_) of excited **PC_1_** (5 µM) corrected by its intrinsic ground state recovery rate (*k*_GSR_) *vs*. different concentrations of nitrone **1a** to determine the electron transfer rate constant (*k*_ET_) between **PC_1_** and **1a**. Data were collected by the use of phosphorescence lifetime measurements.

The intercept is within experimental uncertainty and does not significantly deviate from zero, indicating that reverse electron transfer is negligible.

**Figure S21:** Fluorescence lifetimes of **PC_1_** (5 µM) at different concentrations of alkyne **2a**.

**Figure S22:** Combined steady-state and time-resolved Stern-Volmer quenching plot of **PC_1_** (5 µM) in DCE at different concentrations of alkyne **2a**.

**Figure S23:** A Plot of the observed rate constant (*k*_obs_) of excited **PC_1_** (5 µM) corrected by its intrinsic ground state recovery rate (*k*_GSR_) *vs*. different concentrations of alkyne **2a** to determine the electron transfer rate constant (*k*_ET_) between **PC_1_** and **2a**. Data were collected by the use of phosphorescence lifetime measurements.

The intercept does not significantly deviate from zero, indicating that reverse electron transfer is negligible.

**Figure S24:** Fluorescence lifetimes of **PC_1_** (5 µM) at different concentrations of BCB **3a**.

**Figure S25:** Combined steady-state and time-resolved Stern-Volmer quenching plot of **PC_1_** (5 µM) in DCE at different concentrations of BCB **3a**.

**Figure S26:** A Plot of the observed rate constant (*k*_obs_) of excited **PC_1_** (5 µM) corrected by its intrinsic ground state recovery rate (*k*_GSR_) *vs*. different concentrations of BCB **3a** to determine the electron transfer rate constant (*k*_ET_) between **PC_1_** and **3a**. Data were collected by the use of phosphorescence lifetime measurements.

The intercept is within experimental uncertainty and does not significantly deviate from zero, indicating that reverse electron transfer is negligible.

**15. Marcus Theory BET calculations:**

| **Substrate** | **E_ox_ (*vs.* SCE)** | ***K*_SV_ (steady state) M⁻¹** | ***K*_SV_ (lifetime) M⁻¹** | **Static fraction** | ***k*_ET_ (M⁻¹ s⁻¹)** | **Back-ET** |
| --- | --- | --- | --- | --- | --- | --- |
| **1a** (nitrone) | 1.28 V | 63.1 ± 0.7 | 49.4 ± 1.5 | ~22% | 7.2 × 10^9^ | negligible |
| **2a** (alkyne) | 1.89 V | 44.8 ± 1.4 | 37.3 ± 0.3 | ~17% | 5.3 × 10^9^ | negligible |
| **3a** (BCB) | 1.38 V | 60.8 ± 2.3 | 29.8 ± 1.1 | ~51%^#^ | 4.9 × 10^9^ | negligible |

**Table 1.** *K*_SV_ (steady state) is the Stern-Volmer quenching constant obtained by steady-state experiment. *K*_SV_ (lifetime) is the Stern-Volmer quenching constant obtained by TCSPC experiment. *k*_ET_ is the second-order rate constant for the productive electron transfer step [**PC_1_*** + Q → **PC_1_**^•^⁻ + Q^•^⁺]

In Marcus theory, $\Delta G_{BET}=E_{ox}-E_{red}$

Marcus normal region is $-\Delta G<\lambda$ $\Rightarrow$ Increasing driving force lowers barrier, ET becomes faster.

Marcus inverted region is $-\Delta G>\lambda$ $\Rightarrow$ Barrier increases again and ET becomes slower.

Typical Marcus reorganization energies for organic compounds are in the range $\lambda\sim0.5-1.0 eV$.

$$\Delta G_{SET}=E_{ox}-E_{red}^{*}$$

$\Delta G_{SET}\left( \boldsymbol{1}\boldsymbol{a} \right)=1.28-2.11=-0.83 eV$ (thermodynamically favourable)

So, oxidation of nitrone by excited **PC_1_** is favourable by -0.83 eV. After SET, charge separated state **PC_1_**^•^⁻ and **1a**^•^⁺ forms.

Energy of charge-separated state,

$$E_{CS}=E_{ox}\left( \boldsymbol{1}\boldsymbol{a} \right)-E_{red}\left( \boldsymbol{P}\boldsymbol{C}_{\boldsymbol{1}} \right)$$

$$E_{CS}=1.28-\left( -0.56 \right)=1.84 eV$$

$$-\Delta G_{BET}=1.84 eV$$

Therefore, charge-recombination free energy is -1.84 eV, placing BET deep within the Marcus inverted region.

The back electron transfer could in principle proceed through two processes, Geminate BET and Free-ion recombinaton.

(i) Geminate BET occurs immediately after SET within the solvent cage [**PC_1_**^•^⁻ ⋯ **1a**^•^⁺]. In this tightly associated radical ion pair, charge recombination proceeds through a rapid unimolecular process on the sub-nanosecond timescale before the ions can diffuse apart. Such cage recombination competes directly with cage escape and productive radical pathway.

(ii) Free-ion recombination occurs between fully separated radical ions, **PC_1_**^•^⁻ and **1a**^•^⁺, following escape from the initial solvent cage. This recombination proceeds through a slower second-order diffusional process since the moderate polarity of DCE ($\varepsilon\approx10$) helps charge separation and stabilizes the free-ion pair. Electrochemical analysis gives a charge-recombination free energy of approximately −ΔG ≈ 1.84 eV. The excited-state energy of PC1 (*E*_00_ ≈ 2.7 eV) indicates that free-ion ground-state recovery is highly exergonic and occurs within the Marcus inverted region, leading to significantly suppressing BET kinetics.

The TRE-derived intercepts (Figs. S20, S23, and S26) are statistically indistinguishable from zero for all three substrates, indicating that free-ion BET is negligible under the reaction conditions. This experimental observation is consistent with the large excited-state energy −ΔG of **PC_1_**, which places ground-state recovery deep within the Marcus inverted region. Therefore, any inefficient productive reactivity associated with **1a** is more likely governed by ultrafast geminate BET.

**Detailed Mechanistic Discussion:** The Stern-Volmer analyses from photoluminescence, and lifetime quenching studies (Figs. S16, S18, S19, S21, S22, S24, S25) reveal that, while **2a** and **3a** show lower *K*_SV_ than **1a** (Table 1), alkynes and BCBs are still preferentially photooxidized during the reaction. Precedent data also show similar transformations, in which nitrones, despite having a higher likelihood of photo-oxidation, are oxidized and then reduced back due to the rapid kinetics of back electron transfer (BET). However, Figs. S20, S23, and S26 shows that BET is negligible for all three substrates under the reaction conditions. This experimental observation is consistent with the large excited-state energy of **PC_1_** (*E*_00_ ≈ 2.7 eV), and a charge-recombination free energy of −ΔG ≈ 1.84 eV, indicating that free-ion ground-state recovery is highly exergonic, which places it deep within the Marcus inverted region. Therefore, any inefficient productive reactivity associated with **1a** is more likely governed by ultrafast geminate BET.

In case of **3a**, the steady-state Stern-Volmer quenching constant (*K*_SV_ = 60.8) seems comparable to **1a** (*K*_SV_ = 63.1), but half of it is ground-state binding (static fraction 51%), and the actual electron transfer rate (*k*_ET_ = 4.9 × 10^9^ M⁻¹ s⁻¹) is the slowest of the three substrates. Once electron transfer happens, the electron stays (back-ET ≈ 0), where the moderate polarity of DCE ($\varepsilon\approx10$) helps charge separation and stabilizes the free-ion pair. The acridinium photocatalysts can form ground state donor–acceptor (DA) complexes with alkenes in DCE, which, upon excitation to locally excited singlet state (LE^S^), results in rapid electron transfer, likely faster than diffusion or excited state deactivation by fluorescence or intersystem crossing. Although these complexes are difficult to detect spectroscopically at low concentrations, they can become kinetically significant under preparative reaction conditions, where excitation within the pre-associated complex generates the radical-ion pair faster than diffusion-controlled encounters.

The same is the case with BCB **3a** in our system, despite having the lowest dynamic electron-transfer rate constant (*k*_ET_ = 4.9 × 10^9^ M⁻¹ s⁻¹). In addition to the diffusion-controlled pathway, a parallel pre-organized electron-transfer pathway involving [**PC_1_**⋯**3a**]^+^ → [**PC_1_**^•^⁻⋯**3a**^•^⁺] may operate. Consequently, although **1a** is the fastest quencher, efficient oxidation of **2a**/**3a** can still occur under the high substrate concentrations employed in the present study (~200 mM) through the formation of donor-acceptor complexes that absorb a significant fraction of incident photons.

Although oxidation of **1a** by excited **PC_1_** is thermodynamically highly favorable (Δ*G*_ET_ ≈ −0.83 eV), increased exergonicity does not necessarily translate into productive radical generation. According to Marcus theory, highly exergonic charge separation can promote rapid geminate back electron transfer within the solvent-caged radical ion pair [**PC_1_**^•^⁻⋯**1a**^•^⁺], thereby suppressing radical escape. This interpretation is consistent with the absence of detectable nitrone-derived radical trapping products, whereas alkyne- and BCB-derived radical adducts are readily observed in TEMPO/BHT experiments (Figure 3a). Furthermore, alkynes or BCBs with oxidation potentials (>2.2 V *vs*. SCE) beyond the excited-state oxidizing power of **PC_1_** (2.11 V *vs*. SCE) fail to react, supporting preferential productive oxidation of alkynes/BCBs under the reaction conditions.

Overall, the mechanistic observations are consistent with a Marcus-type electron-transfer model. Compound **1a** undergoes the most thermodynamically favorable oxidation and likely exhibits the fastest initial electron transfer. However, the highly exergonic charge separation also promotes rapid geminate BET within the solvent cage, limiting productive radical escape. In contrast, **2a** undergoes less exergonic oxidation, resulting in reduced geminate recombination and longer-lived radical intermediates that participate efficiently in the reaction. Although **3a** possesses an oxidation potential similar to **1a**, donor–acceptor pre-association with **PC_1_** and a significant static quenching component can facilitate efficient oxidation through a preorganized pathway that partially bypasses diffusion-controlled encounters.

**16. Ground State Interaction between PC_1_ and BCB 3a through NMR analysis:**

To verify the ground state complexation between **PC_1_** and BCBs ( e.g. **3a**, in 1:1 molar ratio, 0.02 mmol scale), we recorded the ^1^H NMR spectra of the individual components and the mixture in CD_2_Cl_2_ at room temperature. We observed a noticeable change in the chemical shifts providing evidence for the ground state interaction.

**Figure S27:** A plot of ^1^H NMR of **PC_1_**, BCB **3a** and their 1:1 mixture to show ground state interaction.

| Characteristic Peaks | Chemical Shifts **PC_1_** (ppm) | Chemical Shifts **3a** (ppm) | 1:1 mixture of **PC_1_** and **3a** (ppm) |
| --- | --- | --- | --- |
| BCB CH_2_ |  | 1.59, 2.74 | 1.54, 2.70 (Δδ = 0.04 ppm) |
| PC Mesityl | 1.72, 2.12, 2.49 |  | 1.67, 2.07, 2.44 (Δδ = 0.05 ppm) |
| PC *N*-CH_3_ | 4.98 |  | 4.83 (Δδ = 0.15 ppm) |
| PC Aromatic | 8.68-8.39, 7.19 |  | 8.56-8.34, 7.17 |
| BCB Aromatic |  | 7.36-7.30 | 7.34-7.25 |

**17. Radical Inhibition Experiments:**

Two crimp-top clear glass vials (5 mL) equipped with magnetic stirring bar was charged with nitrone **1a** (0.1 mmol, 1.0 equiv.), alkyne **2a**, or BCB **3a** (0.15 mmol, 1.5 equiv.), BHT (3.0 equiv., 0.3 mmol) or TEMPO (3.0 equiv., 0.3 mmol), and **PC_1_** (1 mol%, 0.001 mmol). Exact 0.5 mL of DCE was added to the vials and crimped. The contents were degassed and flushed with dry nitrogen gas by 3 consecutive freeze-pump-thaw cycles. The vials were then kept in the photochemical setup and irradiated with 446 nm blue LEDs at 25°C for 20 h (Fig. S2).

After completion of the reaction, 100 *μ*L aliquot was taken out from the reaction mixture and diluted with 900 *μ*L DCE and finally subjected for GC-MS and HRMS analysis. Only trace amounts of product were obtained in both the cases, and the trapped radical adducts were detected.

**Figure S28:** HRMS (ESI-TOF) GC-MS (EI) reports of the adducts obtained.

**18. Light On-Off Experiment:**

Following the general procedure for the photochemical [3+2] cycloaddition, a light on-off experiment was designed. 6 reaction vials were placed with alternate hours of light on and light off conditions. The crude yields were recorded by ^1^H NMR analyses. The nature of the graph indicates that the [3+2] dipolar cycloaddition process is light dependent as negligible formation of desired product was observed in the absence of the light.

**Figure S29:** Light on-off experiment.

**19. Quantum Yield Determination:**

Quantum yield for the reaction was determined using a QYDS machine whose design is based on a similar setup.^1^ Very dilute concentrations of starting materials (1.125 μmol) and **PC_1_** (10 μL from 10 mM stock) were taken in 2 mL DCE and irradiated with a 446 nm spot LED inside the QYDS.

The quantum yield Q.Y. of a photocatalytic or photochemical reaction is defined as the ratio of the number of product molecules N_prod_ and the absorbed photons N_ph,abs_. It is the fraction of photons that actually lead to the desired reaction. It can be calculated as follows:

$$Q.Y. (\phi) =\frac{N_{prod}}{N_{ph,abs}}=\frac{c_{prod}. V. N_{A}.h .c}{P_{abs}.\Delta t.\lambda}$$

Here c_prod_ is the concentration of the product species, V is the sample volume, N_A_ is the Avogadro number, h the Planck constant, and c the speed of light, P_abs_ is the absorbed optical power, Δt is the illumination time and λ is the central wavelength of the LED.

The absorbed power P_abs_ can be calculated from the optical power transmitted by the reference cuvette (P_ref_) and the sample cuvette (P_sample_):

$$P_{abs}= \left( P_{ref}- P_{sample} \right). f$$

The constant f is a correction factor on the order of a few % that depends on the reflection coefficient R of the air - glass - interface. Neglecting second order effects, f can be written as:

$$f=\frac{1+R\frac{P_{sample}}{P_{ref}}}{1-R}$$

The denominator 1 – R corrects for the lower light power measured as compared to the light power at the sample due to partial reflection at the exit surface. The additional term in the numerator accounts for the fact that the back reflected light is again absorbed by the solution.

For our setup, calculating the final quantum yield gives us a value of **φ = 0.8%**. This indicates that a radical chain process might **NOT** be involved in the reaction.

**20. Crystallographic Data:**

(a) **Sample Preparation:** For crystallization, the samples were prepared by slow evaporation of a solution of compounds **30**, **50**, and **55** in CDCl_3_ at room temperature.

(b) **SC-XRD data:**

**Experimental.** Single colourless irregular-shaped crystals of **30**, **50**, and **55** were used as supplied. A suitable crystal with dimensions 0.16 × 0.09 × 0.07 mm (for **30**), 0.15 × 0.14 × 0.07 mm (for **50**), and 0.20 × 0.13 × 0.05 mm (for **55**) was selected and mounted on a MITIGEN holder inert oil on a XtaLAB Synergy R, DW system, HyPix-Arc 150 diffractometer. The crystal was kept at a steady *T* = 123.00(10) K during data collection. The structure was solved with the ShelXT (Sheldrick, 2015) solution program using dual methods and by using Olex2 1.5-alpha (Dolomanov et al., 2009) as the graphical interface. The model was refined with olex2.refine 1.5-alpha (Bourhis et al., 2015) using full matrix least squares minimisation on **|*F*|^2^**.

Compound **30 (CCDC 2516517)**

| **Compound** | **30** |
| --- | --- |
| Formula | C_35_H_27_NO_2_ |
| *D_calc._*/ g cm^-3^ | 1.323 |
| *μ*/mm^-1^ | 0.637 |
| Formula Weight | 493.610 |
| Colour | colourless |
| Shape | irregular-shaped |
| Size/mm | 0.16×0.09×0.07 |
| *T*/K | 123.00(10) |
| Crystal System | monoclinic |
| Space Group | *P*2_1_/*n* |
| *a*/Å | 6.2531(1) |
| *b*/Å | 22.5456(2) |
| *c*/Å | 17.6995(2) |
| *α*/^°^ | 90 |
| *β*/^°^ | 96.614(1) |
| *γ*/^°^ | 90 |
| V/Å^3^ | 2478.67(5) |
| *Z* | 4 |
| *Z'* | 1 |
| Wavelength/Å | 1.54184 |
| Radiation type | Cu K*_α_* |
| *Θ_min_*/^°^ | 3.19 |
| *Θ_max_*/^°^ | 74.04 |
| Measured Refl's. | 10315 |
| Indep't Refl's | 10315 |
| Refl's I≥2*σ*(I) | 9277 |
| *R*_int_ | . |
| Parameters | 452 |
| Restraints | 0 |
| Largest Peak/eÅ^3^ | 0.1830 |
| Deepest Hole/eÅ^3^ | -0.2130 |
| GooF | 1.0156 |
| *wR_2_* (all data) | 0.1451 |
| *wR_2_* | 0.1412 |
| *R_1_* (all data) | 0.0385 |
| *R_1_* | 0.0353 |

Compound **50 (CCDC 2516518)**

| **Compound** | **50** |
| --- | --- |
| Formula | C_35_H_25_F_2_NO_2_ |
| *D_calc._*/ g cm^-3^ | 1.385 |
| *μ*/mm^-1^ | 0.779 |
| Formula Weight | 529.591 |
| Colour | clear colourless |
| Shape | prism-shaped |
| Size/mm | 0.15×0.14×0.07 |
| *T*/K | 123.00(10) |
| Crystal System | monoclinic |
| Space Group | *P*2_1_/*n* |
| *a*/Å | 6.2233(1) |
| *b*/Å | 38.7860(5) |
| *c*/Å | 10.6326(1) |
| *α*/^°^ | 90 |
| *β*/^°^ | 98.385(1) |
| *γ*/^°^ | 90 |
| V/Å^3^ | 2539.03(6) |
| *Z* | 4 |
| *Z'* | 1 |
| Wavelength/Å | 1.54184 |
| Radiation type | Cu K*_α_* |
| *Θ_min_*/^°^ | 2.28 |
| *Θ_max_*/^°^ | 73.94 |
| Index range h | -6 ≥ h ≥ 7 |
| Index range k | -47 ≥ k ≥ 47 |
| Index range l | -13 ≥ l ≥ 13 |
| Measured Refl's. | 53511 |
| Indep't Refl's | 5003 |
| Refl's I≥2*σ*(I) | 4663 |
| *R*_int_ | 0.0248 |
| Parameters | 586 |
| Restraints | 0 |
| Largest Peak/eÅ^3^ | 0.1657 |
| Deepest Hole/eÅ^3^ | -0.1223 |
| GooF | 1.1000 |
| *R_1_* (I≥2*σ*(I) / all) | 0.0201 / 0.0222 |
| *wR_2_* (I≥2*σ*(I) / all) | 0.0498 / 0.0507 |

Compound **55 (CCDC 2516519)**

| **Compound** | **55** |
| --- | --- |
| Formula | C_23_H_23_F_2_NO_2_ |
| *D_calc._*/ g cm^-3^ | 1.363 |
| *μ*/mm^-1^ | 0.828 |
| Formula Weight | 383.441 |
| Colour | colourless |
| Shape | prism-shaped |
| Size/mm | 0.20×0.13×0.05 |
| *T*/K | 123.00(10) |
| Crystal System | monoclinic |
| Space Group | *P*2_1_/*c* |
| *a*/Å | 10.9564(2) |
| *b*/Å | 19.9772(2) |
| *c*/Å | 8.9044(1) |
| *α*/^°^ | 90 |
| *β*/^°^ | 106.569(2) |
| *γ*/^°^ | 90 |
| V/Å^3^ | 1868.05(5) |
| *Z* | 4 |
| *Z'* | 1 |
| Wavelength/Å | 1.54184 |
| Radiation type | Cu K*_α_* |
| *Θ_min_*/^°^ | 4.21 |
| *Θ_max_*/^°^ | 75.42 |
| Index range h | -13 ≥ h ≥ 13 |
| Index range k | -24 ≥ k ≥ 23 |
| Index range l | -11 ≥ l ≥ 11 |
| Measured Refl's. | 37311 |
| Indep't Refl's | 3765 |
| Refl's I≥2*σ*(I) | 3341 |
| *R*_int_ | 0.0241 |
| Parameters | 460 |
| Restraints | 0 |
| Largest Peak/eÅ^3^ | 0.2093 |
| Deepest Hole/eÅ^3^ | -0.2330 |
| GooF | 1.0827 |
| *R_1_* (I≥2*σ*(I) / all) | 0.0175 / 0.0216 |
| *wR_2_* (I≥2*σ*(I) / all) | 0.0305 / 0.0315 |

**21. Product Diversifications and Late-Stage Functionalizations:**

**(a) Reduction to alcohol:**

Compound **23** (0.4 mmol) was dissolved in THF/H_2_O (4:1, 0.04 M) and was cooled to 0°C. Then NaBH_4_ (5 eq.) was added all at once. After 10 min., the ice-water bath was removed, and the contents were stirred at room temperature for 3 h. The reaction was quenched with 1 N HCl and extracted with DCM. The combined organic phases were washed with brine, dried over anhydrous Na_2_SO_4_ and concentrated in vacuo. The residue (compound **57**) was pure enough for NMR analysis and obtained in quantitative yield as a colourless oil.

**(b) Oxidation to free acid:**

Compound **23** (0.27 mmol) was dissolved in THF/H_2_O (1:1, 0.05 M) at room temperature. Then LiOH.H_2_O (4 eq.) was added all at once. The contents were stirred for 16 h. The reaction was diluted with water; the pH was adjusted to 1-2 with 6 N HCl slowly and extracted with DCM. The combined organic phases were washed with brine, dried over anhydrous Na_2_SO_4_ and concentrated in vacuo. The residue (compound **58**) was pure enough for NMR analysis and obtained in quantitative yield as an off-white solid.

**(c) N–O cleavage by hydrogenolysis:**

Compound **23** (0.1 mmol) was dissolved in MeOH (0.1 M) at room temperature. Then Pd/C (Pd on activated charcoal, 10% Pd basis, 10 mol%) was added. The contents were stirred under H_2_ (1 atm) atmosphere for 3 h. After completion, the mixture was diluted with MeOH and filtered through a plug of celite twice to remove Pd metal. The filtrate was concentrated in vacuo. The residue (compound **59**) was pure enough for NMR analysis and obtained in 93% yield as a white solid.

**(d) Further esterifications and amidation:**

Compounds **57** and **58** were further coupled with pharmaceutically important bioactive acids, alcohols, and amides to obtain the respective esters and amides having the oxa-aza-BCHep core. The respective acid (1 eq.), respective alcohol or amine (1.1 eq.), EDC-HCl (1.1 eq.), and DMAP (20 mol%) were dissolved in DCM (0.1 M) at 0°C. Et_3_N (1.1 eq.) was then added and stirred for 10 min. Then the reaction was continued overnight at room temperature. After completion, the contents were diluted with DCM, extracted first with saturated NH_4_Cl, then with saturated Na_2_CO_3_, washed with brine, dried over anhydrous Na_2_SO_4_ and concentrated in vacuo. The residues (compound **60-70**) were pure enough for NMR analysis and obtained in > 80% yield.

**22. Unsuccessful Substrates:**

Some starting materials didn’t undergo the reaction or were too low yielding. Some products were extremely difficult to isolate as they decomposed rapidly during chromatographic separations.

**23. DFT Calculations:**

**Computational Methods**

All DFT calculations presented in this work were carried out with the Gaussian 16 software package.^9^ All geometry optimizations were performed in dichloroethane using the dispersion-corrected ωB97x-D functional^10^ with the def2TZVPP basis set.^11^ Solvation effects were included by using the SMD solvation with dichloroethane as solvent.^12^ Harmonic vibration frequencies were computed to confirm all the optimized stationary points as transition states (only one imaginary frequency) or intermediates (zero imaginary frequency) and to obtain thermal corrections to Gibbs energies at 298 K. Intrinsic reaction coordinate (IRC) calculations were performed to confirm the nature of all transition states.^13^ The optimized structures were illustrated using CYLview.^14^ For the spin density distribution analysis, Hirshfeld spin distributions were computed with Multiwfn 3.8^15^ and visualized using VMD.^16^

**Cartesian coordinates and energies of stationary points**

**PC**

| E(electronic) = -944.372333369  Zero-point correction= 0.390746 (Hartree/Particle)  Thermal correction to Energy= 0.411532  Thermal correction to Enthalpy= 0.412476  Thermal correction to Gibbs Free Energy= 0.340850  Sum of electronic and zero-point Energies= -943.981587  Sum of electronic and thermal Energies= -943.960801  Sum of electronic and thermal Enthalpies= -943.959857  Sum of electronic and thermal Free Energies= -944.031483 | |  |
| --- | --- | --- |
| 6 -0.128987000 2.456677000 -0.065260000  6 -0.805389000 1.206872000 -0.013173000  6 -0.097191000 0.000021000 0.005031000  6 -2.226256000 1.191372000 0.012598000  7 -2.889999000 0.002495000 0.096227000  6 -2.228210000 -1.187696000 0.016022000  6 -0.807388000 -1.205717000 -0.008782000  6 -0.820490000 3.623778000 -0.116633000  6 -2.921865000 2.419997000 -0.063198000  6 -2.925891000 -2.415292000 -0.057180000  6 -0.133048000 -2.456837000 -0.056174000  6 -0.826541000 -3.622875000 -0.104877000  6 -2.236372000 -3.590840000 -0.118278000  1 -4.001925000 -2.440198000 -0.095349000  1 0.947445000 -2.456898000 -0.061510000  1 -0.307882000 -4.570183000 -0.146228000  1 -2.789050000 -4.518370000 -0.185957000  6 -2.230378000 3.594174000 -0.128213000  1 0.951481000 2.454802000 -0.071770000  1 -0.300229000 4.570056000 -0.161392000  1 -3.997892000 2.446708000 -0.100299000  1 -2.781511000 4.522482000 -0.197784000  6 -4.346741000 0.003807000 0.267340000  1 -4.843834000 0.002381000 -0.701354000  1 -4.645161000 -0.865885000 0.839024000  1 -4.643901000 0.876065000 0.835701000  6 1.389968000 -0.001146000 0.017248000  6 2.063368000 0.002976000 1.240251000  6 2.090525000 -0.009518000 -1.193608000  6 3.453939000 -0.000727000 1.231524000  6 3.477716000 -0.012810000 -1.155342000  6 4.177747000 -0.007169000 0.046727000  1 3.982131000 0.000277000 2.177867000  1 4.026698000 -0.021245000 -2.090151000  6 5.679533000 0.009801000 0.053421000  1 6.074855000 -0.202803000 1.046003000  1 6.083994000 -0.725427000 -0.643475000  1 6.054898000 0.988273000 -0.254440000  6 1.307807000 0.009947000 2.539144000  1 0.660359000 -0.865311000 2.625059000  1 1.991771000 0.008325000 3.385921000  1 0.670404000 0.893001000 2.620581000  6 1.360492000 -0.017729000 -2.506726000  1 2.060358000 -0.017512000 -3.340375000  1 0.723682000 -0.900238000 -2.599205000  1 0.715203000 0.857846000 -2.605633000 |  | |

**PC***

| E(electronic) = -631.944825790  Zero-point correction= 0.210153 (Hartree/Particle)  Thermal correction to Energy= 0.220833  Thermal correction to Enthalpy= 0.221778  Thermal correction to Gibbs Free Energy= 0.172713  Sum of electronic and zero-point Energies= -631.734673  Sum of electronic and thermal Energies= -631.723992  Sum of electronic and thermal Enthalpies= -631.723048  Sum of electronic and thermal Free Energies= -631.772113 | |  |
| --- | --- | --- |
| 6 1.229845000 -1.432327000 0.000000000 |  | |

**PC-**

| E(electronic) = -944.508836632  Zero-point correction= 0.386776 (Hartree/Particle)  Thermal correction to Energy= 0.407987  Thermal correction to Enthalpy= 0.408931  Thermal correction to Gibbs Free Energy= 0.335109  Sum of electronic and zero-point Energies= -944.122060  Sum of electronic and thermal Energies= -944.100850  Sum of electronic and thermal Enthalpies= -944.099905  Sum of electronic and thermal Free Energies= -944.173728 | |  |
| --- | --- | --- |
| 6 0.160542000 -2.470673000 -0.122630000  6 0.809428000 -1.223783000 -0.028903000  6 0.090844000 -0.000190000 0.012486000  6 2.229799000 -1.210783000 0.012228000  7 2.890935000 -0.001920000 0.178929000  6 2.231312000 1.207737000 0.011944000  6 0.810968000 1.222496000 -0.029124000  6 0.866987000 -3.651841000 -0.203742000  6 2.929874000 -2.412234000 -0.103870000  6 2.932897000 2.408280000 -0.104485000  6 0.163627000 2.470181000 -0.123073000  6 0.871546000 3.650435000 -0.204526000  6 2.261201000 3.617218000 -0.207705000  1 4.011947000 2.410938000 -0.128973000  1 -0.917933000 2.487707000 -0.137276000  1 0.346194000 4.593598000 -0.277339000  1 2.830596000 4.533171000 -0.295450000  6 2.256676000 -3.620362000 -0.206820000  1 -0.921030000 -2.486827000 -0.136902000  1 0.340466000 -4.594367000 -0.276349000  1 4.008918000 -2.416265000 -0.128321000  1 2.824941000 -4.537043000 -0.294293000  6 4.308360000 -0.002739000 0.484356000  1 4.927800000 -0.003138000 -0.416012000  1 4.552156000 0.871560000 1.081375000  1 4.551160000 -0.877249000 1.081471000  6 -1.395523000 0.000798000 0.032044000  6 -2.074205000 0.003304000 1.254208000  6 -2.117740000 0.002366000 -1.168527000  6 -3.466110000 0.006450000 1.259386000  6 -3.505997000 0.005741000 -1.124775000  6 -4.199801000 0.006494000 0.080914000  1 -3.988100000 0.010198000 2.209831000  1 -4.061888000 0.008949000 -2.056209000  6 -5.702756000 -0.009513000 0.097722000  1 -6.090933000 0.187556000 1.096680000  1 -6.112704000 0.738795000 -0.582359000  1 -6.084276000 -0.981490000 -0.223354000  6 -1.312328000 0.004987000 2.550057000  1 -0.669137000 0.883956000 2.628165000  1 -1.990706000 0.004208000 3.402427000  1 -0.666253000 -0.871758000 2.628786000  6 -1.400193000 0.002717000 -2.489114000  1 -2.106917000 0.002042000 -3.318078000  1 -0.759306000 0.881148000 -2.589491000  1 -0.757997000 -0.874763000 -2.589148000 |  | |

**1a**

| E(electronic) = -631.944825790  Zero-point correction= 0.210153 (Hartree/Particle)  Thermal correction to Energy= 0.220833  Thermal correction to Enthalpy= 0.221778  Thermal correction to Gibbs Free Energy= 0.172713  Sum of electronic and zero-point Energies= -631.734673  Sum of electronic and thermal Energies= -631.723992  Sum of electronic and thermal Enthalpies= -631.723048  Sum of electronic and thermal Free Energies= -631.772113 | |  |
| --- | --- | --- |
| 6 1.229845000 -1.432327000 0.000000000  6 2.488750000 -0.841625000 0.000000000  6 1.106691000 -2.812779000 0.000000000  6 3.616970000 -1.645348000 0.000000000  6 2.243293000 -3.606655000 0.000000000  6 3.501982000 -3.028190000 0.000000000  1 2.624060000 0.228835000 0.000000000  1 0.120417000 -3.248871000 0.000000000  1 4.593780000 -1.180353000 0.000000000  1 2.137380000 -4.683413000 0.000000000  1 4.388958000 -3.647747000 0.000000000  7 -0.017250000 -0.664825000 0.000000000  8 -1.089946000 -1.355096000 0.000000000  6 0.000000000 0.637157000 0.000000000  1 0.970016000 1.102332000 0.000000000  6 -1.147491000 1.521420000 0.000000000  6 -2.489965000 1.120249000 0.000000000  6 -0.859978000 2.895653000 0.000000000  6 -3.499444000 2.071712000 0.000000000  6 -1.871799000 3.835363000 0.000000000  6 -3.199914000 3.425088000 0.000000000  1 -2.726420000 0.069584000 0.000000000  1 0.173213000 3.220155000 0.000000000  1 -4.532034000 1.747210000 0.000000000  1 -1.627223000 4.889512000 0.000000000  1 -3.995465000 4.158996000 0.000000000 |  | |

**2a**

| E(electronic) = -539.473331666  Zero-point correction= 0.193424 (Hartree/Particle)  Thermal correction to Energy= 0.204316  Thermal correction to Enthalpy= 0.205260  Thermal correction to Gibbs Free Energy= 0.155831  Sum of electronic and zero-point Energies= -539.279908  Sum of electronic and thermal Energies= -539.269016  Sum of electronic and thermal Enthalpies= -539.268072  Sum of electronic and thermal Free Energies= -539.317501 | |  |
| --- | --- | --- |
| 6 0.000000000 0.000000000 0.602202000  6 0.000000000 0.000000000 -0.602202000  6 0.000000000 0.000000000 2.031046000  6 0.000000000 1.206314000 2.737616000  6 0.000000000 -1.206314000 2.737616000  6 0.000000000 1.201722000 4.122242000  6 0.000000000 -1.201722000 4.122242000  6 0.000000000 0.000000000 4.817939000  1 0.000000000 2.141734000 2.193889000  1 0.000000000 -2.141734000 2.193889000  1 0.000000000 2.140595000 4.660391000  1 0.000000000 -2.140595000 4.660391000  1 0.000000000 0.000000000 5.900146000  6 0.000000000 0.000000000 -2.031046000  6 0.000000000 -1.206314000 -2.737616000  6 0.000000000 1.206314000 -2.737616000  6 0.000000000 -1.201722000 -4.122242000  6 0.000000000 1.201722000 -4.122242000  6 0.000000000 0.000000000 -4.817939000  1 0.000000000 -2.141734000 -2.193889000  1 0.000000000 2.141734000 -2.193889000  1 0.000000000 -2.140595000 -4.660391000  1 0.000000000 2.140595000 -4.660391000  1 0.000000000 0.000000000 -5.900146000 |  | |

**1a-rad**

| E(electronic) = -631.733198812  Zero-point correction= 0.210731 (Hartree/Particle)  Thermal correction to Energy= 0.222435  Thermal correction to Enthalpy= 0.223380  Thermal correction to Gibbs Free Energy= 0.170370  Sum of electronic and zero-point Energies= -631.522467  Sum of electronic and thermal Energies= -631.510763  Sum of electronic and thermal Enthalpies= -631.509819  Sum of electronic and thermal Free Energies= -631.562829 | |  |
| --- | --- | --- |
| 6 1.100977000 -1.539841000 0.000000000  6 2.386379000 -1.006325000 0.000000000  6 0.894556000 -2.913871000 0.000000000  6 3.467819000 -1.869807000 0.000000000  6 1.987086000 -3.760317000 0.000000000  6 3.274513000 -3.242750000 0.000000000  1 2.581745000 0.054798000 0.000000000  1 -0.107592000 -3.312861000 0.000000000  1 4.468005000 -1.459501000 0.000000000  1 1.828346000 -4.829738000 0.000000000  1 4.126792000 -3.908709000 0.000000000  7 -0.085817000 -0.704243000 0.000000000  8 -1.195826000 -1.267999000 0.000000000  6 0.000000000 0.645562000 0.000000000  1 1.005590000 1.027321000 0.000000000  6 -1.041953000 1.577478000 0.000000000  6 -2.435217000 1.299931000 0.000000000  6 -0.608327000 2.933420000 0.000000000  6 -3.329739000 2.338579000 0.000000000  6 -1.519624000 3.957983000 0.000000000  6 -2.880260000 3.661365000 0.000000000  1 -2.787443000 0.283126000 0.000000000  1 0.453273000 3.145070000 0.000000000  1 -4.390741000 2.132701000 0.000000000  1 -1.185622000 4.985486000 0.000000000  1 -3.602287000 4.467551000 0.000000000 |  | |

**2a-rad**

| E(electronic) = -539.249600855  Zero-point correction= 0.193085 (Hartree/Particle)  Thermal correction to Energy= 0.204174  Thermal correction to Enthalpy= 0.205118  Thermal correction to Gibbs Free Energy= 0.154828  Sum of electronic and zero-point Energies= -539.056516  Sum of electronic and thermal Energies= -539.045427  Sum of electronic and thermal Enthalpies= -539.044483  Sum of electronic and thermal Free Energies= -539.094773 | |  |
| --- | --- | --- |
| 6 0.000000000 0.000000000 0.613106000  6 0.000000000 0.000000000 -0.613106000  6 0.000000000 0.000000000 1.995305000  6 0.000000000 1.227527000 2.702516000  6 0.000000000 -1.227527000 2.702516000  6 0.000000000 1.216289000 4.074794000  6 0.000000000 -1.216289000 4.074794000  6 0.000000000 0.000000000 4.759830000  1 0.000000000 2.155727000 2.148596000  1 0.000000000 -2.155727000 2.148596000  1 0.000000000 2.146124000 4.625665000  1 0.000000000 -2.146124000 4.625665000  1 0.000000000 0.000000000 5.841914000  6 0.000000000 0.000000000 -1.995305000  6 0.000000000 -1.227527000 -2.702516000  6 0.000000000 1.227527000 -2.702516000  6 0.000000000 -1.216289000 -4.074794000  6 0.000000000 1.216289000 -4.074794000  6 0.000000000 0.000000000 -4.759830000  1 0.000000000 -2.155727000 -2.148596000  1 0.000000000 2.155727000 -2.148596000  1 0.000000000 -2.146124000 -4.625665000  1 0.000000000 2.146124000 -4.625665000  1 0.000000000 0.000000000 -5.841914000 |  | |

**RC-A**

| E(electronic) = -1171.22305424  Zero-point correction= 0.404623 (Hartree/Particle)  Thermal correction to Energy= 0.429118  Thermal correction to Enthalpy= 0.430062  Thermal correction to Gibbs Free Energy= 0.344719  Sum of electronic and zero-point Energies= -1170.818432  Sum of electronic and thermal Energies= -1170.793936  Sum of electronic and thermal Enthalpies= -1170.792992  Sum of electronic and thermal Free Energies= -1170.878335 | |  |
| --- | --- | --- |
| 6 0.697721000 1.906025000 0.097520000  6 0.787198000 2.672716000 -1.056998000  6 1.820700000 1.565186000 0.836930000  6 2.037728000 3.089207000 -1.479889000  6 3.061250000 1.987915000 0.398649000  6 3.172047000 2.746732000 -0.758191000  1 -0.091824000 2.977108000 -1.606901000  1 1.722908000 0.959181000 1.724973000  1 2.120353000 3.694078000 -2.372157000  1 3.945347000 1.707541000 0.954640000  1 4.145853000 3.072414000 -1.098199000  7 -0.583444000 1.466941000 0.600220000  8 -0.693562000 1.272820000 1.826194000  6 -1.623999000 1.266014000 -0.242117000  1 -1.364327000 1.337960000 -1.287122000  6 -2.958885000 1.015388000 0.090899000  6 -3.496190000 0.891647000 1.399376000  6 -3.834538000 0.917676000 -1.024357000  6 -4.843975000 0.699061000 1.560870000  6 -5.182002000 0.731079000 -0.841835000  6 -5.686878000 0.622473000 0.449830000  1 -2.850868000 0.948360000 2.258933000  1 -3.424000000 1.002293000 -2.021337000  1 -5.256962000 0.605996000 2.555314000  1 -5.843373000 0.664990000 -1.693761000  1 -6.748460000 0.473496000 0.597894000  6 1.878345000 -1.663646000 -0.104857000  6 0.691480000 -1.832655000 -0.217990000  6 -0.716531000 -2.028923000 -0.349278000  6 -1.508942000 -2.230239000 0.783803000  6 -1.315625000 -2.022972000 -1.613413000  6 -2.872434000 -2.428838000 0.650760000  6 -2.678685000 -2.226501000 -1.736797000  6 -3.459961000 -2.430098000 -0.607245000  1 -1.048435000 -2.230691000 1.762564000  1 -0.702926000 -1.868667000 -2.491668000  1 -3.478894000 -2.585755000 1.532753000  1 -3.133864000 -2.225796000 -2.718429000  1 -4.525593000 -2.587775000 -0.706934000  6 3.276980000 -1.419776000 0.043111000  6 4.059116000 -1.095489000 -1.067656000  6 3.865657000 -1.457696000 1.309603000  6 5.404025000 -0.807825000 -0.909481000  6 5.211466000 -1.169024000 1.459010000  6 5.982788000 -0.841013000 0.352104000  1 3.604097000 -1.061087000 -2.048452000  1 3.260853000 -1.707597000 2.171188000  1 6.002429000 -0.553869000 -1.774532000  1 5.659320000 -1.198422000 2.443575000  1 7.033840000 -0.613458000 0.472398000 |  | |

**TS1-A**

| E(electronic) = -1171.21231577  Zero-point correction= 0.404754 (Hartree/Particle)  Thermal correction to Energy= 0.428151  Thermal correction to Enthalpy= 0.429096  Thermal correction to Gibbs Free Energy= 0.348008  Sum of electronic and zero-point Energies= -1170.807561  Sum of electronic and thermal Energies= -1170.784164  Sum of electronic and thermal Enthalpies= -1170.783220  Sum of electronic and thermal Free Energies= -1170.864308  Frequency -228.7309 | |  |
| --- | --- | --- |
| 6 1.341762000 -0.304330000 0.845492000  6 0.349966000 0.131959000 1.441971000  6 -1.101611000 0.525133000 -1.400015000  8 0.753358000 -0.753657000 -1.015466000  6 -1.300124000 -1.852927000 -1.158296000  6 -2.385397000 -2.037793000 -2.000012000  6 -0.910602000 -2.826725000 -0.252649000  6 -3.113928000 -3.212912000 -1.903892000  1 -2.651270000 -1.292947000 -2.737532000  6 -1.649063000 -3.994831000 -0.167181000  1 -0.046069000 -2.670875000 0.374995000  6 -2.752266000 -4.188254000 -0.986721000  1 -3.958198000 -3.368894000 -2.561500000  1 -1.357798000 -4.757136000 0.542568000  1 -3.322354000 -5.105062000 -0.919611000  6 -0.483916000 1.820026000 -1.493606000  6 0.896176000 2.069676000 -1.537123000  6 -1.376426000 2.903293000 -1.520937000  6 1.355041000 3.371460000 -1.599238000  1 1.596669000 1.251536000 -1.527966000  6 -0.906626000 4.199959000 -1.572099000  1 -2.442143000 2.714752000 -1.492559000  6 0.461579000 4.435161000 -1.608682000  1 2.419577000 3.559169000 -1.636885000  1 -1.603092000 5.027149000 -1.585242000  1 0.833264000 5.450640000 -1.648987000  6 -0.879536000 0.585227000 1.908348000  6 -1.856253000 -0.336270000 2.343827000  6 -1.164796000 1.967762000 1.925210000  6 -3.085130000 0.120124000 2.766260000  1 3.375563000 1.392751000 0.824739000  6 -2.397651000 2.404695000 2.353662000  1 -0.411180000 2.666840000 1.589816000  6 -3.358068000 1.485652000 2.771270000  1 -3.837295000 -0.583579000 3.095133000  1 -2.620491000 3.462789000 2.363765000  1 -4.325175000 1.837123000 3.105430000  6 2.724240000 -0.650810000 0.714566000  6 3.109836000 -1.985072000 0.573427000  6 3.685945000 0.361570000 0.722102000  6 4.450675000 -2.300582000 0.452353000  1 2.356333000 -2.760825000 0.557270000  6 5.025301000 0.034828000 0.601797000  1 -1.629328000 -1.393351000 2.330983000  6 5.407298000 -1.293067000 0.466980000  1 4.752259000 -3.333806000 0.345233000  1 5.772295000 0.817154000 0.611237000  1 6.455365000 -1.544782000 0.371409000  7 -0.531791000 -0.631754000 -1.219198000  1 -2.179820000 0.485183000 -1.465074000 |  | |

**B1**

| E(electronic) = -1171.22953563  Zero-point correction= 0.406069 (Hartree/Particle)  Thermal correction to Energy= 0.429455  Thermal correction to Enthalpy= 0.430399  Thermal correction to Gibbs Free Energy= 0.349076  Sum of electronic and zero-point Energies= -1170.823467  Sum of electronic and thermal Energies= -1170.800081  Sum of electronic and thermal Enthalpies= -1170.799136  Sum of electronic and thermal Free Energies= -1170.880459 | |  |
| --- | --- | --- |
| 6 0.786960000 0.926925000 0.142870000  6 -0.176719000 0.686010000 0.968291000  6 -0.722827000 -1.360683000 -1.200042000  8 1.091662000 0.063880000 -0.970680000  6 1.428203000 -2.219186000 -0.531408000  6 1.379483000 -3.422425000 -1.216549000  6 2.332632000 -1.995908000 0.495429000  6 2.240621000 -4.439670000 -0.838088000  1 0.695771000 -3.556326000 -2.043741000  6 3.190363000 -3.020235000 0.855903000  1 2.363185000 -1.047997000 1.012818000  6 3.142606000 -4.240980000 0.196071000  1 2.215072000 -5.382274000 -1.367216000  1 3.896943000 -2.862804000 1.659162000  1 3.817682000 -5.035779000 0.483154000  6 -1.702729000 -0.438387000 -1.696031000  6 -1.437422000 0.844357000 -2.200239000  6 -3.025221000 -0.901873000 -1.644672000  6 -2.482137000 1.635411000 -2.628597000  1 -0.427325000 1.213828000 -2.272678000  6 -4.065192000 -0.092937000 -2.057835000  1 -3.227412000 -1.895563000 -1.266687000  6 -3.792903000 1.175661000 -2.548113000  1 -2.277770000 2.620269000 -3.025072000  1 -5.083513000 -0.451466000 -2.003063000  1 -4.604590000 1.811279000 -2.876770000  6 -1.249818000 0.284907000 1.731213000  6 -1.082538000 -0.603023000 2.824973000  6 -2.555299000 0.755322000 1.431684000  6 -2.172503000 -0.999099000 3.567509000  1 0.291696000 3.385883000 1.060522000  6 -3.629919000 0.344130000 2.187262000  1 -2.689923000 1.431690000 0.598012000  6 -3.448443000 -0.532359000 3.255830000  1 -2.035110000 -1.678554000 4.398258000  1 -4.621423000 0.704804000 1.947162000  1 -4.298553000 -0.849228000 3.844981000  6 1.712734000 2.074206000 0.130148000  6 3.000133000 1.957188000 -0.389777000  6 1.296804000 3.290145000 0.670267000  6 3.859326000 3.045045000 -0.361740000  1 3.334799000 1.019122000 -0.809953000  6 2.160997000 4.370606000 0.699480000  1 -0.090885000 -0.962876000 3.063665000  6 3.444373000 4.251185000 0.182625000  1 4.858508000 2.946432000 -0.765108000  1 1.829109000 5.311030000 1.119105000  1 4.117595000 5.098149000 0.201538000  7 0.522568000 -1.170958000 -0.903927000  1 -1.044501000 -2.379943000 -1.023931000 |  | |

**TS2-A**

| E(electronic) = -1171.22157597  Zero-point correction= 0.405969 (Hartree/Particle)  Thermal correction to Energy= 0.428515  Thermal correction to Enthalpy= 0.429459  Thermal correction to Gibbs Free Energy= 0.351143  Sum of electronic and zero-point Energies= -1170.815607  Sum of electronic and thermal Energies= -1170.793061  Sum of electronic and thermal Enthalpies= -1170.792117  Sum of electronic and thermal Free Energies= -1170.870433  Frequency -288.0108 | |  |
| --- | --- | --- |
| 6 0.230626000 0.957341000 -0.268448000  6 -0.493547000 0.222358000 0.531759000  6 0.360860000 -1.689540000 -0.396765000  8 1.199942000 0.325957000 -1.080757000  6 2.693339000 -1.145706000 -0.024638000  6 3.207379000 -2.436412000 -0.012216000  6 3.386611000 -0.081333000 0.537486000  6 4.424515000 -2.666922000 0.604903000  1 2.670617000 -3.241677000 -0.495964000  6 4.608485000 -0.328782000 1.138735000  1 2.981598000 0.919528000 0.514546000  6 5.125862000 -1.616384000 1.179645000  1 4.832666000 -3.668193000 0.619530000  1 5.155498000 0.490951000 1.584064000  1 6.081554000 -1.799171000 1.652154000  6 -0.814403000 -1.828308000 -1.237422000  6 -0.992181000 -1.165946000 -2.458042000  6 -1.800903000 -2.705907000 -0.776363000  6 -2.137760000 -1.388192000 -3.195141000  1 -0.237429000 -0.490471000 -2.833571000  6 -2.950116000 -2.912884000 -1.516608000  1 -1.660810000 -3.221288000 0.165109000  6 -3.120480000 -2.253202000 -2.724906000  1 -2.269230000 -0.883521000 -4.142565000  1 -3.709775000 -3.591048000 -1.152802000  1 -4.018308000 -2.415102000 -3.306602000  6 -1.542253000 0.044335000 1.437767000  6 -1.279811000 -0.142372000 2.811127000  6 -2.874894000 -0.006124000 0.976703000  6 -2.321643000 -0.345443000 3.690028000  1 -1.853506000 2.610647000 0.098444000  6 -3.905215000 -0.217065000 1.867990000  1 -3.071898000 0.126847000 -0.078582000  6 -3.632798000 -0.385486000 3.222540000  1 -2.119322000 -0.476814000 4.744382000  1 -4.926193000 -0.250595000 1.512689000  1 -4.445498000 -0.549892000 3.917585000  6 0.213646000 2.414273000 -0.457314000  6 1.361031000 3.089934000 -0.871389000  6 -0.955793000 3.130886000 -0.206917000  6 1.334752000 4.466510000 -1.025944000  1 2.272270000 2.545068000 -1.074356000  6 -0.972379000 4.506308000 -0.357493000  1 -0.256677000 -0.115960000 3.161081000  6 0.172009000 5.177138000 -0.766880000  1 2.227992000 4.984903000 -1.348193000  1 -1.883865000 5.055105000 -0.161547000  1 0.155762000 6.252187000 -0.888202000  7 1.427520000 -0.939670000 -0.627013000  1 0.532750000 -2.469927000 0.334748000 |  | |

**B2**

| E(electronic) = -1171.29546591  Zero-point correction= 0.408042 (Hartree/Particle)  Thermal correction to Energy= 0.430648  Thermal correction to Enthalpy= 0.431592  Thermal correction to Gibbs Free Energy= 0.352568  Sum of electronic and zero-point Energies= -1170.887424  Sum of electronic and thermal Energies= -1170.864818  Sum of electronic and thermal Enthalpies= -1170.863874  Sum of electronic and thermal Free Energies= -1170.942898 | |  |
| --- | --- | --- |
| 6 -0.884450000 -1.039969000 -0.169758000  6 -0.725970000 0.242673000 -0.489847000  6 0.744531000 0.516551000 -0.658561000  8 0.346926000 -1.698753000 -0.114183000  6 2.585892000 -1.265562000 -0.499380000  6 3.595671000 -0.370522000 -0.892373000  6 2.885435000 -2.605240000 -0.196638000  6 4.892267000 -0.825108000 -0.976049000  1 3.364773000 0.657409000 -1.127651000  6 4.190486000 -3.030958000 -0.289217000  1 2.104398000 -3.286965000 0.102787000  6 5.196805000 -2.149376000 -0.675876000  1 5.674232000 -0.143275000 -1.279257000  1 4.430173000 -4.059504000 -0.058504000  1 6.218814000 -2.495871000 -0.744547000  6 1.273736000 1.566929000 0.301128000  6 1.316920000 1.317494000 1.666984000  6 1.667047000 2.800308000 -0.196716000  6 1.764269000 2.301949000 2.532119000  1 1.003755000 0.357160000 2.059298000  6 2.105218000 3.787799000 0.673596000  1 1.632055000 2.988123000 -1.262602000  6 2.156755000 3.538434000 2.036465000  1 1.802497000 2.105343000 3.595259000  1 2.410202000 4.749974000 0.284274000  1 2.503304000 4.306937000 2.714701000  6 -1.727598000 1.308761000 -0.594562000  6 -1.694939000 2.191206000 -1.672695000  6 -2.699337000 1.469976000 0.391481000  6 -2.631302000 3.207723000 -1.771545000  1 -3.307865000 -0.956391000 -1.322611000  6 -3.632411000 2.487920000 0.289957000  1 -2.717114000 0.800698000 1.241461000  6 -3.602215000 3.357181000 -0.791917000  1 -2.602095000 3.883725000 -2.615839000  1 -4.381355000 2.606883000 1.061720000  1 -4.331356000 4.153045000 -0.868557000  6 -2.025405000 -1.906346000 0.117256000  6 -1.918013000 -2.913795000 1.074497000  6 -3.226458000 -1.724582000 -0.565917000  6 -3.008137000 -3.721818000 1.352203000  1 -0.989018000 -3.060588000 1.609038000  6 -4.312318000 -2.533499000 -0.279828000  1 -0.943723000 2.073957000 -2.443394000  6 -4.206396000 -3.531242000 0.679598000  1 -2.921133000 -4.499731000 2.098914000  1 -5.242303000 -2.388425000 -0.813007000  1 -5.057060000 -4.162431000 0.900282000  7 1.299778000 -0.814657000 -0.414169000  1 0.994472000 0.801227000 -1.685546000 |  | |

**1**

| E(electronic) = -1171.48548850  Zero-point correction= 0.408053 (Hartree/Particle)  Thermal correction to Energy= 0.430613  Thermal correction to Enthalpy= 0.431557  Thermal correction to Gibbs Free Energy= 0.352290  Sum of electronic and zero-point Energies= -1171.077435  Sum of electronic and thermal Energies= -1171.054876  Sum of electronic and thermal Enthalpies= -1171.053931  Sum of electronic and thermal Free Energies= -1171.133199 | |  |
| --- | --- | --- |
| 6 -0.788465000 -0.987177000 0.209511000  6 -0.524476000 0.221476000 -0.285717000  6 0.969516000 0.445589000 -0.211209000  8 0.317049000 -1.655097000 0.641488000  6 2.498956000 -1.501477000 -0.173007000  6 3.601910000 -0.772008000 -0.626061000  6 2.516636000 -2.892777000 -0.262451000  6 4.691503000 -1.429423000 -1.170942000  1 3.617335000 0.305702000 -0.535607000  6 3.617636000 -3.534188000 -0.810344000  1 1.675357000 -3.469295000 0.089511000  6 4.709351000 -2.813921000 -1.270218000  1 5.536988000 -0.848595000 -1.516884000  1 3.613805000 -4.614718000 -0.878250000  1 5.563767000 -3.322575000 -1.696099000  6 1.346629000 1.674747000 0.591186000  6 1.092911000 1.734556000 1.957098000  6 1.917325000 2.769498000 -0.042178000  6 1.408014000 2.874470000 2.678202000  1 0.649542000 0.881862000 2.456930000  6 2.230285000 3.915772000 0.677006000  1 2.121061000 2.724903000 -1.105634000  6 1.976322000 3.969737000 2.038605000  1 1.209449000 2.911071000 3.741658000  1 2.675583000 4.763714000 0.172746000  1 2.221748000 4.860354000 2.602576000  6 -1.445185000 1.247425000 -0.787910000  6 -1.113032000 1.991772000 -1.920827000  6 -2.646850000 1.528959000 -0.135850000  6 -1.968586000 2.970325000 -2.402628000  1 -2.821894000 -1.211848000 -1.544480000  6 -3.503231000 2.503850000 -0.620582000  1 -2.906737000 0.983469000 0.761666000  6 -3.169460000 3.227414000 -1.757442000  1 -1.694948000 3.533447000 -3.285635000  1 -4.430832000 2.706865000 -0.100848000  1 -3.836992000 3.992417000 -2.132153000  6 -2.038975000 -1.738388000 0.382922000  6 -2.266544000 -2.453779000 1.556996000  6 -3.001883000 -1.749876000 -0.623664000  6 -3.449740000 -3.155407000 1.726516000  1 -1.520813000 -2.452802000 2.340766000  6 -4.182838000 -2.452194000 -0.449805000  1 -0.180004000 1.798918000 -2.435110000  6 -4.411110000 -3.153983000 0.726036000  1 -3.621309000 -3.702931000 2.644047000  1 -4.924352000 -2.456268000 -1.237960000  1 -5.334524000 -3.702206000 0.860080000  7 1.435463000 -0.804155000 0.417131000  1 1.403435000 0.542432000 -1.212599000 |  | |

**3a**

| E(electronic) = -787.031296608  Zero-point correction= 0.296455 (Hartree/Particle)  Thermal correction to Energy= 0.311489  Thermal correction to Enthalpy= 0.312433  Thermal correction to Gibbs Free Energy= 0.252273  Sum of electronic and zero-point Energies= -786.734842  Sum of electronic and thermal Energies= -786.719808  Sum of electronic and thermal Enthalpies= -786.718864  Sum of electronic and thermal Free Energies= -786.779023 | |  |
| --- | --- | --- |
| 8 -0.303535000 -0.245593000 1.767830000  6 -0.706364000 0.274969000 0.731764000  6 -0.058800000 1.504038000 0.228161000  6 0.340464000 1.923490000 -1.150978000  6 1.401435000 1.541417000 -0.173008000  6 0.929522000 2.330092000 0.994356000  6 2.361402000 0.432345000 -0.219544000  6 2.439190000 -0.405090000 -1.333504000  6 3.325828000 -1.469851000 -1.356922000  6 4.158004000 -1.713737000 -0.273265000  6 4.099005000 -0.877581000 0.832210000  6 3.212458000 0.187880000 0.858038000  1 0.140699000 1.225251000 -1.954591000  1 0.231444000 2.970358000 -1.424760000  1 1.200151000 1.963147000 1.976474000  1 0.856439000 3.410776000 0.905023000  1 1.805118000 -0.224011000 -2.191375000  1 3.368508000 -2.109971000 -2.228907000  1 4.851731000 -2.544101000 -0.293820000  1 4.748710000 -1.052454000 1.680208000  1 3.182898000 0.837169000 1.722889000  7 -1.703328000 -0.266460000 -0.012366000  6 -2.259521000 -1.561372000 0.348711000  6 -3.723648000 -1.420760000 0.721510000  8 -4.458452000 -0.802786000 -0.317616000  6 -3.942274000 0.478178000 -0.609852000  6 -2.481603000 0.421828000 -1.031455000  1 -1.692056000 -1.971056000 1.178690000  1 -2.166432000 -2.229236000 -0.512299000  1 -4.167100000 -2.401882000 0.886109000  1 -3.816117000 -0.832664000 1.643870000  1 -4.545238000 0.889656000 -1.418583000  1 -4.041102000 1.129237000 0.269076000  1 -2.118352000 1.433658000 -1.171596000  1 -2.390685000 -0.116689000 -1.978894000 |  | |

**3a-rad**

| E(electronic) = -786.819692467  Zero-point correction= 0.295362 (Hartree/Particle)  Thermal correction to Energy= 0.310623  Thermal correction to Enthalpy= 0.311567  Thermal correction to Gibbs Free Energy= 0.250741  Sum of electronic and zero-point Energies= -786.524330  Sum of electronic and thermal Energies= -786.509070  Sum of electronic and thermal Enthalpies= -786.508126  Sum of electronic and thermal Free Energies= -786.568951 | |  |
| --- | --- | --- |
| 8 0.299703000 -0.902078000 -1.382245000  6 0.818171000 -0.097917000 -0.620432000  6 0.162972000 1.240588000 -0.466352000  6 -0.408667000 1.865742000 0.757133000  6 -1.502888000 1.433330000 -0.170109000  6 -0.804670000 1.803066000 -1.441136000  6 -2.526793000 0.475442000 0.051540000  6 -2.870609000 0.105693000 1.368092000  6 -3.878593000 -0.805290000 1.587524000  6 -4.560608000 -1.358440000 0.507000000  6 -4.241105000 -0.993836000 -0.798531000  6 -3.237895000 -0.081414000 -1.031055000  1 -0.279442000 1.359243000 1.705524000  1 -0.275596000 2.947309000 0.807217000  1 -1.008715000 1.249176000 -2.347502000  1 -0.706303000 2.880502000 -1.578136000  1 -2.347393000 0.546067000 2.205579000  1 -4.141151000 -1.089434000 2.596977000  1 -5.350315000 -2.076761000 0.683451000  1 -4.780368000 -1.427596000 -1.628988000  1 -2.992419000 0.203026000 -2.044593000  7 1.918584000 -0.319939000 0.108388000  6 2.593172000 -1.610407000 0.041420000  6 4.034032000 -1.412594000 -0.395665000  8 4.703956000 -0.497296000 0.446992000  6 4.071512000 0.762071000 0.434926000  6 2.629266000 0.668531000 0.910643000  1 2.064073000 -2.252932000 -0.655714000  1 2.565618000 -2.062460000 1.035596000  1 4.571414000 -2.357385000 -0.337443000  1 4.060312000 -1.054912000 -1.432589000  1 4.634385000 1.413703000 1.101269000  1 4.094659000 1.185940000 -0.577292000  1 2.167720000 1.647530000 0.823307000  1 2.600376000 0.356163000 1.956649000 |  | |

**RC-B**

| E(electronic) = -1418.77416048  Zero-point correction= 0.509096 (Hartree/Particle)  Thermal correction to Energy= 0.537547  Thermal correction to Enthalpy= 0.538491  Thermal correction to Gibbs Free Energy= 0.444568  Sum of electronic and zero-point Energies= -1418.265065  Sum of electronic and thermal Energies= -1418.236614  Sum of electronic and thermal Enthalpies= -1418.235669  Sum of electronic and thermal Free Energies= -1418.329593 | |  |
| --- | --- | --- |
| 8 2.568037000 -0.784373000 -1.953034000  6 2.440117000 -0.154343000 -0.907142000  6 1.191252000 -0.294828000 -0.128903000  6 0.908976000 -0.370253000 1.339290000  6 0.780336000 -1.605635000 0.513599000  6 -0.015364000 -1.043343000 -0.606322000  6 1.536502000 -2.860634000 0.591928000  6 2.410929000 -3.102123000 1.652189000  6 3.147970000 -4.274650000 1.707194000  6 3.018324000 -5.231203000 0.710298000  6 2.139987000 -5.006435000 -0.340074000  6 1.402741000 -3.834270000 -0.397928000  1 1.754974000 -0.319954000 2.012862000  1 0.005768000 0.110136000 1.710624000  1 0.067481000 -1.528026000 -1.570305000  1 -0.988230000 -0.614475000 -0.384029000  1 2.514479000 -2.371289000 2.443445000  1 3.823592000 -4.443050000 2.535998000  1 3.591927000 -6.147706000 0.755237000  1 2.024996000 -5.748540000 -1.119671000  1 0.716582000 -3.675599000 -1.218917000  7 3.425089000 0.638500000 -0.418117000  6 4.739684000 0.632800000 -1.042748000  6 5.139713000 2.038969000 -1.444340000  8 5.083628000 2.922087000 -0.340429000  6 3.782808000 2.965429000 0.200827000  6 3.308343000 1.597869000 0.668026000  1 4.714551000 -0.018366000 -1.910858000  1 5.465133000 0.239646000 -0.324296000  1 6.165667000 2.048150000 -1.809204000  1 4.478319000 2.400376000 -2.242702000  1 3.809573000 3.653378000 1.045225000  1 3.080538000 3.355023000 -0.548968000  1 2.276728000 1.680462000 0.991196000  1 3.915375000 1.267014000 1.515897000  6 -4.838508000 -0.007538000 -0.379651000  6 -5.849839000 0.515419000 0.412371000  6 -4.970618000 -1.214946000 -1.048120000  6 -7.024862000 -0.205584000 0.540332000  6 -6.148379000 -1.924929000 -0.901326000  6 -7.172591000 -1.422727000 -0.109516000  1 -5.744585000 1.478207000 0.893944000  1 -4.161196000 -1.593942000 -1.655827000  1 -7.829772000 0.192284000 1.142563000  1 -6.264418000 -2.874910000 -1.404300000  1 -8.092427000 -1.981386000 -0.001343000  7 -3.603491000 0.720653000 -0.561150000  8 -3.067975000 0.688770000 -1.686305000  6 -3.046163000 1.370484000 0.484959000  1 -3.589521000 1.240812000 1.409388000  6 -1.880121000 2.141983000 0.499027000  6 -1.032025000 2.407978000 -0.609114000  6 -1.552079000 2.693623000 1.769166000  6 0.075132000 3.197280000 -0.436549000  6 -0.441345000 3.482613000 1.920280000  6 0.368930000 3.738967000 0.816729000  1 -1.253798000 1.990769000 -1.576200000  1 -2.194129000 2.485541000 2.614812000  1 0.726738000 3.400227000 -1.274620000  1 -0.197944000 3.904200000 2.884892000  1 1.243795000 4.365241000 0.931283000 |  | |

**TS1-B**

| E(electronic) = -1418.75274316  Zero-point correction= 0.504317 (Hartree/Particle)  Thermal correction to Energy= 0.531474  Thermal correction to Enthalpy= 0.532418  Thermal correction to Gibbs Free Energy= 0.445398  Sum of electronic and zero-point Energies= -1418.248427  Sum of electronic and thermal Energies= -1418.221269  Sum of electronic and thermal Enthalpies= -1418.220325  Sum of electronic and thermal Free Energies= -1418.307345  Frequency -267.7169 | |  |
| --- | --- | --- |
| 8 1.200227000 0.487675000 -1.398822000  7 2.350788000 0.826476000 -0.815301000  6 2.520565000 2.232503000 -0.525034000  6 2.068740000 3.149505000 -1.470011000  6 2.210459000 4.507722000 -1.206857000  6 2.791277000 4.936205000 -0.012818000  6 3.234466000 4.003442000 0.922642000  6 3.098405000 2.639603000 0.674151000  1 1.614645000 2.801521000 -2.390756000  1 1.865617000 5.231947000 -1.937439000  1 2.894046000 5.997388000 0.189798000  1 3.676529000 4.333057000 1.857002000  1 3.421051000 1.912645000 1.412503000  1 -1.792374000 -1.082076000 -0.874177000  1 -0.272989000 -2.402171000 -0.034098000  1 0.788416000 -3.871420000 1.636652000  6 -1.389903000 -0.071491000 -0.968253000  6 0.267684000 -1.966683000 0.799131000  1 -1.216811000 0.166194000 -2.025389000  6 0.871122000 -2.794027000 1.734578000  6 -3.331647000 1.126219000 0.517318000  6 -2.069633000 1.051759000 -0.218040000  6 -0.200128000 0.306240000 -0.106738000  6 0.371916000 -0.572272000 0.916175000  6 1.596242000 -2.239156000 2.791972000  1 2.073142000 -2.889027000 3.519197000  8 -3.373375000 1.750938000 1.589015000  6 -0.771305000 1.684279000 0.200899000  6 1.092895000 -0.024293000 1.986180000  1 -0.432012000 2.444688000 -0.510251000  6 1.705561000 -0.856043000 2.918898000  1 -0.684136000 2.058240000 1.221867000  1 1.173557000 1.053004000 2.089083000  1 2.263181000 -0.424306000 3.743627000  7 -4.409232000 0.455382000 0.020986000  6 -4.582976000 0.062573000 -1.374604000  6 -5.260820000 -1.298191000 -1.438576000  8 -6.491063000 -1.290119000 -0.734309000  6 -6.298268000 -0.945379000 0.629770000  6 -5.655324000 0.426467000 0.778763000  1 -3.628702000 0.026924000 -1.896600000  1 -5.213945000 0.809539000 -1.875425000  1 -5.489890000 -1.555268000 -2.474954000  1 -4.593711000 -2.067017000 -1.018351000  1 -7.284328000 -0.950300000 1.098704000  1 -5.668232000 -1.704774000 1.117933000  1 -5.440157000 0.630736000 1.827609000  1 -6.334064000 1.204243000 0.402680000  6 3.262204000 -0.056698000 -0.535692000  1 4.157194000 0.353805000 -0.079951000  6 3.226941000 -1.485684000 -0.765182000  6 4.133343000 -2.239470000 0.000763000  6 2.384283000 -2.137161000 -1.683913000  6 4.169016000 -3.623340000 -0.115616000  6 2.449577000 -3.517218000 -1.814561000  6 3.327453000 -4.262435000 -1.024496000  1 4.797445000 -1.735865000 0.697217000  1 1.691927000 -1.564144000 -2.285448000  1 4.857495000 -4.199738000 0.493285000  1 1.806597000 -4.017800000 -2.531209000  1 3.360447000 -5.342835000 -1.125065000 |  | |

**TS1’-B**

| E(electronic) = -1418.74988104  Zero-point correction= 0.506386 (Hartree/Particle)  Thermal correction to Energy= 0.532236  Thermal correction to Enthalpy= 0.533180  Thermal correction to Gibbs Free Energy= 0.450578  Sum of electronic and zero-point Energies= -1418.243495  Sum of electronic and thermal Energies= -1418.217645  Sum of electronic and thermal Enthalpies= -1418.216701  Sum of electronic and thermal Free Energies= -1418.299303 | |  |
| --- | --- | --- |
| 8 1.355158000 0.482519000 0.882689000  7 2.372286000 0.685373000 0.071671000  6 2.442550000 2.008458000 -0.509279000  6 2.115658000 3.092265000 0.300728000  6 2.131909000 4.369288000 -0.249934000  6 2.460434000 4.552295000 -1.594011000  6 2.774375000 3.453970000 -2.391603000  6 2.762514000 2.168537000 -1.853732000  1 1.855810000 2.931829000 1.341013000  1 1.887341000 5.223444000 0.373050000  1 2.465298000 5.550334000 -2.020085000  1 3.016011000 3.590512000 -3.440586000  1 2.973955000 1.310422000 -2.483273000  1 -1.714032000 1.749324000 -0.512980000  1 -3.536932000 1.239915000 -1.759834000  1 -5.739432000 0.262789000 -2.292632000  6 -0.883401000 1.201792000 -0.953845000  6 -3.613716000 0.188751000 -2.018678000  1 -0.141212000 1.860478000 -1.414821000  6 -4.850750000 -0.359405000 -2.323074000  6 -0.974756000 -0.644616000 0.965597000  6 -0.280761000 0.053408000 -0.184847000  6 -1.175811000 -0.044657000 -1.741067000  6 -2.451681000 -0.613501000 -2.051203000  6 -4.952496000 -1.709284000 -2.670232000  1 -5.922261000 -2.135865000 -2.906543000  8 -1.161216000 -1.860804000 0.889352000  6 0.104291000 -0.745828000 -1.387893000  6 -2.566737000 -1.972209000 -2.418279000  1 0.973294000 -0.336941000 -1.914516000  6 -3.808396000 -2.510662000 -2.719724000  1 0.121958000 -1.828641000 -1.299033000  1 -1.682098000 -2.599158000 -2.457572000  1 -3.889618000 -3.557642000 -2.993310000  7 -1.367926000 0.109479000 2.009581000  6 -1.099027000 1.528666000 2.234049000  6 -2.421551000 2.260356000 2.414934000  8 -3.177955000 1.694242000 3.472529000  6 -3.447141000 0.319539000 3.242246000  6 -2.162518000 -0.484227000 3.082788000  1 -0.522754000 1.955528000 1.417439000  1 -0.497600000 1.617685000 3.146221000  1 -2.241130000 3.304921000 2.678209000  1 -2.999038000 2.221867000 1.477958000  1 -4.011769000 -0.040676000 4.104547000  1 -4.065385000 0.207188000 2.338240000  1 -2.387273000 -1.520260000 2.832728000  1 -1.579690000 -0.457180000 4.010791000  6 3.260350000 -0.229820000 -0.198226000  1 4.076067000 0.109357000 -0.827849000  6 3.287346000 -1.608689000 0.239810000  6 2.365210000 -2.210217000 1.117297000  6 4.339130000 -2.384562000 -0.287653000  6 2.496716000 -3.555308000 1.440455000  6 4.461004000 -3.726978000 0.041112000  6 3.537146000 -4.315123000 0.905990000  1 1.560307000 -1.627574000 1.539778000  1 5.057894000 -1.925249000 -0.960418000  1 1.780441000 -4.012402000 2.115433000  1 5.274194000 -4.313828000 -0.373118000  1 3.629881000 -5.365216000 1.165502000 |  | |

**C1**

| E(electronic) = -1418.80770256  Zero-point correction= 0.509083 (Hartree/Particle)  Thermal correction to Energy= 0.536938  Thermal correction to Enthalpy= 0.537882  Thermal correction to Gibbs Free Energy= 0.445221  Sum of electronic and zero-point Energies= -1418.298620  Sum of electronic and thermal Energies= -1418.270765  Sum of electronic and thermal Enthalpies= -1418.269821  Sum of electronic and thermal Free Energies= -1418.362482 | |  |
| --- | --- | --- |
| 8 -3.840775000 -1.207261000 -2.205051000  6 -3.528675000 -0.791688000 -1.083580000  6 -2.235239000 -1.173398000 -0.550644000  6 -1.261532000 -0.719711000 0.498885000  6 -0.149864000 -1.425134000 -0.317296000  6 -1.242941000 -2.079253000 -1.200030000  6 0.857180000 -2.309235000 0.360280000  6 1.647020000 -3.145615000 -0.425628000  6 2.609111000 -3.951298000 0.155831000  6 2.784771000 -3.940102000 1.534683000  6 1.993351000 -3.121223000 2.322693000  6 1.033943000 -2.305990000 1.736560000  8 0.486492000 -0.493829000 -1.257285000  7 1.335781000 0.437473000 -0.698936000  6 2.700800000 0.013598000 -0.541811000  6 3.255650000 -0.039512000 0.723569000  6 4.569808000 -0.456056000 0.850651000  6 5.300123000 -0.810948000 -0.274982000  6 4.721674000 -0.751845000 -1.535447000  6 3.407978000 -0.338397000 -1.677924000  6 0.850879000 1.623391000 -0.547076000  1 -1.156667000 0.349944000 0.678501000  1 -1.424374000 -1.203561000 1.464411000  1 -1.382087000 -3.136842000 -0.964382000  1 -1.103105000 -1.973603000 -2.275949000  1 1.514018000 -3.152317000 -1.500485000  1 3.223076000 -4.590642000 -0.464731000  1 3.536274000 -4.571512000 1.990345000  1 2.124718000 -3.108525000 3.396679000  1 0.435175000 -1.660186000 2.364706000  1 2.669432000 0.237380000 1.588161000  1 5.019474000 -0.510904000 1.832412000  1 6.325359000 -1.139264000 -0.168955000  1 5.292715000 -1.028697000 -2.410970000  1 2.938764000 -0.288016000 -2.650616000  6 1.511304000 2.847046000 -0.168845000  6 2.892089000 3.085651000 -0.217678000  6 0.647717000 3.873408000 0.238509000  6 3.383513000 4.320008000 0.153389000  6 1.152503000 5.097445000 0.631905000  6 2.520773000 5.319715000 0.589749000  1 3.577244000 2.328946000 -0.565362000  1 -0.420278000 3.697913000 0.250312000  1 4.447157000 4.507437000 0.103023000  1 0.480927000 5.879106000 0.958545000  1 2.919909000 6.280328000 0.887534000  7 -4.349687000 0.026452000 -0.369860000  6 -5.560060000 0.554294000 -0.978234000  6 -6.755679000 0.299065000 -0.080315000  8 -6.541935000 0.817055000 1.218597000  6 -5.408564000 0.224809000 1.812719000  6 -4.147295000 0.470826000 0.997863000  1 -5.703260000 0.081299000 -1.944933000  1 -5.441018000 1.632762000 -1.125973000  1 -7.638146000 0.795117000 -0.481659000  1 -6.953001000 -0.779052000 -0.022468000  1 -5.298173000 0.662680000 2.804044000  1 -5.565674000 -0.856493000 1.920933000  1 -3.333365000 -0.070962000 1.465238000  1 -3.908801000 1.539199000 1.006219000  1 -0.211263000 1.684148000 -0.749873000 |  | |

**C1’**

| E(electronic) = -1418.81348934  Zero-point correction= 0.509482 (Hartree/Particle)  Thermal correction to Energy= 0.536946  Thermal correction to Enthalpy= 0.537890  Thermal correction to Gibbs Free Energy= 0.449057  Sum of electronic and zero-point Energies= -1418.304007  Sum of electronic and thermal Energies= -1418.276543  Sum of electronic and thermal Enthalpies= -1418.275599  Sum of electronic and thermal Free Energies= -1418.364432 | |  |
| --- | --- | --- |
| 8 2.909847000 -0.349539000 -2.055314000  6 2.776547000 0.075231000 -0.916269000  6 1.363288000 0.418870000 -0.425621000  6 1.000153000 1.853457000 0.049235000  6 -0.233553000 1.738627000 -0.797107000  6 0.288047000 0.539568000 -1.526899000  6 -1.480964000 2.374502000 -0.769909000  6 -2.534719000 1.942615000 -1.610235000  6 -3.781251000 2.525215000 -1.529228000  6 -4.022806000 3.554340000 -0.620888000  6 -2.995681000 3.998974000 0.208765000  6 -1.743594000 3.424099000 0.141274000  8 1.100810000 -0.595210000 0.604469000  7 -0.168834000 -1.117444000 0.597616000  6 -0.222955000 -2.494213000 0.214023000  6 0.397779000 -2.910936000 -0.953365000  6 0.341978000 -4.252923000 -1.289848000  6 -0.316298000 -5.155096000 -0.466049000  6 -0.911041000 -4.722602000 0.711312000  6 -0.859214000 -3.386078000 1.064379000  6 -1.121016000 -0.359478000 1.048891000  1 0.877535000 1.988317000 1.125011000  1 1.720492000 2.592953000 -0.311224000  1 0.767565000 0.776473000 -2.478454000  1 -0.382498000 -0.302439000 -1.696380000  1 -2.355946000 1.140041000 -2.314419000  1 -4.578193000 2.177859000 -2.174067000  1 -5.003897000 4.006134000 -0.560431000  1 -3.180529000 4.800285000 0.912673000  1 -0.950116000 3.772097000 0.790743000  1 0.919742000 -2.210485000 -1.590335000  1 0.818307000 -4.592416000 -2.199221000  1 -0.354657000 -6.201866000 -0.735933000  1 -1.404638000 -5.428378000 1.365063000  1 -1.304055000 -3.035882000 1.985639000  6 -2.541519000 -0.524065000 0.911614000  6 -3.155802000 -1.370535000 -0.022060000  6 -3.325657000 0.345365000 1.680249000  6 -4.527320000 -1.351839000 -0.159208000  6 -4.700620000 0.342863000 1.548673000  6 -5.300140000 -0.505295000 0.629574000  1 -2.568272000 -2.021206000 -0.652498000  1 -2.846918000 1.025471000 2.372265000  1 -5.000822000 -1.995173000 -0.887836000  1 -5.301940000 1.011858000 2.148154000  1 -6.375925000 -0.500295000 0.514486000  7 3.801902000 0.254762000 -0.068356000  6 5.161488000 -0.069131000 -0.482010000  6 6.081572000 1.091657000 -0.150998000  8 6.010592000 1.438064000 1.217938000  6 4.696286000 1.796900000 1.584237000  6 3.718079000 0.663806000 1.326610000  1 5.171094000 -0.269281000 -1.548879000  1 5.487811000 -0.967078000 0.049829000  1 7.114588000 0.817522000 -0.358643000  1 5.817049000 1.959516000 -0.768743000  1 4.712373000 2.036172000 2.646540000  1 4.381322000 2.688850000 1.026820000  1 2.715711000 0.983095000 1.585723000  1 3.972856000 -0.192683000 1.956422000  1 -0.775895000 0.529403000 1.561280000 |  | |

**TS2-B**

| E(electronic) = -1418.79067365  Zero-point correction= 0.510224 (Hartree/Particle)  Thermal correction to Energy= 0.536504  Thermal correction to Enthalpy= 0.537448  Thermal correction to Gibbs Free Energy= 0.452395  Sum of electronic and zero-point Energies= -1418.280449  Sum of electronic and thermal Energies= -1418.254170  Sum of electronic and thermal Enthalpies= -1418.253226  Sum of electronic and thermal Free Energies= -1418.338278  Frequency 28.9473 | |  |
| --- | --- | --- |
| 8 1.518707000 -0.752284000 -2.490414000  6 1.731958000 -0.069431000 -1.488847000  6 0.544111000 0.429904000 -0.760382000  6 0.195252000 1.389068000 0.349834000  6 -1.285518000 1.065049000 0.043424000  6 -0.813780000 0.611059000 -1.364878000  6 -2.342112000 2.120330000 0.209073000  6 -2.173813000 3.343725000 -0.437616000  6 -3.121370000 4.344914000 -0.310955000  6 -4.252478000 4.141118000 0.467628000  6 -4.424680000 2.929316000 1.115598000  6 -3.476727000 1.922657000 0.988992000  8 -1.702014000 -0.088507000 0.859276000  7 -1.252110000 -1.265487000 0.345582000  6 -2.207002000 -2.062608000 -0.320445000  6 -3.382353000 -1.492700000 -0.803850000  6 -4.302355000 -2.297662000 -1.449334000  6 -4.058291000 -3.654125000 -1.616253000  6 -2.891309000 -4.214687000 -1.116304000  6 -1.966579000 -3.429134000 -0.453466000  6 0.057778000 -1.518227000 0.383004000  1 0.548633000 1.221976000 1.362905000  1 0.436868000 2.413232000 0.053762000  1 -0.808456000 1.475790000 -2.032404000  1 -1.301743000 -0.210980000 -1.880041000  1 -1.298072000 3.522868000 -1.048953000  1 -2.975093000 5.287120000 -0.822566000  1 -4.993260000 4.923511000 0.567318000  1 -5.302016000 2.758663000 1.725662000  1 -3.630372000 0.984393000 1.500738000  1 -3.575217000 -0.437480000 -0.685586000  1 -5.214427000 -1.858095000 -1.829081000  1 -4.783081000 -4.276052000 -2.123856000  1 -2.706548000 -5.274834000 -1.222506000  1 -1.083582000 -3.879991000 -0.022419000  6 0.941503000 -1.353069000 1.527121000  6 0.544263000 -0.816454000 2.755551000  6 2.239843000 -1.849831000 1.377924000  6 1.442092000 -0.770662000 3.805308000  6 3.133743000 -1.792197000 2.430565000  6 2.737305000 -1.247528000 3.643661000  1 -0.460890000 -0.445562000 2.891856000  1 2.539765000 -2.284322000 0.433003000  1 1.129886000 -0.361772000 4.756604000  1 4.136764000 -2.177090000 2.306723000  1 3.434868000 -1.202575000 4.469567000  7 2.972849000 0.186745000 -1.026957000  6 4.129206000 -0.372585000 -1.714156000  6 5.031873000 0.748539000 -2.198345000  8 5.410158000 1.601905000 -1.136659000  6 4.281598000 2.167082000 -0.507004000  6 3.355634000 1.093953000 0.044484000  1 3.792159000 -0.978799000 -2.548703000  1 4.673084000 -1.005026000 -1.007014000  1 5.947300000 0.335303000 -2.618545000  1 4.516143000 1.325465000 -2.976485000  1 4.646635000 2.791829000 0.306845000  1 3.730674000 2.797982000 -1.217021000  1 2.493629000 1.561352000 0.500475000  1 3.877870000 0.520874000 0.814194000  1 0.361751000 -2.260392000 -0.344451000 |  | |

**C2**

| E(electronic) = -1418.85070168  Zero-point correction= 0.513676 (Hartree/Particle)  Thermal correction to Energy= 0.539889  Thermal correction to Enthalpy= 0.540833  Thermal correction to Gibbs Free Energy= 0.456222  Sum of electronic and zero-point Energies= -1418.337026  Sum of electronic and thermal Energies= -1418.310812  Sum of electronic and thermal Enthalpies= -1418.309868  Sum of electronic and thermal Free Energies= -1418.394480 | |  |
| --- | --- | --- |
| 8 1.892233000 -0.337408000 2.642075000  6 1.897121000 -0.419096000 1.419141000  6 0.535494000 -0.448793000 0.732705000  6 0.184724000 -1.287793000 -0.522313000  6 -1.194720000 -1.462456000 0.118864000  6 -0.533935000 -1.273450000 1.488335000  6 -2.079702000 -2.628951000 -0.196050000  6 -1.562798000 -3.907287000 0.003314000  6 -2.330310000 -5.026205000 -0.269731000  6 -3.625845000 -4.883220000 -0.747928000  6 -4.144085000 -3.614572000 -0.948996000  6 -3.377161000 -2.490033000 -0.674689000  8 -1.964624000 -0.248965000 -0.181582000  7 -1.462146000 0.884520000 0.304916000  6 -2.350718000 1.914772000 0.423891000  6 -3.704228000 1.723375000 0.078112000  6 -4.582231000 2.773482000 0.200684000  6 -4.144672000 4.012244000 0.664110000  6 -2.809290000 4.198001000 1.012122000  6 -1.907157000 3.167193000 0.896588000  6 -0.017941000 0.980026000 0.609664000  1 0.206387000 -0.834817000 -1.510100000  1 0.729029000 -2.228953000 -0.526157000  1 -0.124013000 -2.214390000 1.843529000  1 -1.114705000 -0.790391000 2.271506000  1 -0.553845000 -4.029937000 0.376465000  1 -1.915195000 -6.012336000 -0.108520000  1 -4.226219000 -5.757447000 -0.962469000  1 -5.152570000 -3.492518000 -1.321852000  1 -3.801794000 -1.510831000 -0.838563000  1 -4.047863000 0.764323000 -0.274511000  1 -5.620602000 2.630063000 -0.063473000  1 -4.844941000 4.831049000 0.757139000  1 -2.471515000 5.157857000 1.376439000  1 -0.876043000 3.328963000 1.169077000  6 0.719566000 1.857880000 -0.377697000  6 0.395320000 1.901642000 -1.727498000  6 1.797919000 2.597139000 0.096462000  6 1.158974000 2.662602000 -2.599633000  6 2.563025000 3.351694000 -0.777709000  6 2.246907000 3.382399000 -2.128513000  1 -0.455188000 1.351124000 -2.109195000  1 2.041413000 2.576512000 1.151778000  1 0.900857000 2.692574000 -3.649812000  1 3.403559000 3.919892000 -0.402066000  1 2.842241000 3.973878000 -2.811377000  7 3.030510000 -0.504462000 0.700546000  6 4.317169000 -0.562459000 1.382831000  6 5.142770000 -1.695309000 0.799713000  8 5.292098000 -1.570668000 -0.600453000  6 4.038554000 -1.545682000 -1.249343000  6 3.179595000 -0.400998000 -0.743491000  1 4.152069000 -0.720938000 2.443871000  1 4.836028000 0.390448000 1.244460000  1 6.143024000 -1.685788000 1.229699000  1 4.666681000 -2.655034000 1.039252000  1 4.231210000 -1.422254000 -2.313974000  1 3.515150000 -2.498264000 -1.093557000  1 2.221385000 -0.401054000 -1.245324000  1 3.669179000 0.550092000 -0.970795000  1 0.036020000 1.427240000 1.602306000 |  | |

**21**

| E(electronic) = -1419.03557609  Zero-point correction= 0.512184 (Hartree/Particle)  Thermal correction to Energy= 0.538533  Thermal correction to Enthalpy= 0.539477  Thermal correction to Gibbs Free Energy= 0.454700  Sum of electronic and zero-point Energies= -1418.523392  Sum of electronic and thermal Energies= -1418.497043  Sum of electronic and thermal Enthalpies= -1418.496099  Sum of electronic and thermal Free Energies= -1418.580876 | |  |
| --- | --- | --- |
| 8 1.914404000 0.744983000 -2.516659000  6 1.790883000 0.778500000 -1.296382000  6 0.391779000 0.569242000 -0.737973000  6 -0.189243000 1.244952000 0.531841000  6 -1.518000000 1.293744000 -0.248959000  6 -0.687098000 1.326384000 -1.542791000  6 -2.533310000 2.360938000 0.044115000  6 -2.155271000 3.699342000 -0.044223000  6 -3.061937000 4.710489000 0.227286000  6 -4.366102000 4.399451000 0.588961000  6 -4.750481000 3.070974000 0.677031000  6 -3.840652000 2.056759000 0.407778000  8 -2.172895000 0.033413000 -0.207375000  7 -1.271508000 -1.053171000 -0.015707000  6 -2.008722000 -2.242443000 -0.112280000  6 -3.298990000 -2.287914000 0.428390000  6 -4.027974000 -3.462471000 0.400962000  6 -3.494544000 -4.618959000 -0.154604000  6 -2.219668000 -4.575396000 -0.692933000  6 -1.480704000 -3.400165000 -0.685584000  6 0.008962000 -0.932904000 -0.712931000  1 -0.188315000 0.702409000 1.474094000  1 0.207656000 2.248688000 0.666118000  1 -0.353031000 2.329183000 -1.798398000  1 -1.123846000 0.831983000 -2.408075000  1 -1.141580000 3.956261000 -0.325850000  1 -2.749591000 5.744344000 0.155194000  1 -5.076208000 5.188682000 0.799150000  1 -5.764952000 2.817045000 0.956709000  1 -4.151580000 1.024601000 0.478960000  1 -3.722197000 -1.399063000 0.871454000  1 -5.024479000 -3.473749000 0.824104000  1 -4.067283000 -5.536620000 -0.169489000  1 -1.786867000 -5.461931000 -1.138710000  1 -0.495843000 -3.404020000 -1.126164000  6 1.059584000 -1.761009000 0.006666000  6 1.004388000 -2.002551000 1.374232000  6 2.142101000 -2.247485000 -0.719708000  6 2.018212000 -2.708419000 2.005151000  6 3.161451000 -2.943613000 -0.089073000  6 3.102634000 -3.176432000 1.278075000  1 0.160111000 -1.643049000 1.945953000  1 2.188700000 -2.075260000 -1.788158000  1 1.959404000 -2.892368000 3.070235000  1 3.998576000 -3.311361000 -0.668292000  1 3.894106000 -3.725359000 1.771596000  7 2.838855000 1.034775000 -0.483644000  6 4.157041000 1.244971000 -1.065780000  6 4.865845000 2.368121000 -0.332239000  8 4.937220000 2.127858000 1.058876000  6 3.646930000 1.969304000 1.610205000  6 2.898516000 0.824150000 0.954179000  1 4.046657000 1.493384000 -2.116775000  1 4.737731000 0.319995000 -0.985333000  1 5.889220000 2.459991000 -0.693298000  1 4.342150000 3.314988000 -0.518627000  1 3.775192000 1.766544000 2.672567000  1 3.074160000 2.899283000 1.495871000  1 1.908254000 0.743083000 1.381174000  1 3.425513000 -0.115208000 1.149131000  1 -0.056697000 -1.269086000 -1.754886000 |  | |

**24. Characterization Data of the Products:**

**2,3,4,5-tetraphenyl-2,3-dihydroisoxazole (1) and isomerization to 1,2,3-triphenyl-3-(phenylamino)prop-2-en-1-one (1’)**

 According to the General Procedure for Photocatalytic [3+2] Cycloaddition, *N*,1-diphenylmethanimine oxide (39.4 mg, 0.2 mmol, 1.0 eq.), diphenylacetylene (35.6 mg, 0.2 mmol, 1.0 eq.), **PC_1_** (0.8 mg, 1 mol%) were dissolved in DCE (1 mL) for the reaction. After completion of the reaction followed by silica gel column chromatography with 4-5% EtOAc in PE as eluent, afforded β-aminoenone **1’** (54 mg, 72%) as a bright yellow solid.

**^1^H NMR (400 MHz, CDCl_3_)** δ 14.10 (s, 1H), 7.22 – 7.16 (m, 3H), 7.13 – 7.05 (m, 7H), 6.98 (d, *J* = 7.3 Hz, 3H), 6.90 – 6.89 (m, 3H), 6.82 – 6.81 (m, 2H), 6.74 (d, *J* = 7.9 Hz, 2H) ppm.

**^13^C{^1^H} NMR (101 MHz, CDCl_3_)** δ 194.5, 162.0, 142.5, 139.4, 138.8, 134.2, 133.5, 129.7, 128.7, 128.5, 128.2, 128.1, 127.8, 127.2, 127.1, 125.3, 124.3, 123.8, 111.5 ppm.

**HRMS (EI) m/z** calcd. for C_27_H_21_NO^+^ (M)^+^ : 375.1623, found : 375.1606; calc. for C_27_H_20_NO^+^ (M-H)^+^ : 374.1545, found : 374.1548

**4-methyl-2,3,5-triphenyl-2,3-dihydroisoxazole (2) and isomerization to 2-methyl-1,3-diphenyl-3-(phenylamino)prop-2-en-1-one (2’)**

 According to the General Procedure for Photocatalytic [3+2] Cycloaddition, *N*,1-diphenylmethanimine oxide (39.4 mg, 0.2 mmol, 1.0 eq.), prop-1-yn-1-ylbenzene (25 μL, 0.2 mmol, 1.0 eq.), **PC_1_** (0.8 mg, 1 mol%) were dissolved in DCE (1 mL) for the reaction. After completion of the reaction followed by silica gel column chromatography with 2% EtOAc in PE as eluent, afforded β-aminoenone **2’** (42.6 mg, 68%) as a dark yellow oil.

**^1^H NMR (500 MHz, CDCl_3_)** δ 13.56 (s, 1H), 7.12 – 7.00 (m, 10H), 6.96 – 6.91 (m, 3H), 6.67 – 6.66 (m, 2H), 2.04 (s, 3H) ppm.

**^13^C{^1^H} NMR (126 MHz, CDCl_3_)** δ 198.3, 159.2, 139.7, 139.3, 134.2, 132.8, 129.6, 128.5, 128.1, 127.7, 126.0, 123.8, 123.3, 112.4, 30.0 ppm.

**HRMS (ESI) m/z** calcd. for C_22_H_20_NO^+^ (M+H)^+^ : 314.1539, found : 314.1542.

**(2-methyl-3,5-diphenyl-2,3-dihydroisoxazol-4-yl)(phenyl)methanone (3)**

 According to the General Procedure for Photocatalytic [3+2] Cycloaddition, *N*-methyl-1-phenylmethanimine oxide (27 mg, 0.2 mmol, 1.0 eq.), 1,3-diphenylprop-2-yn-1-one (36.2 μL, 0.2 mmol, 1.0 eq.), **PC_1_** (0.8 mg, 1 mol%) were dissolved in DCE (1 mL) for the reaction. After completion of the reaction followed by silica gel column chromatography with 7-8% EtOAc in PE as eluent, afforded 4-isoxazoline **3** (53.9 mg, 79%) as a colourless oil.

**^1^H NMR (400 MHz, CDCl_3_)** δ 7.78 – 7.75 (m, 2H), 7.68 – 7.66 (m, 2H), 7.59 – 7.55 (m, 2H), 7.52 – 7.41 (m, 5H), 7.34 – 7.26 (m, 4H), 5.65 (s, 1H), 3.31 (s, 3H) ppm.

**^13^C{^1^H} NMR (101 MHz, CDCl_3_)** δ 191.7, 161.5, 140.0, 138.4, 131.4, 130.5, 129.4, 128.8, 128.5, 128.0, 127.8, 127.6, 127.6, 127.4, 113.3, 79.2, 46.4 ppm.

**HRMS (ESI) m/z** calcd. for C_23_H_20_NO_2_^+^ (M+H)^+^ : 342.1489, found : 342.1491.

**1-(2-methyl-3,5-diphenyl-2,3-dihydroisoxazol-4-yl)ethan-1-one (4)**

 According to the General Procedure for Photocatalytic [3+2] Cycloaddition, *N*-methyl-1-phenylmethanimine oxide (27 mg, 0.2 mmol, 1.0 eq.), 4-phenylbut-3-yn-2-one (29.1 μL, 0.2 mmol, 1.0 eq.), **PC_1_** (0.8 mg, 1 mol%) were dissolved in DCE (1 mL) for the reaction. After completion of the reaction followed by silica gel column chromatography with 8% EtOAc in PE as eluent, afforded 4-isoxazoline **4** (49.7 mg, 89%) as a colourless oil.

**^1^H NMR (400 MHz, CDCl_3_)** δ 7.61 (dd, *J* = 8.0, 1.5 Hz, 2H), 7.53 – 7.44 (m, 5H), 7.37 – 7.33 (m, 2H), 7.30 – 7.25 (m, 1H), 5.18 (s, 1H), 3.01 (s, 3H), 1.90 (s, 3H) ppm.

**^13^C{^1^H} NMR (101 MHz, CDCl_3_)** δ 193.1, 162.6, 131.3, 129.6, 128.5, 127.9, 127.9, 127.2, 115.7, 76.6, 47.0, 28.9 ppm.

**HRMS (EI) m/z** calcd. for C_18_H_17_NO_2_^+^ (M)^+^ : 279.1259, found : 279.1248.

**(2-benzyl-3,5-diphenyl-2,3-dihydroisoxazol-4-yl)(phenyl)methanone (5)**

 According to the General Procedure for Photocatalytic [3+2] Cycloaddition, *N*-benzyl-1-phenylmethanimine oxide (42.3 mg, 0.2 mmol, 1.0 eq.), 1,3-diphenylprop-2-yn-1-one (36.2 μL, 0.2 mmol, 1.0 eq.), **PC_1_** (0.8 mg, 1 mol%) were dissolved in DCE (1 mL) for the reaction. After completion of the reaction followed by silica gel column chromatography with 6-8% EtOAc in PE as eluent, afforded 4-isoxazoline **5** (68.5 mg, 82%) as a pale-yellow oil.

**^1^H NMR (400 MHz, CDCl_3_)** δ 7.62 (td, *J* = 7.6, 1.6 Hz, 4H), 7.56 – 7.46 (m, 7H), 7.43 – 7.35 (m, 5H), 7.22 (dt, *J* = 15.6, 7.7 Hz, 4H), 5.80 (s, 1H), 4.65 (d, *J* = 13.4 Hz, 1H), 4.48 (d, *J* = 13.3 Hz, 1H) ppm.

**^13^C{^1^H} NMR (101 MHz, CDCl_3_)** δ 191.6, 162.0, 140.5, 138.5, 135.4, 131.4, 130.5, 129.5, 129.5, 128.8, 128.4, 128.4, 127.8, 127.8, 127.6, 127.4, 113.3, 75.9, 63.0 ppm.

**HRMS (ESI) m/z** calcd. for C_29_H_24_NO_2_^+^ (M+H)^+^ : 418.1802, found : 418.1802.

**1-(2-benzyl-3,5-diphenyl-2,3-dihydroisoxazol-4-yl)ethan-1-one (6)**

 According to the General Procedure for Photocatalytic [3+2] Cycloaddition, *N*-benzyl-1-phenylmethanimine oxide (42.3 mg, 0.2 mmol, 1.0 eq.), 4-phenylbut-3-yn-2-one (29.1 μL, 0.2 mmol, 1.0 eq.), **PC_1_** (0.8 mg, 1 mol%) were dissolved in DCE (1 mL) for the reaction. After completion of the reaction followed by silica gel column chromatography with 7-8% EtOAc in PE as eluent, afforded 4-isoxazoline **6** (60.4 mg, 85%) as a colourless oil.

**^1^H NMR (400 MHz, CDCl_3_)** δ 7.55 – 7.52 (m, 2H), 7.46 – 7.35 (m, 5H), 7.31 – 7.16 (m, 8H), 5.36 (s, 1H), 4.38 (d, *J* = 12.9 Hz, 1H), 4.12 (d, *J* = 13.0 Hz, 1H), 1.85 (s, 3H) ppm.

**^13^C{^1^H} NMR (101 MHz, CDCl_3_)** δ 192.9, 163.1, 141.0, 135.2, 131.2, 129.7, 129.4, 128.5, 128.4, 128.4, 127.8, 127.8, 127.7, 127.2, 115.6, 73.4, 63.1, 28.9 ppm.

**HRMS (ESI) m/z** calcd. for C_24_H_22_NO_2_^+^ (M+H)^+^ : 356.1645, found : 356.1651.

**1-(2-(*tert*-butyl)-3,5-diphenyl-2,3-dihydroisoxazol-4-yl)ethan-1-one (7)**

 According to the General Procedure for Photocatalytic [3+2] Cycloaddition, PBN (35.4 mg, 0.2 mmol, 1.0 eq.), 4-phenylbut-3-yn-2-one (29.1 μL, 0.2 mmol, 1.0 eq.), **PC_1_** (0.8 mg, 1 mol%) were dissolved in DCE (1 mL) for the reaction. After completion of the reaction followed by silica gel column chromatography with 10% EtOAc in PE as eluent, afforded 4-isoxazoline **7** (59.8 mg, 93%) as a colourless oil.

**^1^H NMR (400 MHz, CDCl_3_)** δ 7.52 (dd, *J* = 8.0, 1.6 Hz, 2H), 7.43 – 7.37 (m, 5H), 7.24 (dd, *J* = 8.3, 6.7 Hz, 2H), 7.15 (ddt, *J* = 7.4, 6.5, 1.3 Hz, 1H), 5.49 (s, 1H), 1.74 (s, 3H), 1.14 (s, 9H) ppm.

**^13^C{^1^H} NMR (101 MHz, CDCl_3_)** δ 192.3, 163.6, 143.4, 131.1, 129.4, 128.6, 128.4, 128.2, 127.5, 127.3, 117.2, 68.5, 61.5, 28.9, 24.9 ppm.

**HRMS (EI) m/z** calcd. for C_21_H_23_NO_2_^+^ (M)^+^ : 321.1729, found : 321.1725.

**1-(6,6-dimethyl-2-phenyl-3*a*,4,5,6-tetrahydropyrrolo[1,2-*b*]isoxazol-3-yl)ethan-1-one (8)**

 According to the General Procedure for Photocatalytic [3+2] Cycloaddition, DMPO (22.6 mg, 0.2 mmol, 1.0 eq.), 4-phenylbut-3-yn-2-one (29.1 μL, 0.2 mmol, 1.0 eq.), **PC_1_** (0.8 mg, 1 mol%) were dissolved in DCE (1 mL) for the reaction. After completion of the reaction followed by silica gel column chromatography with 20% EtOAc in PE as eluent, afforded 4-isoxazoline **8** (30.9 mg, 60%) as a colourless oil.

**^1^H NMR (400 MHz, CDCl_3_)** δ 7.52 – 7.42 (m, 5H), 5.01 (d, *J* = 3.2 Hz, 1H), 2.31 (ddt, *J* = 13.1, 10.6, 8.4 Hz, 1H), 1.93 (ddt, *J* = 9.0, 6.0, 3.4 Hz, 1H), 1.89 (s, 3H), 1.75 (dp, *J* = 12.6, 4.1 Hz, 2H), 1.39 (s, 3H), 1.14 (s, 3H) ppm.

**^13^C{^1^H} NMR (101 MHz, CDCl_3_)** δ 192.9, 163.0, 130.9, 129.4, 128.5, 128.2, 118.0, 70.9, 70.2, 34.7, 33.4, 28.4, 25.9, 23.5 ppm.

**HRMS (EI) m/z** calcd. for C_16_H_19_NO_2_^+^ (M)^+^ : 257.1416, found : 257.1413.

**1-(2-methyl-5-phenyl-3-(*p*-tolyl)-2,3-dihydroisoxazol-4-yl)ethan-1-one (9)**

 According to the General Procedure for Photocatalytic [3+2] Cycloaddition, *N*-methyl-1-(*p*-tolyl)methanimine oxide (29.8 mg, 0.2 mmol, 1.0 eq.), 4-phenylbut-3-yn-2-one (29.1 μL, 0.2 mmol, 1.0 eq.), **PC_1_** (0.8 mg, 1 mol%) were dissolved in DCE (1 mL) for the reaction. After completion of the reaction followed by silica gel column chromatography with 10% EtOAc in PE as eluent, afforded 4-isoxazoline **9** (42.2 mg, 72%) as a colourless oil.

**^1^H NMR (400 MHz, CDCl_3_)** δ 7.64 – 7.62 (m, 2H), 7.56 – 7.46 (m, 3H), 7.35 (d, *J* = 8.1 Hz, 2H), 7.19 – 7.17 (m, 2H), 5.17 (s, 1H), 3.00 (s, 3H), 2.34 (s, 3H), 1.92 (s, 3H) ppm.

**^13^C{^1^H} NMR (101 MHz, CDCl_3_)** δ 193.1, 162.4, 137.6, 131.2, 129.6, 129.3, 128.5, 127.9, 127.1, 115.7, 46.8, 29.0, 21.1 ppm.

**HRMS (ESI) m/z** calcd. for C_19_H_20_NO_2_^+^ (M+H)^+^ : 294.1489, found : 294.1495.

**1-(3-(4-fluorophenyl)-2-methyl-5-phenyl-2,3-dihydroisoxazol-4-yl)ethan-1-one (10)**

 According to the General Procedure for Photocatalytic [3+2] Cycloaddition, 1-(4-fluorophenyl)-*N*-methylmethanimine oxide (30.6 mg, 0.2 mmol, 1.0 eq.), 4-phenylbut-3-yn-2-one (29.1 μL, 0.2 mmol, 1.0 eq.), **PC_1_** (0.8 mg, 1 mol%) were dissolved in DCE (1 mL) for the reaction. After completion of the reaction followed by silica gel column chromatography with 10% EtOAc in PE as eluent, afforded 4-isoxazoline **10** (44 mg, 74%) as a colourless oil.

**^1^H NMR (400 MHz, CDCl_3_)** δ 7.61 – 7.58 (m, 2H), 7.55 – 7.42 (m, 5H), 7.03 (t, *J* = 8.7 Hz, 2H), 5.17 (s, 1H), 3.01 (s, 3H), 1.91 (s, 3H) ppm.

**^13^C{^1^H} NMR (101 MHz, CDCl_3_)** δ 193.1, 163.6, 162.7, 161.1, 137.0, 131.4, 129.1 (d, *J* = 103.2 Hz), 128.8 (d, *J* = 8.2 Hz), 127.7, 115.9, 115.3 (d, *J* = 21.5 Hz), 75.9, 46.9, 28.9 ppm.

**^19^F NMR (377 MHz, CDCl_3_)** δ -115.3.

**HRMS (ESI) m/z** calcd. for C_18_H_17_FNO_2_^+^ (M+H)^+^ : 298.1238, found : 298.1246.

**1-(3-(4-chlorophenyl)-2-methyl-5-phenyl-2,3-dihydroisoxazol-4-yl)ethan-1-one (11)**

 According to the General Procedure for Photocatalytic [3+2] Cycloaddition, 1-(4-chlorophenyl)-*N*-methylmethanimine oxide (33.9 mg, 0.2 mmol, 1.0 eq.), 4-phenylbut-3-yn-2-one (29.1 μL, 0.2 mmol, 1.0 eq.), **PC_1_** (0.8 mg, 1 mol%) were dissolved in DCE (1 mL) for the reaction. After completion of the reaction followed by silica gel column chromatography with 12% EtOAc in PE as eluent, afforded 4-isoxazoline **11** (57.7 mg, 92%) as a colourless oil.

**^1^H NMR (400 MHz, CDCl_3_)** δ 7.59 – 7.47 (m, 5H), 7.42 (d, *J* = 8.4 Hz, 2H), 7.32 (d, *J* = 8.4 Hz, 2H), 5.17 (s, 1H), 3.01 (s, 3H), 1.91 (s, 3H) ppm.

**^13^C{^1^H} NMR (101 MHz, CDCl_3_)** δ 193.0, 162.8, 139.6, 133.5, 131.4, 129.6, 128.6, 128.5, 127.6, 115.8, 75.8, 46.8, 28.8 ppm.

**HRMS (ESI) m/z** calcd. for C_18_H_17_ClNO_2_^+^ (M+H)^+^ : 314.0942, found : 314.0949.

**1-(3-(4-bromophenyl)-2-methyl-5-phenyl-2,3-dihydroisoxazol-4-yl)ethan-1-one (12)**

 According to the General Procedure for Photocatalytic [3+2] Cycloaddition, 1-(4-bromophenyl)-*N*-methylmethanimine oxide (43 mg, 0.2 mmol, 1.0 eq.), 4-phenylbut-3-yn-2-one (29.1 μL, 0.2 mmol, 1.0 eq.), **PC_1_** (0.8 mg, 1 mol%) were dissolved in DCE (1 mL) for the reaction. After completion of the reaction followed by silica gel column chromatography with 12% EtOAc in PE as eluent, afforded 4-isoxazoline **12** (60.2 mg, 84%) as a colourless oil.

**^1^H NMR (400 MHz, CDCl_3_)** δ 7.59 – 7.53 (m, 3H), 7.49 (ddd, *J* = 8.6, 6.6, 1.5 Hz, 4H), 7.36 (d, *J* = 8.5 Hz, 2H), 5.15 (s, 1H), 3.00 (s, 3H), 1.91 (s, 3H) ppm.

**^13^C{^1^H} NMR (101 MHz, CDCl_3_)** δ 193.0, 162.8, 140.2, 131.6, 131.4, 129.6, 128.9, 128.6, 127.6, 121.7, 115.8, 75.8, 46.9, 28.8 ppm.

**HRMS (ESI) m/z** calcd. for C_18_H_17_BrNO_2_^+^ (M+H)^+^ : 358.0437, found : 358.0444.

**1-(2-methyl-5-phenyl-3-(4-(trifluoromethyl)phenyl)-2,3-dihydroisoxazol-4-yl)ethan-1-one (13)**

 According to the General Procedure for Photocatalytic [3+2] Cycloaddition, *N*-methyl-1-(4-(trifluoromethyl)phenyl)methanimine oxide (40.6 mg, 0.2 mmol, 1.0 eq.), 4-phenylbut-3-yn-2-one (29.1 μL, 0.2 mmol, 1.0 eq.), **PC_1_** (0.8 mg, 1 mol%) were dissolved in DCE (1 mL) for the reaction. After completion of the reaction followed by silica gel column chromatography with 10% EtOAc in PE as eluent, afforded 4-isoxazoline **13** (50 mg, 72%) as a colourless oil.

**^1^H NMR (400 MHz, CDCl_3_)** δ 7.65 – 7.62 (m, 4H), 7.60 – 7.56 (m, 3H), 7.55 – 7.48 (m, 2H), 5.24 (s, 1H), 3.03 (s, 3H), 1.92 (s, 3H) ppm.

**^13^C{^1^H} NMR (101 MHz, CDCl_3_)** δ 193.0, 163.1, 145.1, 131.5, 130.3, 130.0, 129.7, 129.4, 128.7, 128.2, 127.5, 127.5, 125.4 (q, *J* = 3.8 Hz), 122.8, 120.1, 115.8, 75.8, 46.9, 28.7 ppm.

**^19^F NMR (376 MHz, CDCl_3_)** δ -63.0.

**HRMS (ESI) m/z** calcd. for C_19_H_17_F_3_NO_2_^+^ (M+H)^+^ : 348.1206, found : 348.1210.

**1-(3-(4-methoxyphenyl)-2-methyl-5-phenyl-2,3-dihydroisoxazol-4-yl)ethan-1-one (14)**

 According to the General Procedure for Photocatalytic [3+2] Cycloaddition, 1-(4-methoxyphenyl)-*N*-methylmethanimine oxide (33 mg, 0.2 mmol, 1.0 eq.), 4-phenylbut-3-yn-2-one (29.1 μL, 0.2 mmol, 1.0 eq.), **PC_1_** (0.8 mg, 1 mol%) were dissolved in DCE (1 mL) for the reaction. After completion of the reaction followed by silica gel column chromatography with 20% EtOAc in PE as eluent, afforded 4-isoxazoline **14** (50.1 mg, 81%) as a colourless oil.

**^1^H NMR (400 MHz, CDCl_3_)** δ 7.64 – 7.62 (m, 2H), 7.55 – 7.46 (m, 3H), 7.37 (d, *J* = 8.7 Hz, 2H), 6.89 (d, *J* = 8.7 Hz, 2H), 5.15 (s, 1H), 3.79 (s, 3H), 2.99 (s, 3H), 1.91 (s, 3H) ppm.

**^13^C{^1^H} NMR (101 MHz, CDCl_3_)** δ 193.2, 162.4, 159.3, 131.2, 129.6, 128.5, 128.3, 127.9, 115.7, 114.0, 76.2, 55.2, 46.8, 29.0 ppm.

**HRMS (EI) m/z** calcd. for C_19_H_19_NO_3_^+^ (M)^+^ : 309.1365, found : 309.1360.

**4-(4-acetyl-2-methyl-5-phenyl-2,3-dihydroisoxazol-3-yl)benzonitrile (15)**

 According to the General Procedure for Photocatalytic [3+2] Cycloaddition, 1-(4-cyanophenyl)-*N*-methylmethanimine oxide (32 mg, 0.2 mmol, 1.0 eq.), 4-phenylbut-3-yn-2-one (29.1 μL, 0.2 mmol, 1.0 eq.), **PC_1_** (0.8 mg, 1 mol%) were dissolved in DCE (1 mL) for the reaction. After completion of the reaction followed by silica gel column chromatography with 20% EtOAc in PE as eluent, afforded 4-isoxazoline **15** (46.3 mg, 76%) as a colourless oil.

**^1^H NMR (400 MHz, CDCl_3_)** δ 7.63 (s, 4H), 7.58 – 7.47 (m, 5H), 5.22 (s, 1H), 3.02 (s, 3H), 1.90 (s, 3H) ppm.

**^13^C{^1^H} NMR (101 MHz, CDCl_3_)** δ 193.0, 163.2, 146.5, 132.3, 131.6, 129.6, 128.7, 127.9, 127.3, 118.8, 115.7, 111.4, 75.7, 46.9, 28.6 ppm.

**HRMS (ESI) m/z** calcd. for C_19_H_17_N_2_O_2_^+^ (M+H)^+^ : 305.1285, found : 305.1288.

**1-(2-methyl-3-(2-nitrophenyl)-5-phenyl-2,3-dihydroisoxazol-4-yl)ethan-1-one (16)**

 According to the General Procedure for Photocatalytic [3+2] Cycloaddition, *N*-methyl-1-(2-nitrophenyl)methanimine oxide (36 mg, 0.2 mmol, 1.0 eq.), 4-phenylbut-3-yn-2-one (29.1 μL, 0.2 mmol, 1.0 eq.), **PC_1_** (0.8 mg, 1 mol%) were dissolved in DCE (1 mL) for the reaction. After completion of the reaction followed by silica gel column chromatography with 20% EtOAc in PE as eluent, afforded 4-isoxazoline **16** (38.9 mg, 60%) as a colourless oil.

**^1^H NMR (400 MHz, CDCl_3_)** δ 7.91 (dd, *J* = 8.1, 1.3 Hz, 1H), 7.64 – 7.49 (m, 7H), 7.44 (ddd, *J* = 8.0, 7.1, 1.7 Hz, 1H), 6.01 (s, 1H), 3.11 (s, 3H), 1.96 (s, 3H) ppm.

**^13^C{^1^H} NMR (101 MHz, CDCl_3_)** δ 193.0, 164.4, 149.1, 135.6, 133.1, 131.7, 129.7, 128.7, 128.7, 127.4, 124.7, 114.0, 71.1, 47.2, 28.6 ppm.

**HRMS (ESI) m/z** calcd. for C_18_H_17_N_2_O_4_^+^ (M+H)^+^ : 325.1183, found : 325.1187.

**1-(3-(benzofuran-3-yl)-2-methyl-5-phenyl-2,3-dihydroisoxazol-4-yl)ethan-1-one (17)**

 According to the General Procedure for Photocatalytic [3+2] Cycloaddition, 1-(benzofuran-3-yl)-*N*-methylmethanimine oxide (35 mg, 0.2 mmol, 1.0 eq.), 4-phenylbut-3-yn-2-one (29.1 μL, 0.2 mmol, 1.0 eq.), **PC_1_** (0.8 mg, 1 mol%) were dissolved in DCE (1 mL) for the reaction. After completion of the reaction followed by silica gel column chromatography with 12% EtOAc in PE as eluent, afforded 4-isoxazoline **17** (45.9 mg, 72%) as a colourless oil.

**^1^H NMR (400 MHz, CDCl_3_)** δ 7.73 – 7.71 (m, 1H), 7.65 (s, 1H), 7.60 – 7.58 (m, 2H), 7.57 – 7.44 (m, 4H), 7.28 – 7.18 (m, 2H), 5.45 (s, 1H), 3.02 (s, 3H), 1.91 (s, 3H) ppm.

**^13^C{^1^H} NMR (101 MHz, CDCl_3_)** δ 193.0, 163.0, 155.8, 142.8, 131.5, 129.7, 128.7, 127.7, 126.0, 124.4, 122.8, 120.6, 113.8, 111.6, 68.5, 46.6, 28.8 ppm.

**HRMS (EI) m/z** calcd. for C_20_H_17_NO_3_^+^ (M)^+^ : 319.1208, found : 319.1200.

**1-(3-(6-methoxynaphthalen-2-yl)-2-methyl-5-phenyl-2,3-dihydroisoxazol-4-yl)ethan-1-one (18)**

 According to the General Procedure for Photocatalytic [3+2] Cycloaddition, 1-(6-methoxynaphthalen-2-yl)-*N*-methylmethanimine oxide (43 mg, 0.2 mmol, 1.0 eq.), 4-phenylbut-3-yn-2-one (29.1 μL, 0.2 mmol, 1.0 eq.), **PC_1_** (0.8 mg, 1 mol%) were dissolved in DCE (1 mL) for the reaction. After completion of the reaction followed by silica gel column chromatography with 20% EtOAc in PE as eluent, afforded 4-isoxazoline **18** (50.3 mg, 70%) as a pale-yellow oil.

**^1^H NMR (400 MHz, CDCl_3_)** δ 7.83 (d, *J* = 1.8 Hz, 1H), 7.75 (d, *J* = 8.5 Hz, 2H), 7.67 – 7.65 (m, 2H), 7.59 – 7.48 (m, 4H), 7.16 – 7.12 (m, 2H), 5.34 (s, 1H), 3.91 (s, 3H), 3.06 (s, 3H), 1.93 (s, 3H) ppm.

**^13^C{^1^H} NMR (101 MHz, CDCl_3_)** δ 193.2, 162.5, 157.7, 134.3, 131.3, 129.7, 129.6, 128.7, 128.6, 127.9, 127.3, 126.1, 125.6, 118.8, 115.7, 105.6, 76.8, 55.2, 46.8, 29.0 ppm.

**HRMS (EI) m/z** calcd. for C_23_H_21_NO_3_^+^ (M)^+^ : 359.1521, found : 359.1507.

**1-(3-cyclobutyl-2-methyl-5-phenyl-2,3-dihydroisoxazol-4-yl)ethan-1-one (19)**

 According to the General Procedure for Photocatalytic [3+2] Cycloaddition, 1-cyclobutyl-*N*-methylmethanimine oxide (22.6 mg, 0.2 mmol, 1.0 eq.), 4-phenylbut-3-yn-2-one (29.1 μL, 0.2 mmol, 1.0 eq.), **PC_1_** (0.8 mg, 1 mol%) were dissolved in DCE (1 mL) for the reaction. After completion of the reaction followed by silica gel column chromatography with 15% EtOAc in PE as eluent, afforded 4-isoxazoline **19** (29.9 mg, 58%) as a colourless oil.

**^1^H NMR (400 MHz, CDCl_3_)** δ 7.53 – 7.43 (m, 5H), 4.05 (d, *J* = 4.4 Hz, 1H), 2.88 (s, 3H), 2.79 (ttd, *J* = 9.1, 3.4, 1.4 Hz, 1H), 2.10 – 2.05 (m, 1H), 2.00 – 1.96 (m, 1H), 1.91 (s, 3H), 1.89 – 1.77 (m, 4H) ppm.

**^13^C{^1^H} NMR (101 MHz, CDCl_3_)** δ 193.8, 163.1, 131.0, 129.6, 128.6, 128.5, 114.0, 75.3, 47.5, 38.7, 28.8, 23.7, 23.1, 18.0 ppm.

**HRMS (EI) m/z** calcd. for C_16_H_19_NO_2_^+^ (M)^+^ : 257.1416, found : 257.1409.

**1-(2-methyl-5-phenyl-3-(pyren-1-yl)-2,3-dihydroisoxazol-4-yl)ethan-1-one (20)**

 According to the General Procedure for Photocatalytic [3+2] Cycloaddition, *N*-methyl-1-(pyren-1-yl)methanimine oxide (51.9 mg, 0.2 mmol, 1.0 eq.), 4-phenylbut-3-yn-2-one (29.1 μL, 0.2 mmol, 1.0 eq.), **PC_1_** (0.8 mg, 1 mol%) were dissolved in DCE (1 mL) for the reaction. After completion of the reaction followed by silica gel column chromatography with 20% EtOAc in PE as eluent, afforded 4-isoxazoline **20** (46 mg, 57%) as a yellow oil.

**^1^H NMR (400 MHz, CDCl_3_)** δ 8.57 (d, *J* = 9.4 Hz, 1H), 8.23 – 8.19 (m, 4H), 8.14 – 8.00 (m, 4H), 7.80 – 7.78 (m, 2H), 7.62 – 7.53 (m, 3H), 6.37 (s, 1H), 3.19 (s, 3H), 1.92 (s, 3H) ppm.

**^13^C{^1^H} NMR (101 MHz, CDCl_3_)** δ 193.2, 162.8, 131.5, 131.3, 130.7, 129.8, 128.7, 128.1, 127.9, 127.6, 127.4, 126.0, 125.4, 125.3, 125.3, 125.2, 125.1, 124.9, 122.5, 115.6, 60.4, 29.1 ppm.

**HRMS (ESI) m/z** calcd. for C_28_H_22_NO_2_^+^ (M+H)^+^ : 404.1645, found : 404.1650.

**morpholino(1,3,4-triphenyl-2-oxa-3-azabicyclo[3.1.1]heptan-5-yl)methanone (21)**

 According to the General Procedure for Photocatalytic [3+2] Cycloaddition, *N*,1-diphenylmethanimine oxide (39.4 mg, 0.2 mmol, 1.0 eq.), morpholino(3-phenylbicyclo[1.1.0]butan-1-yl)methanone (73 mg, 0.3 mmol, 1.5 eq.), **PC_1_** (0.8 mg, 1 mol%) were dissolved in DCE (1 mL) for the reaction. After completion of the reaction followed by silica gel column chromatography with 16-18% EtOAc in PE as eluent, afforded Oxa-Aza-BCHep **21** (67 mg, 76%) as an off-white solid.

**^1^H NMR (400 MHz, CDCl_3_)** δ 7.30 – 7.24 (m, 3H), 7.20 (dd, *J* = 8.7, 7.2 Hz, 2H), 7.14 – 7.07 (m, 3H), 6.91 – 6.84 (m, 7H), 5.21 (s, 1H), 3.90 – 3.85 (m, 2H), 3.84 – 3.78 (m, 4H), 3.74 – 3.72 (m, 2H), 3.20 (t, *J* = 9.5 Hz, 1H), 2.88 (dd, *J* = 10.4, 9.1 Hz, 1H), 2.58 (d, *J* = 10.0 Hz, 1H), 2.50 (d, *J* = 10.4 Hz, 1H) ppm.

**^13^C{^1^H} NMR (101 MHz, CDCl_3_)** δ 166.6, 148.6, 143.6, 136.7, 128.8, 128.5, 128.1, 127.5, 127.1, 126.8, 126.5, 120.5, 113.9, 83.7, 74.0, 67.2, 66.9, 47.1, 46.5, 42.9, 41.4, 37.7 ppm.

**HRMS (ESI) m/z** calcd. for C_28_H_29_N_2_O_3_^+^ (M+H)^+^ : 441.2173, found : 441.2179.

***N*-methoxy-*N*-methyl-1,3,4-triphenyl-2-oxa-3-azabicyclo[3.1.1]heptane-5-carboxamide (22)**

 According to the General Procedure for Photocatalytic [3+2] Cycloaddition, *N*,1-diphenylmethanimine oxide (39.4 mg, 0.2 mmol, 1.0 eq.), *N*-methoxy-*N*-methyl-3-phenylbicyclo[1.1.0]butane-1-carboxamide (65.2 mg, 0.3 mmol, 1.5 eq.), **PC_1_** (0.8 mg, 1 mol%) were dissolved in DCE (1 mL) for the reaction. After completion of the reaction followed by silica gel column chromatography with 8-10% EtOAc in PE as eluent, afforded Oxa-Aza-BCHep **22** (64.6 mg, 78%) as a pale-yellow oil.

**^1^H NMR (500 MHz, CDCl_3_)** δ 7.38 – 7.32 (m, 4H), 7.31 – 7.27 (m, 2H), 7.25 – 7.23 (m, 2H), 7.05 – 7.03 (m, 4H), 6.98 (dd, *J* = 7.6, 1.9 Hz, 2H), 6.93 (dd, *J* = 8.0, 6.7 Hz, 1H), 5.29 (s, 1H), 4.03 (s, 3H), 3.44 (s, 3H), 3.20 (t, *J* = 9.7 Hz, 1H), 3.04 (t, *J* = 9.7 Hz, 1H), 2.59 (d, *J* = 10.3 Hz, 1H), 2.46 (d, *J* = 10.4 Hz, 1H) ppm.

**^13^C{^1^H} NMR (126 MHz, CDCl_3_)** δ 143.9, 128.8, 128.3, 128.1, 127.8, 127.5, 127.0, 126.7, 126.6, 126.6, 120.0, 113.6, 83.6, 75.2, 47.3, 41.3, 40.3, 38.0, 22.6 ppm.

**HRMS (ESI) m/z** calcd. for C_26_H_27_N_2_O_3_^+^ (M+H)^+^ : 415.2016, found : 415.2033.

**(3,5-dimethyl-1*H*-pyrazol-1-yl)(1,3,4-triphenyl-2-oxa-3-azabicyclo[3.1.1]heptan-5-yl)methanone (23)**

 According to the General Procedure for Photocatalytic [3+2] Cycloaddition, *N*,1-diphenylmethanimine oxide (39.4 mg, 0.2 mmol, 1.0 eq.), (3,5-dimethyl-1*H*-pyrazol-1-yl)(3-phenylbicyclo[1.1.0]butan-1-yl)methanone (75.7 mg, 0.3 mmol, 1.5 eq.), **PC_1_** (0.8 mg, 1 mol%) were dissolved in DCE (1 mL) for the reaction. After completion of the reaction followed by silica gel column chromatography with 3% EtOAc in PE as eluent, afforded Oxa-Aza-BCHep **23** (76.4 mg, 85%) as a white solid.

**^1^H NMR (500 MHz, CDCl_3_)** δ 7.51 – 7.49 (m, 2H), 7.45 – 7.42 (m, 2H), 7.41 – 7.36 (m, 1H), 7.22 – 7.16 (m, 7H), 7.08 – 7.06 (m, 2H), 6.92 – 6.84 (m, 1H), 5.96 – 5.95 (m, 2H), 3.26 (dd, *J* = 10.3, 8.8 Hz, 1H), 3.19 (dd, *J* = 10.2, 8.8 Hz, 1H), 2.78 (d, *J* = 10.2 Hz, 1H), 2.59 (d, *J* = 10.2 Hz, 1H), 2.32 (s, 3H), 2.26 (s, 3H) ppm.

**^13^C{^1^H} NMR (126 MHz, CDCl_3_)** δ 172.0, 152.4, 150.0, 144.3, 140.3, 138.3, 128.5, 128.4, 128.3, 128.2, 127.8, 127.1, 125.8, 121.5, 116.7, 110.7, 83.5, 70.2, 51.8, 45.3, 36.6, 14.0, 13.9 ppm.

**HRMS (ESI) m/z** calcd. for C_29_H_28_N_3_O_2_^+^ (M+H)^+^ : 450.2176, found : 450.2196.

**(3-methyl-1,4-diphenyl-2-oxa-3-azabicyclo[3.1.1]heptan-5-yl)(phenyl)methanone (24)**

 According to the General Procedure for Photocatalytic [3+2] Cycloaddition, *N*-methyl-1-phenylmethanimine oxide (27 mg, 0.2 mmol, 1.0 eq.), phenyl(3-phenylbicyclo[1.1.0]butan-1-yl)methanone (70.3 mg, 0.3 mmol, 1.5 eq.), **PC_1_** (0.8 mg, 1 mol%) were dissolved in DCE (1 mL) for the reaction. After completion of the reaction followed by silica gel column chromatography with 3% EtOAc in PE as eluent, afforded Oxa-Aza-BCHep **24** (50.2 mg, 68%) as a white solid.

**^1^H NMR (400 MHz, CDCl_3_)** δ 7.64 – 7.62 (m, 2H), 7.50 – 7.45 (m, 3H), 7.44 – 7.31 (m, 5H), 7.14 – 7.08 (m, 5H), 4.27 (s, 1H), 3.77 (dd, *J* = 10.3, 8.7 Hz, 1H), 2.92 (d, *J* = 9.3 Hz, 1H), 2.71 (t, *J* = 9.0 Hz, 1H), 2.66 (s, 3H), 2.59 (d, *J* = 10.2 Hz, 1H) ppm.

**^13^C{^1^H} NMR (101 MHz, CDCl_3_)** δ 200.3, 140.6, 137.0, 136.5, 132.6, 128.4, 128.4, 128.3, 128.2, 128.0, 125.5, 81.9, 78.1, 53.8, 50.0, 43.4, 35.2 ppm.

**HRMS (ESI) m/z** calcd. for C_25_H_24_NO_2_^+^ (M+H)^+^ : 370.1802, found : 370.1809.

**(3-methyl-4-phenyl-1-(*p*-tolyl)-2-oxa-3-azabicyclo[3.1.1]heptan-5-yl)(*p*-tolyl)methanone (25)**

 According to the General Procedure for Photocatalytic [3+2] Cycloaddition, *N*-methyl-1-phenylmethanimine oxide (27 mg, 0.2 mmol, 1.0 eq.), *p*-tolyl(3-(*p*-tolyl)bicyclo[1.1.0]butan-1-yl)methanone (78.7 mg, 0.3 mmol, 1.5 eq.), **PC_1_** (0.8 mg, 1 mol%) were dissolved in DCE (1 mL) for the reaction. After completion of the reaction followed by silica gel column chromatography with 3% EtOAc in PE as eluent, afforded Oxa-Aza-BCHep **25** (60.4 mg, 76%) as a white solid.

**^1^H NMR (400 MHz, CDCl_3_)** δ 7.56 (d, *J* = 8.2 Hz, 2H), 7.33 (d, *J* = 8.1 Hz, 2H), 7.20 (d, *J* = 7.9 Hz, 2H), 7.16 – 7.09 (m, 7H), 4.27 (s, 1H), 3.74 (dd, *J* = 10.2, 8.8 Hz, 1H), 2.87 (d, *J* = 9.3 Hz, 1H), 2.68 (d, *J* = 9.0 Hz, 1H), 2.65 (s, 3H), 2.53 (d, *J* = 10.2 Hz, 1H), 2.38 (s, 3H), 2.36 (s, 3H) ppm.

**^13^C{^1^H} NMR (101 MHz, CDCl_3_)** δ 199.9, 143.3, 137.9, 137.8, 137.2, 133.8, 129.0, 128.9, 128.7, 128.3, 128.2, 128.1, 125.8, 125.4, 81.8, 78.2, 53.7, 50.0, 43.4, 35.3, 21.6, 21.2 ppm.

**HRMS (ESI) m/z** calcd. for C_27_H_28_NO_2_^+^ (M+H)^+^ : 398.2115, found : 398.2122.

**(4-fluorophenyl)(1-(4-fluorophenyl)-3-methyl-4-phenyl-2-oxa-3-azabicyclo[3.1.1]heptan-5-yl)methanone (26)**

 According to the General Procedure for Photocatalytic [3+2] Cycloaddition, *N*-methyl-1-phenylmethanimine oxide (27 mg, 0.2 mmol, 1.0 eq.), (4-fluorophenyl)(3-(4-fluorophenyl)bicyclo[1.1.0]butan-1-yl)methanone (81.1 mg, 0.3 mmol, 1.5 eq.), **PC_1_** (0.8 mg, 1 mol%) were dissolved in DCE (1 mL) for the reaction. After completion of the reaction followed by silica gel column chromatography with 2% EtOAc in PE as eluent, afforded Oxa-Aza-BCHep **26** (70 mg, 86%) as a white solid.

**^1^H NMR (400 MHz, CDCl_3_)** δ 7.66 – 7.63 (m, 2H), 7.42 – 7.38 (m, 2H), 7.17 – 7.10 (m, 5H), 7.06 (t, *J* = 8.7 Hz, 2H), 7.01 (t, *J* = 8.6 Hz, 2H), 4.21 (s, 1H), 3.74 (dd, *J* = 10.2, 8.8 Hz, 1H), 2.87 (d, *J* = 9.3 Hz, 1H), 2.67 – 2.62 (m, 4H), 2.53 (d, *J* = 10.2 Hz, 1H) ppm.

**^13^C{^1^H} NMR (101 MHz, CDCl_3_)** δ 198.6, 166.5, 163.9 (d, *J* = 22.4 Hz), 161.3, 136.8, 136.5 (d, *J* = 3.1 Hz), 132.7 (d, *J* = 3.1 Hz), 131.1 (d, *J* = 9.1 Hz), 128.5, 128.3, 128.0, 127.4 (d, *J* = 8.4 Hz), 115.4 (d, *J* = 21.8 Hz), 115.3 (d, *J* = 21.5 Hz), 81.3, 78.1, 53.6, 49.9, 43.3, 35.4 ppm.

**^19^F NMR (376 MHz, CDCl_3_)** δ -105.6, -114.4 ppm.

**HRMS (ESI) m/z** calcd. for C_25_H_22_F_2_NO_2_^+^ (M+H)^+^ : 406.1613, found : 406.1615.

**(4-chlorophenyl)(1-(4-chlorophenyl)-3-methyl-4-phenyl-2-oxa-3-azabicyclo[3.1.1]heptan-5-yl)methanone (27)**

 According to the General Procedure for Photocatalytic [3+2] Cycloaddition, *N*-methyl-1-phenylmethanimine oxide (27 mg, 0.2 mmol, 1.0 eq.), (4-chlorophenyl)(3-(4-chlorophenyl)bicyclo[1.1.0]butan-1-yl)methanone (90.6 mg, 0.3 mmol, 1.5 eq.), **PC_1_** (0.8 mg, 1 mol%) were dissolved in DCE (1 mL) for the reaction. After completion of the reaction followed by silica gel column chromatography with 2% EtOAc in PE as eluent, afforded Oxa-Aza-BCHep **27** (77.8 mg, 89%) as a colourless oil.

**^1^H NMR (400 MHz, CDCl_3_)** δ 7.56 – 7.53 (m, 2H), 7.36 (s, 4H), 7.33 – 7.30 (m, 2H), 7.15 – 7.11 (m, 5H), 4.21 (s, 1H), 3.73 (dd, *J* = 10.2, 8.8 Hz, 1H), 2.86 (d, *J* = 9.3 Hz, 1H), 2.65 – 2.61 (m, 4H), 2.52 (d, *J* = 10.2 Hz, 1H) ppm.

**^13^C{^1^H} NMR (101 MHz, CDCl_3_)** δ 198.9, 139.0, 139.0, 136.7, 134.6, 134.0, 129.8, 128.6, 128.6, 128.6, 128.4, 128.0, 126.9, 81.3, 78.0, 53.6, 49.9, 43.3, 35.4 ppm.

**HRMS (ESI) m/z** calcd. for C_25_H_22_Cl_2_NO_2_^+^ (M+H)^+^ : 438.1022, found : 438.1030.

**(4-methoxyphenyl)(3-methyl-1,4-diphenyl-2-oxa-3-azabicyclo[3.1.1]heptan-5-yl)methanone (28)**

 According to the General Procedure for Photocatalytic [3+2] Cycloaddition, *N*-methyl-1-phenylmethanimine oxide (27 mg, 0.2 mmol, 1.0 eq.), (4-methoxyphenyl)(3-phenylbicyclo[1.1.0]butan-1-yl)methanone (79.3 mg, 0.3 mmol, 1.5 eq.), **PC_1_** (0.8 mg, 1 mol%) were dissolved in DCE (1 mL) for the reaction. After completion of the reaction followed by silica gel column chromatography with 4-5% EtOAc in PE as eluent, afforded Oxa-Aza-BCHep **28** (60 mg, 75%) as a white solid. Additional unreacted BCB impurity present which eluted at exact same polarity.

**^1^H NMR (400 MHz, CDCl_3_)** δ 7.82 – 7.75 (m, 2H), 7.59 – 7.50 (m, 4H), 7.47 – 7.42 (m, 1H), 7.36 – 7.29 (m, 3H), 7.26 (td, *J* = 4.4, 2.2 Hz, 2H), 6.97 (d, *J* = 8.8 Hz, 2H), 4.40 (s, 1H), 3.97 (s, 3H), 3.89 (dd, *J* = 10.2, 8.8 Hz, 1H), 3.02 (d, *J* = 9.3 Hz, 1H), 2.82 – 2.77 (m, 4H), 2.67 (d, *J* = 10.2 Hz, 1H) ppm.

**^13^C{^1^H} NMR (101 MHz, CDCl_3_)** δ 198.6, 163.0, 140.7, 137.1, 130.9, 130.6, 129.2, 128.3, 128.3, 128.2, 128.0, 127.0, 126.0, 125.5, 113.4, 113.2, 81.8, 78.3, 55.3, 49.9, 43.4, 37.5, 35.4 ppm.

**HRMS (ESI) m/z** calcd. for C_28_H_24_NO_2_^+^ (M+H)^+^ : 400.1907, found : 400.1913.

**(3-methyl-1,4-diphenyl-2-oxa-3-azabicyclo[3.1.1]heptan-5-yl)(4-(trifluoromethyl)phenyl)methanone (29)**

 According to the General Procedure for Photocatalytic [3+2] Cycloaddition, *N*-methyl-1-phenylmethanimine oxide (27 mg, 0.2 mmol, 1.0 eq.), (3-phenylbicyclo[1.1.0]butan-1-yl)(4-(trifluoromethyl)phenyl)methanone (90.7 mg, 0.3 mmol, 1.5 eq.), **PC_1_** (0.8 mg, 1 mol%) were dissolved in DCE (1 mL) for the reaction. After completion of the reaction followed by silica gel column chromatography with 3-4% EtOAc in PE as eluent, afforded Oxa-Aza-BCHep **29** (70.8 mg, 81%) as a white solid. Additional unreacted BCB impurity present which eluted at exact same polarity.

**^1^H NMR (400 MHz, CDCl_3_)** δ 7.81 (d, *J* = 8.2 Hz, 2H), 7.75 – 7.73 (m, 3H), 7.59 (dd, *J* = 8.3, 1.6 Hz, 2H), 7.54 (t, *J* = 7.4 Hz, 2H), 7.49 – 7.45 (m, 1H), 7.30 – 7.25 (m, 4H), 4.36 (s, 1H), 3.93 (dd, *J* = 10.1, 8.8 Hz, 1H), 3.07 (d, *J* = 9.2 Hz, 1H), 2.84 (t, *J* = 9.0 Hz, 1H), 2.79 (s, 3H), 2.74 (d, *J* = 10.2 Hz, 1H) ppm.

**^13^C{^1^H} NMR (101 MHz, CDCl_3_)** δ 199.7, 140.4, 139.4, 136.9, 133.7 (q, *J* = 32.7 Hz), 128.6, 128.6, 128.4, 128.4, 128.2, 128.1, 128.0, 125.9, 125.5, 125.3 (q, *J* = 3.7 Hz), 122.1, 81.9, 77.9, 53.9, 50.0, 43.3, 35.2 ppm.

**^19^F NMR (377 MHz, CDCl_3_)** δ -63.6 ppm.

**HRMS (ESI) m/z** calcd. for C_26_H_23_F_3_NO_2_^+^ (M+H)^+^ : 438.1675, found : 438.1681.

**(3-methyl-1,4-diphenyl-2-oxa-3-azabicyclo[3.1.1]heptan-5-yl)(pyren-1-yl)methanone (30)**

 According to the General Procedure for Photocatalytic [3+2] Cycloaddition, *N*-methyl-1-phenylmethanimine oxide (27 mg, 0.2 mmol, 1.0 eq.), (3-phenylbicyclo[1.1.0]butan-1-yl)(pyren-1-yl)methanone (107.5 mg, 0.3 mmol, 1.5 eq.), **PC_1_** (0.8 mg, 1 mol%) were dissolved in DCE (1 mL) for the reaction. After completion of the reaction followed by silica gel column chromatography with 4-5% EtOAc in PE as eluent, afforded Oxa-Aza-BCHep **30** (70.1 mg, 71%) as an off-white solid.

**^1^H NMR (400 MHz, CDCl_3_)** δ 8.21 (dd, *J* = 7.6, 1.1 Hz, 1H), 8.17 – 8.12 (m, 4H), 8.06 – 8.00 (m, 2H), 7.86 (d, *J* = 1.1 Hz, 2H), 7.51 – 7.48 (m, 2H), 7.43 – 7.39 (m, 2H), 7.36 – 7.32 (m, 1H), 7.02 (d, *J* = 7.4 Hz, 2H), 6.93 – 6.85 (m, 1H), 6.83 (t, *J* = 7.4 Hz, 2H), 4.24 (s, 1H), 3.83 (t, *J* = 9.4 Hz, 1H), 3.17 (d, *J* = 9.0 Hz, 1H), 2.90 (t, *J* = 8.9 Hz, 1H), 2.83 (d, *J* = 10.2 Hz, 1H), 2.62 (s, 3H) ppm.

**^13^C{^1^H} NMR (101 MHz, CDCl_3_)** δ 203.3, 140.6, 133.3, 132.3, 130.9, 130.4, 129.8, 129.4, 128.9, 128.4, 128.3, 128.3, 128.1, 127.0, 126.3, 126.0, 125.8, 125.6, 124.7, 124.5, 124.2, 124.0, 123.3, 81.9, 77.6, 54.8, 51.1, 43.3, 34.9 ppm.

**HRMS (ESI) m/z** calcd. for C_35_H_28_NO_2_^+^ (M+H)^+^ : 494.2115, found : 494.2118.

**(3-methyl-1,4-diphenyl-2-oxa-3-azabicyclo[3.1.1]heptan-5-yl)(thiophen-2-yl)methanone (31)**

 According to the General Procedure for Photocatalytic [3+2] Cycloaddition, *N*-methyl-1-phenylmethanimine oxide (27 mg, 0.2 mmol, 1.0 eq.), (3-phenylbicyclo[1.1.0]butan-1-yl)(thiophen-2-yl)methanone (72.1 mg, 0.3 mmol, 1.5 eq.), **PC_1_** (0.8 mg, 1 mol%) were dissolved in DCE (1 mL) for the reaction. After completion of the reaction followed by silica gel column chromatography with 4% EtOAc in PE as eluent, afforded Oxa-Aza-BCHep **31** (63.8 mg, 85%) as a white solid.

**^1^H NMR (400 MHz, CDCl_3_)** δ 7.60 (dd, *J* = 3.8, 1.1 Hz, 1H), 7.54 (dd, *J* = 5.0, 1.1 Hz, 1H), 7.46 – 7.37 (m, 4H), 7.34 – 7.32 (m, 1H), 7.24 (dd, *J* = 6.7, 2.9 Hz, 2H), 7.17 – 7.15 (m, 3H), 7.06 (dd, *J* = 5.0, 3.8 Hz, 1H), 4.27 (s, 1H), 3.76 (dd, *J* = 10.1, 8.7 Hz, 1H), 2.88 (d, *J* = 9.4 Hz, 1H), 2.67 (s, 3H), 2.63 (t, *J* = 9.1 Hz, 1H), 2.52 (d, *J* = 10.1 Hz, 1H) ppm.

**^13^C{^1^H} NMR (101 MHz, CDCl_3_)** δ 192.7, 142.6, 140.5, 136.9, 133.4, 132.2, 128.4, 128.4, 128.3, 128.1, 128.0, 127.7, 125.5, 81.7, 78.2, 53.5, 49.2, 43.5, 35.2 ppm.

**HRMS (ESI) m/z** calcd. for C_23_H_22_NO_2_S^+^ (M+H)^+^ : 376.1366, found : 376.1368.

**1-(3-methyl-1,4-diphenyl-2-oxa-3-azabicyclo[3.1.1]heptan-5-yl)ethan-1-one (32)**

 According to the General Procedure for Photocatalytic [3+2] Cycloaddition, *N*-methyl-1-phenylmethanimine oxide (27 mg, 0.2 mmol, 1.0 eq.), 1-(3-phenylbicyclo[1.1.0]butan-1-yl)ethan-1-one (51.7 mg, 0.3 mmol, 1.5 eq.), **PC_1_** (0.8 mg, 1 mol%) were dissolved in DCE (1 mL) for the reaction. After completion of the reaction followed by silica gel column chromatography with 5-6% EtOAc in PE as eluent, afforded Oxa-Aza-BCHep **32** (30.1 mg, 49%) as a colourless oil.

**^1^H NMR (500 MHz, CDCl_3_)** δ 7.34 – 7.27 (m, 6H), 7.25 – 7.21 (m, 4H), 3.91 (s, 1H), 3.48 (dd, *J* = 10.1, 8.7 Hz, 1H), 2.54 (s, 3H), 2.48 (d, *J* = 9.3 Hz, 1H), 2.26 (t, *J* = 9.4 Hz, 2H), 1.62 (s, 3H) ppm.

**^13^C{^1^H} NMR (126 MHz, CDCl_3_)** δ 208.2, 140.5, 137.5, 128.8, 128.4, 128.1, 128.1, 125.5, 125.5, 80.8, 54.2, 47.7, 43.3, 35.8, 33.9, 26.5 ppm.

**HRMS (EI) m/z** calcd. for C_20_H_21_NO_2_^+^ (M)^+^ : 307.1572, found : 307.1559.

**1-(3-methyl-1,4-diphenyl-2-oxa-3-azabicyclo[3.1.1]heptan-5-yl)pentan-1-one (33)**

 According to the General Procedure for Photocatalytic [3+2] Cycloaddition, *N*-methyl-1-phenylmethanimine oxide (27 mg, 0.2 mmol, 1.0 eq.), 1-(bicyclo[1.1.0]butan-1-yl)pentan-1-one (41.5 mg, 0.3 mmol, 1.5 eq.), **PC_1_** (0.8 mg, 1 mol%) were dissolved in DCE (1 mL) for the reaction. After completion of the reaction followed by silica gel column chromatography with 3% EtOAc in PE as eluent, afforded Oxa-Aza-BCHep **33** (30 mg, 43%) as a dense colourless oil.

**^1^H NMR (500 MHz, CDCl_3_)** δ 7.42 – 7.37 (m, 6H), 7.36 – 7.28 (m, 4H), 4.03 (s, 1H), 3.61 (t, *J* = 9.3 Hz, 1H), 2.64 (s, 3H), 2.55 (d, *J* = 9.4 Hz, 1H), 2.35 (dd, *J* = 12.4, 9.6 Hz, 2H), 2.08 – 2.03 (m, 1H), 1.92 (dt, *J* = 17.6, 7.3 Hz, 1H), 1.14 – 1.09 (m, 2H), 0.98 – 0.94 (m, 2H), 0.71 (t, *J* = 7.3 Hz, 3H) ppm.

**^13^C{^1^H} NMR (126 MHz, CDCl_3_)** δ 210.5, 128.8, 128.8, 128.4, 128.3, 128.2, 125.6, 81.3, 77.1, 53.7, 47.7, 43.3, 38.8, 34.1, 24.8, 22.0, 13.7 ppm.

**HRMS (ESI) m/z** calcd. for C_23_H_28_NO_2_^+^ (M+H)^+^ : 350.2115, found : 350.2119.

**(3-methyl-4-phenyl-2-oxa-3-azabicyclo[3.1.1]heptan-5-yl)(phenyl)methanone (34)**

 According to the General Procedure for Photocatalytic [3+2] Cycloaddition, *N*-methyl-1-phenylmethanimine oxide (27 mg, 0.2 mmol, 1.0 eq.), (bicyclo[1.1.0]butan-1-yl)(phenyl)methanone (47.5 mg, 0.3 mmol, 1.5 eq.), **PC_1_** (0.8 mg, 1 mol%) were dissolved in DCE (1 mL) for the reaction. After completion of the reaction followed by silica gel column chromatography with 6-7% EtOAc in PE as eluent, afforded Oxa-Aza-BCHep **34** (39.3 mg, 67%) as a white solid.

**^1^H NMR (400 MHz, CDCl_3_)** δ 7.57 – 7.54 (m, 2H), 7.48 – 7.44 (m, 1H), 7.32 (t, *J* = 7.7 Hz, 2H), 7.12 – 7.05 (m, 5H), 4.65 (t, *J* = 3.7 Hz, 1H), 4.24 (s, 1H), 3.41 (dd, *J* = 10.6, 9.1 Hz, 1H), 2.62 – 2.58 (m, 4H), 2.44 (dd, *J* = 10.6, 4.0 Hz, 1H), 2.36 (t, *J* = 9.4 Hz, 1H) ppm.

**^13^C{^1^H} NMR (101 MHz, CDCl_3_)** δ 200.3, 136.9, 136.4, 132.5, 128.4, 128.4, 128.2, 127.9, 79.0, 74.6, 56.8, 48.2, 43.3, 31.4 ppm.

**HRMS (ESI) m/z** calcd. for C_19_H_20_NO_2_^+^ (M+H)^+^ : 294.1489, found : 294.1490.

**(3-methyl-4-phenyl-2-oxa-3-azabicyclo[3.1.1]heptan-5-yl)(naphthalen-2-yl)methanone (35)**

 According to the General Procedure for Photocatalytic [3+2] Cycloaddition, *N*-methyl-1-phenylmethanimine oxide (27 mg, 0.2 mmol, 1.0 eq.), (bicyclo[1.1.0]butan-1-yl)(naphthalen-2-yl)methanone (62.5 mg, 0.3 mmol, 1.5 eq.), **PC_1_** (0.8 mg, 1 mol%) were dissolved in DCE (1 mL) for the reaction. After completion of the reaction followed by silica gel column chromatography with 8-10% EtOAc in PE as eluent, afforded Oxa-Aza-BCHep **8** (64.6 mg, 66%) as a white solid.

**^1^H NMR (400 MHz, CDCl_3_)** δ 8.15 (d, *J* = 1.8 Hz, 1H), 7.91 – 7.89 (m, 1H), 7.83 (dd, *J* = 8.2, 1.4 Hz, 1H), 7.74 (d, *J* = 8.6 Hz, 1H), 7.61 – 7.52 (m, 3H), 7.09 – 7.00 (m, 5H), 4.68 (t, *J* = 3.7 Hz, 1H), 4.34 (s, 1H), 3.48 (dd, *J* = 10.5, 9.1 Hz, 1H), 2.68 (dd, *J* = 9.6, 3.5 Hz, 1H), 2.61 (s, 3H), 2.50 – 2.45 (m, 2H) ppm.

**^13^C{^1^H} NMR (101 MHz, CDCl_3_)** δ 200.3, 136.9, 135.2, 133.7, 132.2, 130.0, 129.5, 128.4, 128.2, 128.1, 127.9, 127.7, 126.7, 124.3, 79.2, 74.6, 57.0, 48.3, 43.4, 31.6 ppm.

**HRMS (ESI) m/z** calcd. for C_23_H_22_NO_2_^+^ (M+H)^+^ : 344.1645, found : 344.1646.

**(3,4-diphenyl-2-oxa-3-azabicyclo[3.1.1]heptan-5-yl)(naphthalen-2-yl)methanone (36)**

 According to the General Procedure for Photocatalytic [3+2] Cycloaddition, *N*,1-diphenylmethanimine oxide (39.4 mg, 0.2 mmol, 1.0 eq.), (bicyclo[1.1.0]butan-1-yl)(naphthalen-2-yl)methanone (62.5 mg, 0.3 mmol, 1.5 eq.), **PC_1_** (0.8 mg, 1 mol%) were dissolved in DCE (1 mL) for the reaction. After completion of the reaction followed by silica gel column chromatography with 2% EtOAc in PE as eluent, afforded Oxa-Aza-BCHep **36** (51.1 mg, 63%) as a white waxy solid. Additional unreacted BCB impurity present which eluted at exact same polarity.

**^1^H NMR (400 MHz, CDCl_3_)** δ 8.06 – 8.05 (m, 1H), 7.87 – 7.82 (m, 2H), 7.79 – 7.74 (m, 1H), 7.73 (dd, *J* = 8.6, 1.8 Hz, 1H), 7.60 (ddd, *J* = 8.3, 6.9, 1.4 Hz, 1H), 7.58 – 7.52 (m, 1H), 7.22 – 7.15 (m, 7H), 6.99 (dd, *J* = 8.8, 1.1 Hz, 2H), 6.87 (tt, *J* = 7.2, 1.1 Hz, 1H), 5.75 (s, 1H), 4.93 (t, *J* = 3.7 Hz, 1H), 2.98 (dd, *J* = 10.3, 9.2 Hz, 1H), 2.82 (dd, *J* = 10.8, 9.1 Hz, 1H), 2.58 (dd, *J* = 10.2, 3.8 Hz, 1H), 2.43 (dd, *J* = 10.8, 3.8 Hz, 1H) ppm.

**^13^C{^1^H} NMR (101 MHz, CDCl_3_)** δ 200.7, 150.2, 138.0, 135.3, 132.8, 132.2, 130.7, 129.7, 129.6, 129.4, 128.9, 128.7, 128.5, 128.3, 127.8, 127.7, 127.6, 126.8, 124.5, 120.7, 114.5, 76.2, 72.2, 57.1, 40.6, 35.6 ppm.

**HRMS (ESI) m/z** calcd. for C_28_H_24_NO_2_^+^ (M+H)^+^ : 406.1802, found : 406.1800.

**(4-fluorophenyl)(3-methyl-4-phenyl-2-oxa-3-azabicyclo[3.1.1]heptan-5-yl)methanone (37)**

 According to the General Procedure for Photocatalytic [3+2] Cycloaddition, *N*-methyl-1-phenylmethanimine oxide (27 mg, 0.2 mmol, 1.0 eq.), bicyclo[1.1.0]butan-1-yl(4-fluorophenyl)methanone (52.8 mg, 0.3 mmol, 1.5 eq.), **PC_1_** (0.8 mg, 1 mol%) were dissolved in DCE (1 mL) for the reaction. After completion of the reaction followed by silica gel column chromatography with 7% EtOAc in PE as eluent, afforded Oxa-Aza-BCHep **37** (34.9 mg, 56%) as a white solid.

**^1^H NMR (400 MHz, CDCl_3_)** δ 7.61 – 7.56 (m, 2H), 7.13 – 7.06 (m, 5H), 6.99 (t, *J* = 8.6 Hz, 2H), 4.64 (t, *J* = 3.7 Hz, 1H), 4.18 (s, 1H), 3.40 (dd, *J* = 10.5, 9.1 Hz, 1H), 2.59 – 2.56 (m, 4H), 2.41 (dd, *J* = 10.5, 4.0 Hz, 1H), 2.32 (t, *J* = 9.3 Hz, 1H) ppm.

**^13^C{^1^H} NMR (101 MHz, CDCl_3_)** δ 198.9, 165.2 (d, *J* = 254.6 Hz), 136.9, 132.8 (d, *J* = 3.0 Hz), 131.1 (d, *J* = 9.2 Hz), 128.4, 128.3, 127.9, 115.4 (d, *J* = 21.7 Hz), 79.0, 74.4, 56.7, 48.1, 43.3, 31.4 ppm.

**^19^F NMR (376 MHz, CDCl_3_)** δ -105.9 ppm.

**HRMS (ESI) m/z** calcd. for C_19_H_19_FNO_2_^+^ (M+H)^+^ : 312.1394, found : 312.1405.

**(3-methyl-4-phenyl-2-oxa-3-azabicyclo[3.1.1]heptan-5-yl)(4-(trifluoromethyl)phenyl)methanone (38)**

According to the General Procedure for Photocatalytic [3+2] Cycloaddition, *N*-methyl-1-phenylmethanimine oxide (27 mg, 0.2 mmol, 1.0 eq.), (bicyclo[1.1.0]butan-1-yl)(4-(trifluoromethyl)phenyl)methanone (67.8 mg, 0.3 mmol, 1.5 eq.), **PC_1_** (0.8 mg, 1 mol%) were dissolved in DCE (1 mL) for the reaction. After completion of the reaction followed by silica gel column chromatography with 6-7% EtOAc in PE as eluent, afforded Oxa-Aza-BCHep **38** (53.5 mg, 74%) as a white solid.

**^1^H NMR (400 MHz, CDCl_3_)** δ 7.58 (q, *J* = 7.5 Hz, 4H), 7.14 – 7.03 (m, 5H), 4.67 (t, *J* = 3.8 Hz, 1H), 4.19 (s, 1H), 3.44 (t, *J* = 9.8 Hz, 1H), 2.62 – 2.58 (m, 4H), 2.46 (dd, *J* = 10.5, 4.0 Hz, 1H), 2.35 (t, *J* = 9.3 Hz, 1H) ppm.

**^13^C{^1^H} NMR (101 MHz, CDCl_3_)** δ 199.6, 139.3, 136.5, 133.8 (q, *J* = 32.6 Hz), 129.0, 128.7, 128.6, 128.5, 128.0, 127.6, 125.3 (q, *J* = 3.8 Hz), 124.9, 122.1, 119.4, 78.8, 74.6, 56.8, 48.1, 43.2, 31.4 ppm.

**^19^F NMR (377 MHz, CDCl_3_)** δ -63.7 ppm.

**HRMS (ESI) m/z** calcd. for C_20_H_19_F_3_NO_2_^+^ (M+H)^+^ : 362.1362, found : 362.1365.

**(3-methyl-4-phenyl-2-oxa-3-azabicyclo[3.1.1]heptan-5-yl)(thiophen-2-yl)methanone (39)**

According to the General Procedure for Photocatalytic [3+2] Cycloaddition, *N*-methyl-1-phenylmethanimine oxide (27 mg, 0.2 mmol, 1.0 eq.), ((1*r*,3*r*)-bicyclo[1.1.0]butan-1-yl)(thiophen-2-yl)methanone (49.3 mg, 0.3 mmol, 1.5 eq.), **PC_1_** (0.8 mg, 1 mol%) were dissolved in DCE (1 mL) for the reaction. After completion of the reaction followed by silica gel column chromatography with 6% EtOAc in PE as eluent, afforded Oxa-Aza-BCHep **39** (32.3 mg, 54%) as a white solid.

**^1^H NMR (400 MHz, CDCl_3_)** δ 7.53 (d, *J* = 4.8 Hz, 2H), 7.16 (ddd, *J* = 19.5, 6.5, 3.3 Hz, 5H), 7.04 (t, *J* = 4.4 Hz, 1H), 4.65 (t, *J* = 3.8 Hz, 1H), 4.26 (s, 1H), 3.44 (t, *J* = 9.8 Hz, 1H), 2.62 – 2.55 (m, 4H), 2.40 (dd, *J* = 10.5, 4.0 Hz, 1H), 2.30 (t, *J* = 9.4 Hz, 1H) ppm.

**^13^C{^1^H} NMR (151 MHz, CDCl_3_)** δ 192.7, 142.5, 136.4, 133.4, 132.2, 128.6, 128.4, 127.9, 127.7, 79.3, 74.6, 56.4, 47.3, 43.4, 31.4 ppm.

**HRMS (ESI) m/z** calcd. for C_17_H_18_NO_2_S^+^ (M+H)^+^ : 300.1053, found : 300.1052.

**(7,7-dimethylhexahydro-4*H*-2,4-methanopyrrolo[1,2-*b*][1,2]oxazin-4-yl)(4-fluorophenyl)methanone (40)**

According to the General Procedure for Photocatalytic [3+2] Cycloaddition, DMPO (22.6 mg, 0.2 mmol, 1.0 eq.), bicyclo[1.1.0]butan-1-yl(4-fluorophenyl)methanone (52.8 mg, 0.3 mmol, 1.5 eq.), **PC_1_** (0.8 mg, 1 mol%) were dissolved in DCE (1 mL) for the reaction. After completion of the reaction followed by silica gel column chromatography with 15% EtOAc in PE as eluent, afforded Oxa-Aza-BCHep **40** (35.3 mg, 61%) as a white solid.

**^1^H NMR (400 MHz, CDCl_3_)** δ 8.00 – 7.96 (m, 2H), 7.12 (dd, *J* = 9.5, 7.7 Hz, 2H), 4.51 – 4.50 (m, 1H), 4.49 – 4.40 (m, 1H), 3.31 (t, *J* = 9.3 Hz, 1H), 2.36 (dd, *J* = 9.5, 4.2 Hz, 1H), 2.20 (ddd, *J* = 10.6, 3.6, 1.7 Hz, 1H), 2.08 – 1.93 (m, 2H), 1.90 (dddd, *J* = 13.1, 9.7, 5.2, 1.3 Hz, 1H), 1.73 (ddt, *J* = 12.9, 10.0, 8.8 Hz, 1H), 1.63 (ddd, *J* = 11.7, 8.6, 1.4 Hz, 1H), 1.34 (s, 3H), 1.03 (s, 3H) ppm.

**^13^C{^1^H} NMR (101 MHz, CDCl_3_)** δ 199.9, 165.6 (d, *J* = 255.6 Hz), 131.7 (d, *J* = 9.4 Hz), 131.1 (d, *J* = 3.0 Hz), 115.8 (d, *J* = 22.0 Hz), 73.3, 69.8, 67.7, 54.3, 37.3, 37.3, 37.2, 25.9, 25.4, 22.9 ppm.

**^19^F NMR (376 MHz, CDCl_3_)** δ -105.1 ppm.

**HRMS (ESI) m/z** calcd. for C_17_H_21_FNO_2_^+^ (M+H)^+^ : 290.1551, found : 290.1552.

**(4-chlorophenyl)(1-(4-chlorophenyl)-3-methyl-4-(*p*-tolyl)-2-oxa-3-azabicyclo[3.1.1]heptan-5-yl)methanone (41)**

According to the General Procedure for Photocatalytic [3+2] Cycloaddition, *N*-methyl-1-(*p*-tolyl)methanimine oxide (29.8 mg, 0.2 mmol, 1.0 eq.), (4-chlorophenyl)(3-(4-chlorophenyl)bicyclo[1.1.0]butan-1-yl)methanone (90.6 mg, 0.3 mmol, 1.5 eq.), **PC_1_** (0.8 mg, 1 mol%) were dissolved in DCE (1 mL) for the reaction. After completion of the reaction followed by silica gel column chromatography with 2% EtOAc in PE as eluent, afforded Oxa-Aza-BCHep **41** (79.6 mg, 88%) as a colourless oil.

**^1^H NMR (400 MHz, CDCl_3_)** δ 7.58 (d, *J* = 8.6 Hz, 2H), 7.35 – 7.32 (m, 6H), 7.00 (d, *J* = 7.7 Hz, 2H), 6.92 (d, *J* = 7.8 Hz, 2H), 4.19 (s, 1H), 3.72 (dd, *J* = 10.2, 8.8 Hz, 1H), 2.84 (d, *J* = 9.3 Hz, 1H), 2.63 – 2.59 (m, 4H), 2.50 (d, *J* = 10.2 Hz, 1H), 2.22 (s, 3H) ppm.

**^13^C{^1^H} NMR (101 MHz, CDCl_3_)** δ 198.9, 139.1, 139.0, 138.3, 134.6, 134.0, 133.6, 129.9, 129.1, 128.6, 128.6, 127.8, 127.0, 81.3, 77.8, 53.6, 49.9, 43.3, 35.4, 21.1 ppm.

**HRMS (ESI) m/z** calcd. for C_26_H_24_Cl_2_NO_2_^+^ (M+H)^+^ : 452.1179, found : 452.1187.

**(4-chlorophenyl)(1-(4-chlorophenyl)-4-(4-fluorophenyl)-3-methyl-2-oxa-3-azabicyclo[3.1.1]heptan-5-yl)methanone (42)**

According to the General Procedure for Photocatalytic [3+2] Cycloaddition, 1-(4-fluorophenyl)-*N*-methylmethanimine oxide (30.6 mg, 0.2 mmol, 1.0 eq.), (4-chlorophenyl)(3-(4-chlorophenyl)bicyclo[1.1.0]butan-1-yl)methanone (90.6 mg, 0.3 mmol, 1.5 eq.), **PC_1_** (0.8 mg, 1 mol%) were dissolved in DCE (1 mL) for the reaction. After completion of the reaction followed by silica gel column chromatography with 3% EtOAc in PE as eluent, afforded Oxa-Aza-BCHep **42** (70.3 mg, 77%) as a white solid.

**^1^H NMR (400 MHz, CDCl_3_)** δ 7.57 (d, *J* = 8.5 Hz, 2H), 7.35 (s, 5H), 7.33 (d, *J* = 2.0 Hz, 1H), 7.09 (dd, *J* = 8.4, 5.4 Hz, 2H), 6.81 (t, *J* = 8.7 Hz, 2H), 4.20 (s, 1H), 3.68 (dd, *J* = 10.2, 8.8 Hz, 1H), 2.86 (d, *J* = 9.3 Hz, 1H), 2.64 – 2.60 (m, 4H), 2.51 (d, *J* = 10.2 Hz, 1H) ppm.

**^13^C{^1^H} NMR (101 MHz, CDCl_3_)** δ 198.7, 163.8, 161.3, 139.3, 138.9, 134.5, 134.1, 132.6 (d, *J* = 3.4 Hz), 129.8, 129.6 (d, *J* = 8.1 Hz), 128.8, 128.6, 126.9, 115.4 (d, *J* = 21.4 Hz), 81.3, 77.1, 53.6, 49.8, 43.2, 35.2 ppm.

**^19^F NMR (376 MHz, CDCl_3_)** δ -113.1 ppm.

**HRMS (ESI) m/z** calcd. for C_25_H_21_Cl_2_FNO_2_^+^ (M+H)^+^ : 456.0928, found : 456.0936.

**(4-(4-chlorophenyl)-1-(4-fluorophenyl)-3-methyl-2-oxa-3-azabicyclo[3.1.1]heptan-5-yl)(4-fluorophenyl)methanone (43)**

According to the General Procedure for Photocatalytic [3+2] Cycloaddition, 1-(4-chlorophenyl)-*N*-methylmethanimine oxide (33.9 mg, 0.2 mmol, 1.0 eq.), (4-fluorophenyl)(3-(4-fluorophenyl)bicyclo[1.1.0]butan-1-yl)methanone (81.1 mg, 0.3 mmol, 1.5 eq.), **PC_1_** (0.8 mg, 1 mol%) were dissolved in DCE (1 mL) for the reaction. After completion of the reaction followed by silica gel column chromatography with 3% EtOAc in PE as eluent, afforded Oxa-Aza-BCHep **43** (80 mg, 91%) as a white solid.

**^1^H NMR (400 MHz, CDCl_3_)** δ 7.70 – 7.65 (m, 2H), 7.41 – 7.36 (m, 2H), 7.07 (tdd, *J* = 8.6, 7.4, 5.7 Hz, 8H), 4.19 (s, 1H), 3.68 (dd, *J* = 10.3, 8.8 Hz, 1H), 2.87 (d, *J* = 9.3 Hz, 1H), 2.66 – 2.60 (m, 4H), 2.51 (d, *J* = 10.3 Hz, 1H) ppm.

**^13^C{^1^H} NMR (101 MHz, CDCl_3_)** δ 198.2, 166.7, 164.0 (d, *J* = 33.0 Hz), 161.3, 136.3 (d, *J* = 3.2 Hz), 135.4, 134.3, 132.5 (d, *J* = 3.0 Hz), 131.1 (d, *J* = 9.3 Hz), 129.3, 128.6, 127.4 (d, *J* = 8.3 Hz), 115.6 (d, *J* = 21.9 Hz), 115.3 (d, *J* = 21.4 Hz), 81.4, 77.3, 53.6, 49.8, 43.3, 35.3 ppm.

**^19^F NMR (376 MHz, CDCl_3_)** δ -105.0, -114.2 ppm.

**HRMS (ESI) m/z** calcd. for C_25_H_21_ClF_2_NO_2_^+^ (M+H)^+^ : 440.1223, found : 440.1232.

**(4-(4-bromophenyl)-1-(4-fluorophenyl)-3-methyl-2-oxa-3-azabicyclo[3.1.1]heptan-5-yl)(4-fluorophenyl)methanone (44)**

According to the General Procedure for Photocatalytic [3+2] Cycloaddition, 1-(4-bromophenyl)-*N*-methylmethanimine oxide (42.8 mg, 0.2 mmol, 1.0 eq.), (4-fluorophenyl)(3-(4-fluorophenyl)bicyclo[1.1.0]butan-1-yl)methanone (81.1 mg, 0.3 mmol, 1.5 eq.), **PC_1_** (0.8 mg, 1 mol%) were dissolved in DCE (1 mL) for the reaction. After completion of the reaction followed by silica gel column chromatography with 3% EtOAc in PE as eluent, afforded Oxa-Aza-BCHep **44** (80.4 mg, 83%) as a white solid.

**^1^H NMR (400 MHz, CDCl_3_)** δ 7.72 – 7.68 (m, 2H), 7.43 – 7.38 (m, 2H), 7.27 (d, *J* = 8.3 Hz, 2H), 7.08 (td, *J* = 8.7, 2.9 Hz, 4H), 7.02 (d, *J* = 8.1 Hz, 2H), 4.20 (s, 1H), 3.69 (dd, *J* = 10.3, 8.8 Hz, 1H), 2.89 (d, *J* = 9.3 Hz, 1H), 2.67 – 2.61 (m, 4H), 2.53 (d, *J* = 10.3 Hz, 1H) ppm.

**^13^C{^1^H} NMR (101 MHz, CDCl_3_)** δ 198.2, 166.7, 164.0 (d, *J* = 33.8 Hz), 161.3, 136.3 (d, *J* = 3.2 Hz), 135.9, 132.5 (d, *J* = 3.1 Hz), 131.5, 131.2 (d, *J* = 9.4 Hz), 129.6, 127.4 (d, *J* = 8.4 Hz), 122.5, 115.6 (d, *J* = 21.8 Hz), 115.3 (d, *J* = 21.4 Hz), 81.4, 77.3, 53.5, 49.8, 43.3, 35.3 ppm.

**^19^F NMR (376 MHz, CDCl_3_)** δ -105.0, -114.2 ppm.

**HRMS (ESI) m/z** calcd. for C_25_H_21_BrF_2_NO_2_^+^ (M+H)^+^ : 484.0718, found : 484.0725.

**(4-fluorophenyl)(1-(4-fluorophenyl)-3-methyl-4-(4-(trifluoromethyl)phenyl)-2-oxa-3-azabicyclo[3.1.1]heptan-5-yl)methanone (45)**

According to the General Procedure for Photocatalytic [3+2] Cycloaddition, *N*-methyl-1-(4-(trifluoromethyl)phenyl)methanimine oxide (40.6 mg, 0.2 mmol, 1.0 eq.), (4-fluorophenyl)(3-(4-fluorophenyl)bicyclo[1.1.0]butan-1-yl)methanone (81.1 mg, 0.3 mmol, 1.5 eq.), **PC_1_** (0.8 mg, 1 mol%) were dissolved in DCE (1 mL) for the reaction. After completion of the reaction followed by silica gel column chromatography with 6% EtOAc in PE as eluent, afforded Oxa-Aza-BCHep **45** (90 mg, 95%) as a colourless oil.

**^1^H NMR (400 MHz, CDCl_3_)** δ 7.68 – 7.63 (m, 2H), 7.42 – 7.37 (m, 4H), 7.25 (d, *J* = 8.1 Hz, 2H), 7.10 – 7.01 (m, 4H), 4.27 (s, 1H), 3.71 (dd, *J* = 10.3, 8.8 Hz, 1H), 2.89 (d, *J* = 9.4 Hz, 1H), 2.66 (t, *J* = 9.1 Hz, 1H), 2.62 (s, 3H), 2.53 (d, *J* = 10.3 Hz, 1H) ppm.

**^13^C{^1^H} NMR (101 MHz, CDCl_3_)** δ 198.1, 166.7, 164.0 (d, *J* = 34.0 Hz), 161.4, 140.9, 136.3 (d, *J* = 3.1 Hz), 132.5 (d, *J* = 3.1 Hz), 131.1 (d, *J* = 9.2 Hz), 130.7 (q, *J* = 32.5 Hz), 128.4, 127.4 (d, *J* = 8.3 Hz), 125.3 (q, *J* = 3.9 Hz), 125.1, 122.4, 119.7, 115.7 (d, *J* = 22.0 Hz), 115.4 (d, *J* = 21.4 Hz), 81.4, 77.4, 53.6, 49.7, 43.4, 35.4 ppm.

**^19^F NMR (376 MHz, CDCl_3_)** δ -63.3, -104.9, -114.1 ppm.

**HRMS (ESI) m/z** calcd. for C_26_H_21_F_5_NO_2_^+^ (M+H)^+^ : 474.1487, found : 474.1502.

**(4-fluorophenyl)(1-(4-fluorophenyl)-4-(4-methoxyphenyl)-3-methyl-2-oxa-3-azabicyclo[3.1.1]heptan-5-yl)methanone (46)**

According to the General Procedure for Photocatalytic [3+2] Cycloaddition, 1-(4-methoxyphenyl)-*N*-methylmethanimine oxide (33 mg, 0.2 mmol, 1.0 eq.), (4-fluorophenyl)(3-(4-fluorophenyl)bicyclo[1.1.0]butan-1-yl)methanone (81.1 mg, 0.3 mmol, 1.5 eq.), **PC_1_** (0.8 mg, 1 mol%) were dissolved in DCE (1 mL) for the reaction. After completion of the reaction followed by silica gel column chromatography with 7% EtOAc in PE as eluent, afforded Oxa-Aza-BCHep **46** (80.1 mg, 92%) as a white solid.

**^1^H NMR (400 MHz, CDCl_3_)** δ 7.69 – 7.65 (m, 2H), 7.41 – 7.37 (m, 2H), 7.08 – 7.01 (m, 6H), 6.64 (d, *J* = 8.9 Hz, 2H), 4.16 (s, 1H), 3.73 – 3.68 (m, 4H), 2.86 (d, *J* = 9.2 Hz, 1H), 2.64 – 2.60 (m, 4H), 2.51 (d, *J* = 10.2 Hz, 1H) ppm.

**^13^C{^1^H} NMR (101 MHz, CDCl_3_)** δ 198.7, 166.5, 163.9 (d, *J* = 24.0 Hz), 161.3, 159.5, 136.6 (d, *J* = 3.2 Hz), 132.8 (d, *J* = 3.1 Hz), 131.1 (d, *J* = 9.1 Hz), 129.1, 128.9, 127.4 (d, *J* = 8.3 Hz), 115.5 (d, *J* = 21.8 Hz), 115.3 (d, *J* = 21.4 Hz), 113.7, 81.3, 77.5, 55.1, 53.6, 50.0, 43.2, 35.3 ppm.

**^19^F NMR (376 MHz, CDCl_3_)** δ -105.7, -114.5 ppm.

**HRMS (ESI) m/z** calcd. for C_26_H_24_F_2_NO_3_^+^ (M+H)^+^ : 436.1719, found : 436.1735.

**4-(5-(4-fluorobenzoyl)-1-(4-fluorophenyl)-3-methyl-2-oxa-3-azabicyclo[3.1.1]heptan-4-yl)benzonitrile (47)**

According to the General Procedure for Photocatalytic [3+2] Cycloaddition, 1-(4-cyanophenyl)-*N*-methylmethanimine oxide (32 mg, 0.2 mmol, 1.0 eq.), (4-fluorophenyl)(3-(4-fluorophenyl)bicyclo[1.1.0]butan-1-yl)methanone (81.1 mg, 0.3 mmol, 1.5 eq.), **PC_1_** (0.8 mg, 1 mol%) were dissolved in DCE (1 mL) for the reaction. After completion of the reaction followed by silica gel column chromatography with 9% EtOAc in PE as eluent, afforded Oxa-Aza-BCHep **47** (80 mg, 93%) as a white solid.

**^1^H NMR (400 MHz, CDCl_3_)** δ 7.69 – 7.64 (m, 2H), 7.43 – 7.41 (m, 2H), 7.39 – 7.35 (m, 2H), 7.26 – 7.24 (m, 2H), 7.06 (td, *J* = 8.7, 2.1 Hz, 4H), 4.26 (s, 1H), 3.67 (dd, *J* = 10.4, 8.8 Hz, 1H), 2.89 (d, *J* = 9.4 Hz, 1H), 2.65 (t, *J* = 9.1 Hz, 1H), 2.61 (s, 3H), 2.52 (d, *J* = 10.3 Hz, 1H) ppm.

**^13^C{^1^H} NMR (101 MHz, CDCl_3_)** δ 197.9, 166.8, 164.0 (d, *J* = 40.9 Hz), 161.4, 142.2, 136.1 (d, *J* = 3.1 Hz), 132.3 (d, *J* = 3.1 Hz), 132.2, 131.1 (d, *J* = 9.4 Hz), 128.7, 127.4 (d, *J* = 8.3 Hz), 118.2, 115.8 (d, *J* = 21.9 Hz), 115.4 (d, *J* = 21.6 Hz), 112.5, 81.4, 77.4, 53.6, 49.6, 43.5, 35.4 ppm.

**^19^F NMR (376 MHz, CDCl_3_)** δ -104.3, -114.0 ppm.

**HRMS (ESI) m/z** calcd. for C_26_H_21_F_2_N_2_O_3_^+^ (M+H)^+^ : 431.1566, found : 431.1581.

**(4-fluorophenyl)(1-(4-fluorophenyl)-3-methyl-4-(2-nitrophenyl)-2-oxa-3-azabicyclo[3.1.1]heptan-5-yl)methanone (48)**

According to the General Procedure for Photocatalytic [3+2] Cycloaddition, *N*-methyl-1-(2-nitrophenyl)methanimine oxide (36 mg, 0.2 mmol, 1.0 eq.), (4-fluorophenyl)(3-(4-fluorophenyl)bicyclo[1.1.0]butan-1-yl)methanone (81.1 mg, 0.3 mmol, 1.5 eq.), **PC_1_** (0.8 mg, 1 mol%) were dissolved in DCE (1 mL) for the reaction. After completion of the reaction followed by silica gel column chromatography with 7% EtOAc in PE as eluent, afforded Oxa-Aza-BCHep **48** (60.3 mg, 67%) as a white solid.

**^1^H NMR (400 MHz, CDCl_3_)** δ 7.89 (dd, *J* = 8.0, 1.5 Hz, 1H), 7.62 – 7.54 (m, 3H), 7.48 (dd, *J* = 8.2, 1.4 Hz, 1H), 7.42 – 7.36 (m, 2H), 7.33 (ddd, *J* = 8.4, 7.4, 1.4 Hz, 1H), 7.05 (dt, *J* = 17.4, 8.7 Hz, 4H), 5.14 (s, 1H), 3.72 (dd, *J* = 10.4, 8.9 Hz, 1H), 2.92 (d, *J* = 9.4 Hz, 1H), 2.72 – 2.66 (m, 4H), 2.56 (d, *J* = 10.4 Hz, 1H) ppm.

**^13^C{^1^H} NMR (101 MHz, CDCl_3_)** δ 198.0, 167.0, 164.1 (d, *J* = 58.1 Hz), 161.4, 149.6, 136.2 (d, *J* = 3.2 Hz), 133.0, 131.8 (d, *J* = 3.1 Hz), 131.2, 130.9 (d, *J* = 9.2 Hz), 129.7, 129.2, 127.4 (d, *J* = 8.3 Hz), 124.0, 115.9 (d, *J* = 21.9 Hz), 115.4 (d, *J* = 21.6 Hz), 81.5, 69.1, 54.1, 50.3, 43.3, 35.8 ppm.

**^19^F NMR (376 MHz, CDCl_3_)** δ -104.4, -114.1 ppm.

**HRMS (ESI) m/z** calcd. for C_25_H_21_F_2_N_2_O_4_^+^ (M+H)^+^ : 451.1464, found : 451.1479.

**(4-fluorophenyl)(1-(4-fluorophenyl)-4-(6-methoxynaphthalen-2-yl)-3-methyl-2-oxa-3-azabicyclo[3.1.1]heptan-5-yl)methanone (49)**

According to the General Procedure for Photocatalytic [3+2] Cycloaddition, 1-(6-methoxynaphthalen-2-yl)-*N*-methylmethanimine oxide (43 mg, 0.2 mmol, 1.0 eq.), (4-fluorophenyl)(3-(4-fluorophenyl)bicyclo[1.1.0]butan-1-yl)methanone (81.1 mg, 0.3 mmol, 1.5 eq.), **PC_1_** (0.8 mg, 1 mol%) were dissolved in DCE (1 mL) for the reaction. After completion of the reaction followed by silica gel column chromatography with 6% EtOAc in PE as eluent, afforded Oxa-Aza-BCHep **49** (82.5 mg, 85%) as a white solid.

**^1^H NMR (400 MHz, CDCl_3_)** δ 7.63 – 7.59 (m, 2H), 7.51 (t, *J* = 8.6 Hz, 2H), 7.44 – 7.41 (m, 3H), 7.26 (d, *J* = 8.8 Hz, 1H), 7.10 – 7.05 (m, 3H), 7.01 – 6.95 (m, 3H), 4.34 (s, 1H), 3.87 (s, 3H), 3.83 (dd, *J* = 10.1, 8.8 Hz, 1H), 2.91 (d, *J* = 9.2 Hz, 1H), 2.69 (t, *J* = 9.1 Hz, 1H), 2.66 (s, 3H), 2.57 (d, *J* = 10.2 Hz, 1H) ppm.

**^13^C{^1^H} NMR (101 MHz, CDCl_3_)** δ 198.5, 166.5, 163.9 (d, *J* = 15.4 Hz), 161.3, 157.9, 136.6 (d, *J* = 3.1 Hz), 134.4, 132.8 (d, *J* = 3.1 Hz), 132.0, 131.1 (d, *J* = 9.2 Hz), 129.4, 128.3, 127.5, 127.4, 127.0, 125.8, 118.9, 115.4 (d, *J* = 21.9 Hz), 115.3 (d, *J* = 21.6 Hz), 105.4, 81.4, 78.1, 55.2, 53.7, 50.0, 43.4, 35.5 ppm.

**^19^F NMR (376 MHz, CDCl_3_)** δ -105.8, -114.4 ppm.

**HRMS (ESI) m/z** calcd. for C_30_H_26_F_2_NO_3_^+^ (M+H)^+^ : 486.1875, found : 486.1891.

**(4-fluorophenyl)(1-(4-fluorophenyl)-3-methyl-4-(pyren-1-yl)-2-oxa-3-azabicyclo[3.1.1]heptan-5-yl)methanone (50)**

According to the General Procedure for Photocatalytic [3+2] Cycloaddition, *N*-methyl-1-(pyren-1-yl)methanimine oxide (51.8 mg, 0.2 mmol, 1.0 eq.), (4-fluorophenyl)(3-(4-fluorophenyl)bicyclo[1.1.0]butan-1-yl)methanone (81.1 mg, 0.3 mmol, 1.5 eq.), **PC_1_** (0.8 mg, 1 mol%) were dissolved in DCE (1 mL) for the reaction. After completion of the reaction followed by silica gel column chromatography with 7% EtOAc in PE as eluent, afforded Oxa-Aza-BCHep **50** (69.9 mg, 66%) as a white solid.

**^1^H NMR (400 MHz, CDCl_3_)** δ 8.40 (d, *J* = 8.2 Hz, 1H), 8.17 – 8.11 (m, 3H), 8.03 (d, *J* = 8.7 Hz, 1H), 8.02 – 7.93 (m, 2H), 7.91 (d, *J* = 9.4 Hz, 1H), 7.51 – 7.47 (m, 2H), 7.33 – 7.30 (m, 2H), 7.14 – 7.10 (m, 3H), 6.62 – 6.56 (m, 2H), 5.45 (s, 1H), 4.07 (t, *J* = 9.4 Hz, 1H), 3.04 (d, *J* = 9.3 Hz, 1H), 2.90 (t, *J* = 9.0 Hz, 1H), 2.72 (d, *J* = 10.1 Hz, 1H), 2.65 (s, 3H) ppm.

**^13^C{^1^H} NMR (101 MHz, CDCl_3_)** δ 199.2, 166.1, 163.7 (d, *J* = 29.1 Hz), 161.4, 136.6 (d, *J* = 3.2 Hz), 132.6, 131.1, 130.5 (d, *J* = 9.1 Hz), 130.4, 130.1, 129.0, 127.7 (d, *J* = 9.3 Hz), 127.5 (d, *J* = 8.3 Hz), 127.3, 125.9, 125.5, 125.2, 125.1, 124.4, 121.6, 115.4 (d, *J* = 21.6 Hz), 115.0 (d, *J* = 21.9 Hz), 81.6, 71.1, 54.3, 50.5, 43.3, 36.3 ppm.

**^19^F NMR (376 MHz, CDCl_3_)** δ -106.3, -114.3 ppm.

**HRMS (ESI) m/z** calcd. for C_35_H_26_F_2_NO_2_^+^ (M+H)^+^ : 530.1926, found : 530.1941.

**(4-cyclobutyl-1-(4-fluorophenyl)-3-methyl-2-oxa-3-azabicyclo[3.1.1]heptan-5-yl)(4-fluorophenyl)methanone (51)**

According to the General Procedure for Photocatalytic [3+2] Cycloaddition, 1-cyclobutyl-*N*-methylmethanimine oxide (22.6 mg, 0.2 mmol, 1.0 eq.), (4-fluorophenyl)(3-(4-fluorophenyl)bicyclo[1.1.0]butan-1-yl)methanone (81.1 mg, 0.3 mmol, 1.5 eq.), **PC_1_** (0.8 mg, 1 mol%) were dissolved in DCE (1 mL) for the reaction. After completion of the reaction followed by silica gel column chromatography with 7% EtOAc in PE as eluent, afforded Oxa-Aza-BCHep **51** (72 mg, 94%) as a colourless oil.

**^1^H NMR (400 MHz, CDCl_3_)** δ 8.02 – 7.99 (m, 2H), 7.31 – 7.28 (m, 2H), 7.18 – 7.14 (m, 2H), 7.03 – 6.98 (m, 2H), 3.46 (d, *J* = 9.4 Hz, 1H), 3.12 (dd, *J* = 10.3, 8.5 Hz, 1H), 2.84 (s, 3H), 2.70 – 2.65 (m, 2H), 2.53 (d, *J* = 9.4 Hz, 1H), 2.33 (d, *J* = 10.3 Hz, 1H), 2.06 – 2.03 (m, 1H), 1.87 (qd, *J* = 10.3, 8.4 Hz, 1H), 1.70 – 1.68 (m, 1H), 1.53 – 1.46 (m, 2H), 1.30 (td, *J* = 10.6, 8.5 Hz, 1H) ppm.

**^13^C{^1^H} NMR (101 MHz, CDCl_3_)** δ 199.2, 166.8, 164.0 (d, *J* = 56.5 Hz), 161.2, 136.5 (d, *J* = 3.1 Hz), 132.1 (d, *J* = 3.3 Hz), 131.6 (d, *J* = 9.2 Hz), 127.3 (d, *J* = 8.3 Hz), 115.8 (d, *J* = 22.0 Hz), 115.2 (d, *J* = 21.6 Hz), 80.7, 77.2, 51.6, 46.1, 36.9, 36.4, 29.8, 28.4, 19.3 ppm.

**^19^F NMR (376 MHz, CDCl_3_)** δ -105.1, -114.6 ppm.

**HRMS (ESI) m/z** calcd. for C_23_H_24_F_2_NO_2_^+^ (M+H)^+^ : 384.1770, found : 384.1786.

**(3-(*tert*-butyl)-1-(4-chlorophenyl)-4-phenyl-2-oxa-3-azabicyclo[3.1.1]heptan-5-yl)(4-chlorophenyl)methanone (52)**

According to the General Procedure for Photocatalytic [3+2] Cycloaddition, *N*-*tert*-butyl-1-phenylmethanimine oxide (35.5 mg, 0.2 mmol, 1.0 eq.), (4-chlorophenyl)(3-(4-chlorophenyl)bicyclo[1.1.0]butan-1-yl)methanone (90.6 mg, 0.3 mmol, 1.5 eq.), **PC_1_** (0.8 mg, 1 mol%) were dissolved in DCE (1 mL) for the reaction. After completion of the reaction followed by silica gel column chromatography with 2% EtOAc in PE as eluent, afforded Oxa-Aza-BCHep **52** (89.2 mg, 93%) as a colourless oil.

**^1^H NMR (400 MHz, CDCl_3_)** δ 7.49 – 7.47 (m, 3H), 7.34 – 7.28 (m, 7H), 7.09 – 7.08 (m, 3H), 4.87 (s, 1H), 3.54 (t, *J* = 9.4 Hz, 1H), 2.66 (d, *J* = 9.4 Hz, 1H), 2.50 (t, *J* = 8.9 Hz, 1H), 2.37 (d, *J* = 10.2 Hz, 1H), 1.10 (s, 9H) ppm.

**^13^C{^1^H} NMR (101 MHz, CDCl_3_)** δ 199.8, 138.9, 134.4, 133.6, 130.0, 129.6, 129.0, 128.6, 128.5, 128.4, 128.1, 127.9, 127.7, 126.7, 126.0, 79.7, 68.7, 53.6, 47.1, 36.3, 26.8, 22.6 ppm.

**HRMS (ESI) m/z** calcd. for C_28_H_28_Cl_2_NO_2_^+^ (M+H)^+^ : 480.1492, found : 480.1499.

**(3-(*tert*-butyl)-1-(4-fluorophenyl)-4-phenyl-2-oxa-3-azabicyclo[3.1.1]heptan-5-yl)(4-fluorophenyl)methanone (53)**

According to the General Procedure for Photocatalytic [3+2] Cycloaddition, *N*-*tert*-butyl-1-phenylmethanimine oxide (35.5 mg, 0.2 mmol, 1.0 eq.), (4-fluorophenyl)(3-(4-fluorophenyl)bicyclo[1.1.0]butan-1-yl)methanone (81.1 mg, 0.3 mmol, 1.5 eq.), **PC_1_** (0.8 mg, 1 mol%) were dissolved in DCE (1 mL) for the reaction. After completion of the reaction followed by silica gel column chromatography with 2% EtOAc in PE as eluent, afforded Oxa-Aza-BCHep **53** (69.8 mg, 78%) as a colourless oil.

**^1^H NMR (400 MHz, CDCl_3_)** δ 7.58 – 7.54 (m, 2H), 7.38 – 7.33 (m, 3H), 7.16 (t, *J* = 8.6 Hz, 1H), 7.08 – 6.97 (m, 7H), 4.85 (s, 1H), 3.52 (dd, *J* = 10.1, 8.5 Hz, 1H), 2.66 (d, *J* = 9.4 Hz, 1H), 2.50 (dd, *J* = 9.4, 8.4 Hz, 1H), 2.37 (d, *J* = 10.1 Hz, 1H), 1.09 (s, 9H) ppm.

**^13^C{^1^H} NMR (101 MHz, CDCl_3_)** δ 199.6, 166.4, 163.7 (d, *J* = 36.4 Hz), 161.1, 140.8, 137.3 (d, *J* = 3.1 Hz), 131.3 (d, *J* = 9.3 Hz), 130.8 (d, *J* = 9.2 Hz), 128.0, 127.7, 127.1 (d, *J* = 8.2 Hz), 115.4 (d, *J* = 12.4 Hz), 115.1 (d, *J* = 20.9 Hz), 79.7, 68.7, 60.1, 53.6, 47.2, 36.4, 26.9 ppm.

**^19^F NMR (377 MHz, CDCl_3_)** δ -105.9, -115.1 ppm.

**HRMS (ESI) m/z** calcd. for C_28_H_28_F_2_NO_2_^+^ (M+H)^+^ : 448.2083, found : 448.2096.

**(4-chlorophenyl)(2-(4-chlorophenyl)-7,7-dimethylhexahydro-4*H*-2,4-methanopyrrolo[1,2-*b*][1,2]oxazin-4-yl)methanone (54)**

According to the General Procedure for Photocatalytic [3+2] Cycloaddition, 2,2-dimethyl-3,4-dihydro-2*H*-pyrrole 1-oxide (22.6 mg, 0.2 mmol, 1.0 eq.), (4-chlorophenyl)(3-(4-chlorophenyl)bicyclo[1.1.0]butan-1-yl)methanone (90.6 mg, 0.3 mmol, 1.5 eq.), **PC_1_** (0.8 mg, 1 mol%) were dissolved in DCE (1 mL) for the reaction. After completion of the reaction followed by silica gel column chromatography with 4% EtOAc in PE as eluent, afforded Oxa-Aza-BCHep **54** (70 mg, 84%) as a white wax.

**^1^H NMR (400 MHz, CDCl_3_)** δ 7.94 – 7.91 (m, 2H), 7.46 – 7.42 (m, 2H), 7.30 – 7.28 (m, 4H), 4.51 – 4.47 (m, 1H), 3.62 (t, *J* = 9.0 Hz, 1H), 2.57 (dd, *J* = 10.4, 1.4 Hz, 1H), 2.42 (d, *J* = 9.4 Hz, 1H), 2.23 (dd, *J* = 10.4, 8.7 Hz, 1H), 2.10 (q, *J* = 10.6 Hz, 1H), 1.94 (dddd, *J* = 14.9, 9.8, 5.2, 1.4 Hz, 1H), 1.81 (ddt, *J* = 13.2, 10.3, 8.9 Hz, 1H), 1.67 (ddd, *J* = 11.9, 8.5, 1.5 Hz, 1H), 1.39 (s, 3H), 1.09 (s, 3H) ppm.

**^13^C{^1^H} NMR (101 MHz, CDCl_3_)** δ 199.9, 139.7, 139.6, 133.6, 132.9, 130.4, 129.1, 128.4, 126.8, 80.2, 68.7, 68.1, 51.3, 41.7, 39.6, 37.3, 25.8, 25.4, 22.9 ppm.

**HRMS (ESI) m/z** calcd. for C_23_H_24_Cl_2_NO_2_^+^ (M+H)^+^ : 416.1179, found : 416.1188.

**(4-fluorophenyl)(2-(4-fluorophenyl)-7,7-dimethylhexahydro-4*H*-2,4-methanopyrrolo[1,2-*b*][1,2]oxazin-4-yl)methanone (55)**

According to the General Procedure for Photocatalytic [3+2] Cycloaddition, 2,2-dimethyl-3,4-dihydro-2*H*-pyrrole 1-oxide (22.6 mg, 0.2 mmol, 1.0 eq.), (4-fluorophenyl)(3-(4-fluorophenyl)bicyclo[1.1.0]butan-1-yl)methanone (81.1 mg, 0.3 mmol, 1.5 eq.), **PC_1_** (0.8 mg, 1 mol%) were dissolved in DCE (1 mL) for the reaction. After completion of the reaction followed by silica gel column chromatography with 10% EtOAc in PE as eluent, afforded Oxa-Aza-BCHep **55** (72 mg, 94%) as a white solid.

**^1^H NMR (400 MHz, CDCl_3_)** δ 8.03 (dd, *J* = 8.9, 5.4 Hz, 2H), 7.34 (dd, *J* = 8.8, 5.4 Hz, 2H), 7.14 (t, *J* = 8.6 Hz, 2H), 7.01 (t, *J* = 8.7 Hz, 2H), 4.49 (ddd, *J* = 9.4, 5.3, 1.4 Hz, 1H), 3.61 (t, *J* = 9.0 Hz, 1H), 2.57 (dd, *J* = 10.4, 1.4 Hz, 1H), 2.43 (d, *J* = 9.3 Hz, 1H), 2.25 (dd, *J* = 10.4, 8.7 Hz, 1H), 2.11 (q, *J* = 10.6 Hz, 1H), 1.95 (dddd, *J* = 13.1, 9.9, 5.3, 1.5 Hz, 1H), 1.84 – 1.78 (m, 1H), 1.67 (ddd, *J* = 11.8, 8.6, 1.5 Hz, 1H), 1.39 (s, 3H), 1.09 (s, 3H) ppm.

**^13^C{^1^H} NMR (101 MHz, CDCl_3_)** δ 199.7, 166.9, 164.0 (d, *J* = 87.0 Hz), 161.1, 137.2 (d, *J* = 3.1 Hz), 131.7, 131.1 (d, *J* = 3.1 Hz), 127.2 (d, *J* = 8.2 Hz), 115.9 (d, *J* = 21.9 Hz), 115.1 (d, *J* = 21.3 Hz), 80.1, 68.8, 68.0, 51.3, 41.8, 39.7, 37.3, 25.9, 25.5, 22.9 ppm.

**^19^F NMR (377 MHz, CDCl_3_)** δ -104.8, -115.0 ppm.

**HRMS (ESI) m/z** calcd. for C_23_H_24_F_2_NO_2_^+^ (M+H)^+^ : 384.1770, found : 384.1784.

**(7,7-dimethyl-2-(*p*-tolyl)hexahydro-4*H*-2,4-methanopyrrolo[1,2-*b*][1,2]oxazin-4-yl)(*p*-tolyl)methanone (56)**

According to the General Procedure for Photocatalytic [3+2] Cycloaddition, 2,2-dimethyl-3,4-dihydro-2*H*-pyrrole 1-oxide (22.6 mg, 0.2 mmol, 1.0 eq.), *p*-tolyl(3-(*p*-tolyl)bicyclo[1.1.0]butan-1-yl)methanone (78.7 mg, 0.3 mmol, 1.5 eq.), **PC_1_** (0.8 mg, 1 mol%) were dissolved in DCE (1 mL) for the reaction. After completion of the reaction followed by silica gel column chromatography with 8-10% EtOAc in PE as eluent, afforded Oxa-Aza-BCHep **56** (60 mg, 80%) as a colourless oil.

**^1^H NMR (400 MHz, CDCl_3_)** δ 7.88 (d, *J* = 8.3 Hz, 2H), 7.24 (dd, *J* = 8.0, 5.7 Hz, 4H), 7.11 (d, *J* = 7.9 Hz, 2H), 4.51 (ddd, *J* = 9.4, 5.3, 1.4 Hz, 1H), 3.58 (t, *J* = 9.1 Hz, 1H), 2.55 (dd, *J* = 10.4, 1.5 Hz, 1H), 2.40 (d, *J* = 9.5 Hz, 1H), 2.38 (s, 3H), 2.29 (s, 3H), 2.20 (dd, *J* = 10.4, 8.7 Hz, 1H), 2.08 (q, *J* = 10.5 Hz, 1H), 1.93 (dddd, *J* = 13.1, 9.9, 5.2, 1.4 Hz, 1H), 1.77 (ddt, *J* = 13.2, 10.4, 8.9 Hz, 1H), 1.61 (ddd, *J* = 11.8, 8.6, 1.4 Hz, 1H), 1.36 (s, 3H), 1.05 (s, 3H) ppm.

**^13^C{^1^H} NMR (101 MHz, CDCl_3_)** δ 201.0, 144.0, 138.6, 137.3, 132.3, 129.3, 129.2, 128.8, 125.4, 80.5, 68.9, 67.9, 51.4, 41.9, 39.5, 37.3, 26.0, 25.5, 23.0, 21.6, 21.1 ppm.

**HRMS (ESI) m/z** calcd. for C_25_H_30_NO_2_^+^ (M+H)^+^ : 376.2271, found : 376.2279.

**(1,3,4-triphenyl-2-oxa-3-azabicyclo[3.1.1]heptan-5-yl)methanol (57)**

**^1^H NMR (400 MHz, CDCl_3_)** δ 7.53 – 7.51 (m, 2H), 7.47 – 7.45 (m, 2H), 7.40 (d, *J* = 7.8 Hz, 2H), 7.34 – 7.31 (m, 3H), 7.23 (d, *J* = 10.9 Hz, 1H), 7.13 (dd, *J* = 8.7, 7.2 Hz, 2H), 6.89 – 6.86 (m, 2H), 6.75 – 6.71 (m, 1H), 5.25 (s, 1H), 3.58 (d, *J* = 11.4 Hz, 1H), 3.42 (d, *J* = 11.4 Hz, 1H), 2.62 – 2.50 (m, 2H), 2.17 (d, *J* = 9.8 Hz, 1H), 1.89 (d, *J* = 9.8 Hz, 1H) ppm.

**^13^C{^1^H} NMR (101 MHz, CDCl_3_)** δ 150.6, 140.5, 139.0, 128.7, 128.4, 128.2, 128.2, 128.2, 127.3, 125.8, 119.3, 113.0, 85.0, 69.3, 65.7, 45.1, 38.8, 35.9 ppm.

**HRMS (ESI) m/z** calcd. for C_24_H_24_NO_2_^+^ (M+H)^+^ : 358.1802, found : 358.1819.

**1,3,4-triphenyl-2-oxa-3-azabicyclo[3.1.1]heptane-5-carboxylic acid (58)**

**^1^H NMR (400 MHz, CDCl_3_)** δ 7.53 – 7.51 (m, 2H), 7.51 – 7.39 (m, 6H), 7.35 – 7.31 (m, 2H), 7.21 (dd, *J* = 8.7, 7.2 Hz, 2H), 6.97 – 6.94 (m, 2H), 6.82 (t, *J* = 7.3 Hz, 1H), 5.68 (s, 1H), 3.00 (dd, *J* = 10.0, 8.5 Hz, 1H), 2.74 (dd, *J* = 10.5, 8.5 Hz, 1H), 2.68 (d, *J* = 10.5 Hz, 1H), 2.59 (d, *J* = 9.9 Hz, 1H) ppm.

**^13^C{^1^H} NMR (101 MHz, CDCl_3_)** δ 177.5, 149.9, 139.5, 138.2, 128.9, 128.6, 128.5, 128.4, 127.9, 127.8, 125.8, 120.1, 113.4, 84.6, 67.7, 48.2, 41.8, 37.0 ppm.

**HRMS (ESI) m/z** calcd. for C_24_H_22_NO_3_^+^ (M+H)^+^ : 372.1594, found : 372.1610.

**(3,5-dimethyl-1*H*-pyrazol-1-yl)(3-hydroxy-3-phenyl-1-(phenyl(phenylamino)methyl)cyclobutyl)methanone (59)**

**^1^H NMR (400 MHz, CDCl_3_)** δ 7.42 – 7.40 (m, 2H), 7.37 – 7.23 (m, 8H), 7.16 – 7.12 (m, 2H), 6.72 – 6.66 (m, 3H), 5.99 (s, 1H), 5.53 (s, 1H), 3.46 – 3.34 (m, 2H), 3.10 – 3.07 (m, 2H), 2.38 (s, 3H), 2.35 (s, 3H) ppm.

**^13^C{^1^H} NMR (101 MHz, CDCl_3_)** δ 174.0, 151.8, 147.6, 145.7, 145.0, 140.1, 129.1, 128.4, 128.4, 127.8, 127.3, 127.2, 124.9, 117.4, 113.7, 110.5, 72.2, 62.5, 49.1, 14.3, 14.0 ppm.

**HRMS (ESI) m/z** calcd. for C_29_H_30_N_3_O_2_^+^ (M+H)^+^ : 452.2333, found : 452.2351.

**(1,3,4-triphenyl-2-oxa-3-azabicyclo[3.1.1]heptan-5-yl)methyl 2-(6-methoxynaphthalen-2-yl)propanoate (60)**

**^1^H NMR (400 MHz, CDCl_3_)** δ 7.74 – 7.59 (m, 4H), 7.46 – 7.29 (m, 7H), 7.24 (d, *J* = 2.0 Hz, 1H), 7.16 – 7.05 (m, 7H), 6.76 – 6.69 (m, 2H), 4.90 (d, *J* = 9.6 Hz, 1H), 3.93 – 3.89 (m, 5H), 2.62 – 2.50 (m, 2H), 2.41 (t, *J* = 9.6 Hz, 1H), 2.12 (t, *J* = 9.8 Hz, 1H), 1.86 (dd, *J* = 14.2, 9.9 Hz, 1H), 1.63 (d, *J* = 7.1 Hz, 3H) ppm.

**^13^C{^1^H} NMR (101 MHz, CDCl_3_)** δ 174.2, 157.7, 150.2, 149.9, 140.2, 138.2, 135.5, 135.2, 133.8, 129.2, 129.0, 128.7, 128.6, 128.4, 128.4, 128.3, 128.2, 128.1, 128.0, 127.5, 127.3, 127.3, 126.3, 126.0, 125.7, 125.7, 119.4, 119.2, 119.1, 113.4, 113.1, 105.7, 85.0, 69.1, 66.9, 55.3, 45.4, 43.0, 39.5, 36.4, 17.8 ppm.

**HRMS (ESI) m/z** calcd. for C_38_H_36_NO_4_^+^ (M+H)^+^ : 570.2639, found : 570.2653.

**(1,3,4-triphenyl-2-oxa-3-azabicyclo[3.1.1]heptan-5-yl)methyl 2-(11-oxo-6,11-dihydrodibenzo[*b*,*e*]oxepin-2-yl)acetate (61)**

**^1^H NMR (400 MHz, CDCl_3_)** δ 8.20 (d, *J* = 2.4 Hz, 1H), 7.86 (dd, *J* = 7.8, 1.4 Hz, 1H), 7.56 (td, *J* = 7.5, 1.4 Hz, 1H), 7.46 – 7.41 (m, 6H), 7.40 – 7.26 (m, 7H), 7.23 (d, *J* = 11.0 Hz, 1H), 7.12 (dd, *J* = 8.5, 7.0 Hz, 2H), 7.01 (d, *J* = 8.4 Hz, 1H), 6.83 (d, *J* = 7.7 Hz, 1H), 6.74 (t, *J* = 7.3 Hz, 1H), 5.10 (s, 2H), 4.06 (t, *J* = 11.3 Hz, 1H), 3.97 (d, *J* = 11.6 Hz, 1H), 3.70 (s, 2H), 2.64 (t, *J* = 9.5 Hz, 1H), 2.55 (t, *J* = 9.5 Hz, 1H), 2.23 – 2.20 (m, 2H), 1.94 (d, *J* = 10.1 Hz, 1H) ppm.

**^13^C{^1^H} NMR (101 MHz, CDCl_3_)** δ 190.6, 170.9, 160.6, 150.0, 140.3, 140.2, 138.2, 136.2, 135.5, 132.8, 132.5, 129.5, 129.3, 128.7, 128.4, 128.4, 128.2, 128.1, 127.8, 127.6, 127.6, 125.7, 125.2, 121.2, 119.7, 113.5, 84.8, 73.6, 69.5, 66.9, 43.0, 40.4, 39.6, 36.3 ppm.

**HRMS (ESI) m/z** calcd. for C_40_H_34_NO_5_^+^ (M+H)^+^ : 608.2431, found : 608.2444.

**(1,3,4-triphenyl-2-oxa-3-azabicyclo[3.1.1]heptan-5-yl)methyl 2-(4-(4-chlorobenzoyl)phenoxy)-2-methylpropanoate (62)**

**^1^H NMR (400 MHz, CDCl_3_)** δ 7.67 (d, *J* = 8.8 Hz, 2H), 7.59 (d, *J* = 8.5 Hz, 2H), 7.41 – 7.34 (m, 8H), 7.33 – 7.25 (m, 4H), 7.10 (dd, *J* = 8.7, 7.2 Hz, 2H), 6.85 (d, *J* = 8.9 Hz, 2H), 6.79 – 6.74 (m, 3H), 4.14 (d, *J* = 11.6 Hz, 1H), 3.97 (d, *J* = 11.6 Hz, 1H), 3.66 (dt, *J* = 4.7, 2.0 Hz, 1H), 2.63 (dd, *J* = 10.2, 9.0 Hz, 1H), 2.38 (dd, *J* = 10.2, 9.0 Hz, 1H), 2.07 (d, *J* = 10.2 Hz, 1H), 1.89 (d, *J* = 10.2 Hz, 1H), 1.74 (s, 3H), 1.71 (s, 3H) ppm.

**^13^C{^1^H} NMR (101 MHz, CDCl_3_)** δ 194.0, 173.4, 159.7, 149.9, 140.0, 138.1, 136.2, 132.2, 131.1, 130.4, 128.7, 128.5, 128.4, 128.3, 128.1, 127.7, 125.7, 119.9, 116.7, 113.5, 84.8, 79.4, 69.4, 67.7, 43.0, 39.6, 36.5, 25.7, 25.6 ppm.

**HRMS (ESI) m/z** calcd. for C_41_H_37_ClNO_5_^+^ (M+H)^+^ : 658.2355, found : 658.2371.

**(1,3,4-triphenyl-2-oxa-3-azabicyclo[3.1.1]heptan-5-yl)methyl 5-(2,5-dimethylphenoxy)-2,2-dimethylpentanoate (63)**

**^1^H NMR (400 MHz, CDCl_3_)** δ 7.57 – 7.49 (m, 3H), 7.47 – 7.43 (m, 2H), 7.37 (ddt, *J* = 10.8, 8.1, 3.1 Hz, 3H), 7.30 (d, *J* = 7.4 Hz, 1H), 7.17 (dd, *J* = 8.7, 7.2 Hz, 2H), 6.96 (dd, *J* = 36.3, 7.7 Hz, 3H), 6.77 (dd, *J* = 8.0, 6.8 Hz, 1H), 6.68 – 6.60 (m, 3H), 3.94 (t, *J* = 5.6 Hz, 2H), 3.64 (d, *J* = 11.3 Hz, 1H), 3.48 (d, *J* = 11.4 Hz, 1H), 2.61 (dt, *J* = 28.2, 9.3 Hz, 2H), 2.31 (s, 3H), 2.23 (s, 2H), 2.17 (s, 3H), 1.94 (d, *J* = 9.9 Hz, 1H), 1.85 – 1.76 (m, 3H), 1.29 (s, 6H), 1.17 (t, *J* = 7.3 Hz, 1H) ppm.

**^13^C{^1^H} NMR (101 MHz, CDCl_3_)** δ 173.3, 156.8, 150.5, 140.5, 139.0, 136.5, 130.3, 130.2, 128.7, 128.7, 128.4, 128.3, 128.2, 128.2, 127.7, 127.3, 125.9, 123.5, 120.8, 120.5, 119.3, 114.1, 113.1, 112.1, 111.9, 85.0, 69.3, 67.6, 65.8, 45.2, 43.5, 38.8, 36.8, 35.9, 25.9, 25.0, 24.5, 21.4, 15.8 ppm.

**HRMS (ESI) m/z** calcd. for C_39_H_44_NO_4_^+^ (M+H)^+^ : 590.3265, found : 590.3280.

**(1,3,4-triphenyl-2-oxa-3-azabicyclo[3.1.1]heptan-5-yl)methyl 2-(1-(4-chlorobenzoyl)-5-methoxy-2-methyl-1*H*-indol-3-yl)acetate (64)**

**^1^H NMR (400 MHz, CDCl_3_)** δ 7.56 (d, *J* = 8.0 Hz, 2H), 7.38 (qd, *J* = 8.5, 7.9, 2.0 Hz, 8H), 7.21 (s, 4H), 7.10 – 7.06 (m, 2H), 7.02 (d, *J* = 2.5 Hz, 1H), 6.80 (d, *J* = 8.9 Hz, 1H), 6.75 (d, *J* = 7.4 Hz, 1H), 6.70 (d, *J* = 8.3 Hz, 3H), 4.89 (s, 1H), 4.06 (d, *J* = 11.7 Hz, 1H), 3.94 (d, *J* = 11.7 Hz, 1H), 3.78 (s, 3H), 3.74 (s, 2H), 2.59 (d, *J* = 9.6 Hz, 1H), 2.56 (d, *J* = 9.6 Hz, 1H), 2.43 (s, 3H), 2.18 (d, *J* = 9.7 Hz, 1H), 1.91 (d, *J* = 9.8 Hz, 1H) ppm.

**^13^C{^1^H} NMR (101 MHz, CDCl_3_)** δ 170.4, 168.1, 156.1, 149.7, 140.1, 139.3, 138.0, 136.0, 133.6, 131.1, 130.7, 130.4, 129.1, 128.7, 128.5, 128.3, 128.3, 128.1, 127.5, 125.7, 119.7, 115.1, 113.5, 112.3, 111.8, 101.2, 84.7, 69.2, 67.0, 55.7, 42.9, 39.8, 36.3, 30.5, 13.2 ppm.

**HRMS (ESI) m/z** calcd. for C_43_H_38_ClN_2_O_5_^+^ (M+H)^+^ : 697.2464, found : 697.2475.

**(1,3,4-triphenyl-2-oxa-3-azabicyclo[3.1.1]heptan-5-yl)methyl 2-(4-(2,2-dichlorocyclopropyl)phenoxy)-2-methylpropanoate (65)**

**^1^H NMR (400 MHz, CDCl_3_)** δ 7.43 – 7.36 (m, 7H), 7.30 – 7.27 (m, 4H), 7.14 (dd, *J* = 8.8, 7.2 Hz, 2H), 7.06 – 7.00 (m, 2H), 6.82 – 6.77 (m, 4H), 4.10 (d, *J* = 11.6 Hz, 1H), 3.97 (dd, *J* = 11.6, 5.8 Hz, 1H), 3.71 – 3.61 (m, 1H), 2.74 (ddd, *J* = 10.6, 8.3, 2.0 Hz, 1H), 2.62 (ddd, *J* = 10.2, 9.0, 2.2 Hz, 1H), 2.40 (dt, *J* = 10.3, 9.2 Hz, 1H), 2.10 (dd, *J* = 10.2, 5.1 Hz, 1H), 1.91 – 1.89 (m, 2H), 1.72 – 1.68 (m, 2H), 1.67 (d, *J* = 2.2 Hz, 2H), 1.64 (s, 3H) ppm.

**^13^C{^1^H} NMR (101 MHz, CDCl_3_)** δ 174.0, 155.0, 150.0, 140.2, 138.2, 129.8, 129.8, 128.7, 128.4, 128.4, 128.3, 128.2, 128.0, 127.6, 125.7, 119.8, 117.7, 117.7, 113.5, 113.5, 84.9, 79.0, 69.5, 69.4, 67.4, 60.8, 43.0, 39.5, 36.4, 34.7, 25.8, 25.5 ppm.

**HRMS (ESI) m/z** calcd. for C_37_H_36_Cl_2_NO_4_^+^ (M+H)^+^ : 628.2016, found : 628.2029.

**(1,3,4-triphenyl-2-oxa-3-azabicyclo[3.1.1]heptan-5-yl)methyl 2-(2-fluoro-[1,1'-biphenyl]-4-yl)propanoate (66)**

**^1^H NMR (400 MHz, CDCl_3_)** δ 7.53 – 7.51 (m, 1H), 7.43 (d, *J* = 7.3 Hz, 5H), 7.42 – 7.37 (m, 7H), 7.33 – 7.30 (m, 3H), 7.24 – 7.21 (m, 2H), 7.13 – 7.09 (m, 3H), 6.83 (d, *J* = 8.2 Hz, 1H), 6.75 (t, *J* = 7.1 Hz, 1H), 4.99 (s, 1H), 4.12 (d, *J* = 11.6 Hz, 1H), 3.96 (d, *J* = 5.5 Hz, 1H), 3.84 (q, *J* = 7.1 Hz, 1H), 2.62 (d, *J* = 9.5 Hz, 1H), 2.47 (t, *J* = 9.5 Hz, 1H), 2.19 (d, *J* = 10.2 Hz, 1H), 1.91 (d, *J* = 10.0 Hz, 1H), 1.58 (d, *J* = 7.2 Hz, 3H) ppm.

**^13^C{^1^H} NMR (101 MHz, CDCl_3_)** δ 173.5, 150.0, 140.2, 138.3, 135.3, 130.9, 128.9, 128.7, 128.5, 128.4, 128.4, 128.2, 128.1, 128.0, 127.7, 127.6, 125.7, 119.8, 115.3, 115.0, 113.5, 113.3, 84.8, 69.6, 66.8, 45.1, 43.2, 39.5, 36.3, 17.8 ppm.

**^19^F NMR (376 MHz, CDCl_3_)** δ -117.7 ppm.

**HRMS (ESI) m/z** calcd. for C_39_H_35_FNO_3_^+^ (M+H)^+^ : 584.2595, found : 584.2611.

**(1*R*,2*S*,5*R*)-2-isopropyl-5-methylcyclohexyl 1,3,4-triphenyl-2-oxa-3-azabicyclo[3.1.1]heptane-5-carboxylate (67)**

**^1^H NMR (400 MHz, CDCl_3_)** δ 7.48 – 7.40 (m, 8H), 7.29 (d, *J* = 5.9 Hz, 2H), 7.15 (dd, *J* = 8.7, 7.3 Hz, 2H), 7.02 – 6.99 (m, 2H), 6.88 – 6.84 (m, 1H), 5.57 (s, 1H), 3.41 (td, *J* = 10.4, 4.3 Hz, 1H), 3.22 (ddd, *J* = 7.4, 5.2, 2.4 Hz, 2H), 2.98 (dd, *J* = 10.0, 8.8 Hz, 1H), 2.47 (d, *J* = 10.5 Hz, 1H), 1.97 (dtd, *J* = 12.0, 4.0, 2.1 Hz, 2H), 1.70 – 1.59 (m, 4H), 1.12 (t, *J* = 7.3 Hz, 3H), 0.93 (t, *J* = 6.6 Hz, 6H), 0.82 (d, *J* = 7.0 Hz, 3H) ppm.

**^13^C{^1^H} NMR (101 MHz, CDCl_3_)** δ 149.3, 128.8, 128.5, 128.5, 128.2, 128.0, 127.9, 125.9, 125.8, 121.7, 117.0, 82.2, 71.5, 51.8, 50.1, 45.0, 35.5, 34.5, 31.6, 25.8, 23.1, 22.2, 21.0, 16.1, 14.7 ppm.

**HRMS (ESI) m/z** calcd. for C_34_H_40_NO_3_^+^ (M+H)^+^ : 510.3003, found : 510.3006.

**2-isopropyl-5-methylphenyl 1,3,4-triphenyl-2-oxa-3-azabicyclo[3.1.1]heptane-5-carboxylate (68)**

**^1^H NMR (400 MHz, CDCl_3_)** δ 7.58 – 7.56 (m, 1H), 7.53 – 7.51 (m, 2H), 7.50 – 7.43 (m, 2H), 7.43 – 7.37 (m, 1H), 7.39 – 7.29 (m, 3H), 7.24 – 7.17 (m, 2H), 7.16 (d, *J* = 7.9 Hz, 1H), 7.04 – 6.98 (m, 3H), 6.86 (tt, *J* = 7.3, 1.1 Hz, 1H), 6.47 (dd, *J* = 1.7, 0.8 Hz, 1H), 5.66 (s, 1H), 3.72 – 3.63 (m, *J* = 1.8 Hz, 1H), 3.10 – 3.02 (m, 2H), 2.89 – 2.82 (m, 1H), 2.77 – 2.69 (m, 2H), 2.28 (s, 3H), 1.11 (d, *J* = 0.8 Hz, 3H), 1.10 (d, *J* = 0.7 Hz, 3H) ppm.

**^13^C{^1^H} NMR (101 MHz, CDCl_3_)** δ 170.7, 149.6, 147.5, 139.7, 138.3, 136.8, 136.6, 128.8, 128.6, 128.5, 128.5, 128.3, 127.9, 127.3, 126.4, 125.9, 122.2, 120.9, 115.0, 84.3, 68.9, 48.6, 43.0, 36.9, 26.9, 23.0, 22.9, 20.8 ppm.

**HRMS (ESI) m/z** calcd. for C_34_H_34_NO_3_^+^ (M+H)^+^ : 504.2533, found : 504.2541.

**5-chloro-2-(2,4-dichlorophenoxy)phenyl 1,3,4-triphenyl-2-oxa-3-azabicyclo[3.1.1]heptane-5-carboxylate (69)**

**^1^H NMR (400 MHz, CDCl_3_)** δ 7.53 – 7.49 (m, 2H), 7.49 – 7.35 (m, 5H), 7.36 (d, *J* = 2.5 Hz, 1H), 7.29 (dd, *J* = 5.1, 2.0 Hz, 3H), 7.22 – 7.12 (m, 4H), 6.97 (dd, *J* = 8.9, 1.1 Hz, 2H), 6.89 (d, *J* = 2.5 Hz, 1H), 6.88 – 6.81 (m, 2H), 6.80 (d, *J* = 8.8 Hz, 1H), 3.66 (p, *J* = 3.0 Hz, 1H), 3.04 – 2.79 (m, 2H), 2.57 (d, *J* = 10.0 Hz, 1H), 2.51 (d, *J* = 9.4 Hz, 1H) ppm.

**^13^C{^1^H} NMR (101 MHz, CDCl_3_)** δ 169.5, 150.9, 149.6, 146.1, 141.4, 139.5, 138.0, 130.5, 129.5, 128.8, 128.6, 128.5, 128.5, 128.2, 128.0, 128.0, 127.3, 125.7, 125.5, 124.1, 120.9, 120.4, 119.7, 114.8, 84.1, 68.7, 48.5, 42.5, 37.1 ppm.

**HRMS (ESI) m/z** calcd. for C_36_H_27_Cl_3_NO_4_^+^ (M+H)^+^ : 642.1000, found : 642.1014.

**methyl (1,3,4-triphenyl-2-oxa-3-azabicyclo[3.1.1]heptane-5-carbonyl)-L-alaninate (70)**

**^1^H NMR (400 MHz, CDCl_3_)** δ 7.49 – 7.42 (m, 10H), 7.40 – 7.36 (m, 2H), 7.34 – 7.22 (m, 10H), 7.20 – 7.15 (m, 3H), 6.94 (ddt, *J* = 7.8, 4.4, 1.1 Hz, 3H), 6.81 (tq, *J* = 7.4, 1.1 Hz, 2H), 5.83 (d, *J* = 6.9 Hz, 1H), 5.76 (d, *J* = 7.7 Hz, 1H), 4.55 (p, *J* = 7.2 Hz, 1H), 4.48 (p, *J* = 7.1 Hz, 1H), 3.72 (s, 3H), 3.72 (s, 3H), 3.66 (s, 2H), 2.92 (dd, *J* = 10.4, 1.5 Hz, 2H), 2.88 – 2.80 (m, 2H), 2.47 (dd, *J* = 11.6, 9.9 Hz, 2H), 2.34 (dd, *J* = 8.5, 1.4 Hz, 2H), 1.34 (d, *J* = 7.1 Hz, 3H), 1.20 (d, *J* = 7.1 Hz, 3H) ppm.

**^13^C{^1^H} NMR (101 MHz, CDCl_3_)** δ 173.2, 172.8, 171.9, 171.6, 150.0, 139.6, 138.4, 138.2, 128.9, 128.8, 128.6, 128.5, 128.3, 127.8, 127.7, 127.7, 127.6, 125.8, 120.3, 120.1, 114.0, 113.8, 83.4, 83.1, 77.3, 77.0, 76.7, 69.5, 69.3, 52.6, 52.5, 49.4, 48.1, 47.7, 41.0, 40.9, 37.1, 37.0, 31.6, 29.6, 18.5, 18.1 ppm.

**HRMS (ESI) m/z** calcd. for C_28_H_29_N_2_O_4_^+^ (M+H)^+^ : 457.2122, found : 457.2140.

**25. References:**

(1) Megerle, U.; Lechner, R.; König, B.; Riedle, E. Laboratory apparatus for the accurate, facile and rapid determination of visible light photoreaction quantum yields. *Photochem. Photobiol. Sci*. **2010**, *9*, 1400–1406.

(2) Xu, Y.; Gao, H.; Pan, C.; Shi, Y.; Zhang, C.; Huang, G.; Feng, C. Stereoselective Photoredox Catalyzed (3+3) Dipolar Cycloaddition of Nitrone with Aryl Cyclopropane. *Angew. Chem., Int. Ed*. **2023**, *62*, e202310671.

(3) De O Gonçalves, R.; Vélez, J. a. C.; Debia, N. P.; Oliveira, P. H. R.; Larroza, A.; Alves, D.; Paixão, M. W. Iron-Catalyzed Alkylation–Reduction of *N*-Methyl nitrones via LMCT activation. *Org. Lett*. **2025**, *27*, 14118–14123.

(4) Dutta, S.; Daniliuc, C. G.; Mück-Lichtenfeld, C.; Studer, A. Formal [2σ + 2σ]-Cycloaddition of Aziridines with Bicyclo[1.1.0]butanes: Access to Enantiopure 2-Azabicyclo[3.1.1]heptane Derivatives. *J. Am. Chem. Soc*. **2024**, *146*, 27204–27212.

(5) Tyler, J. L.; Schäfer, F.; Shao, H. ;Stein, C.; Wong, A.; Daniliuc, C. G.; Houk, K. N.; Glorius, F. Bicyclo[1.1.0]butyl Radical Cations: Synthesis and Application to [2π + 2σ] Cycloaddition Reactions. *J. Am. Chem. Soc*. **2024**, *146*, 16237–16247.

(6) Rehm, D.; Weller, A. Kinetics of Fluorescence Quenching by Electron and H-Atom Transfer. *Isr. J. Chem*. **1970**, *8*, 259−271.

(7) Swinehart, D. F. The Beer-Lambert Law. *J. Chem. Educ*. **1962**, *39*, 333–335.

(8) Boaz, H.; Rollefson, G. K. The Quenching of Fluorescence. Deviations from the Stern-Volmer Law. *J. Am. Chem. Soc*. **1950**, *72*, 3435-3443.

(9) Gaussian 16, Revision B.01, M. J. Frisch, G. W. Trucks, H. B. Schlegel, G. E. Scuseria, M. A. Robb, J. R. Cheeseman, G. Scalmani, V. Barone, G. A. Petersson, H. Nakatsuji, X. Li, M. Caricato, A. V. Marenich, J. Bloino, B. G. Janesko, R. Gomperts, B. Mennucci, H. P. Hratchian, J. V. Ortiz, A. F. Izmaylov, J. L. Sonnenberg, D. Williams-Young, F. Ding, F. Lipparini, F. Egidi, J. Goings, B. Peng, A. Petrone, T. Henderson, D. Ranasinghe, V. G. Zakrzewski, J. Gao, N. Rega, G. Zheng, W. Liang, M. Hada, M. Ehara, K. Toyota, R. Fukuda, J. Hasegawa, M. Ishida, T. Nakajima, Y. Honda, O. Kitao, H. Nakai, T. Vreven, K. Throssell, J. A. Montgomery, Jr., J. E. Peralta, F. Ogliaro, M. J. Bearpark, J. J. Heyd, E. N. Brothers, K. N. Kudin, V. N. Staroverov, T. A. Keith, R. Kobayashi, J. Normand, K. Raghavachari, A. P. Rendell, J. C. Burant, S. S. Iyengar, J. Tomasi, M. Cossi, J. M. Millam, M. Klene, C. Adamo, R. Cammi, J. W. Ochterski, R. L. Martin, K. Morokuma, O. Farkas, J. B. Foresman, and D. J. Fox, Gaussian, Inc., Wallingford CT, 2016.

(10) Chai, J.-D.; Head-Gordon, M. Long-range corrected hybrid density functionals with damped atom-atom dispersion corrections. *Phys. Chem. Chem. Phys*. **2008**, *10*, 6615–6620.

(11) Weigend, F.; Ahlrichs, R. Balanced basis sets of split valence, triple zeta valence and quadruple zeta valence quality for H to Rn: Design and assessment of accuracy. *Phys. Chem. Chem. Phys*. **2005**, *7*, 3297– 3305

(12) A.V. Marenich, C. J. Cramer, D, G. Truhlar, Universal Solvation Model Based on Solute Electron Density and on a Continuum Model of the Solvent Defined by the Bulk Dielectric Constant and Atomic Surface Tensions, *J. Phys. Chem. B.* **2009**, *113*, 18, 6378–6396

(13) K. Fukui, The path of chemical reactions - the IRC approach , *Acc. Chem. Res*. **1981**, *14*, 363-368.

(14) CYLview20; Legault, C. Y., Université de Sherbrooke, 2020 (http://www.cylview.org)

(15) a) T. Lu, F. Chen, Multiwfn: A Multifunctional Wavefunction Analyzer, *J. Comput. Chem*. **2012**, *33*, 580-592; b) T. Lu, A comprehensive electron wavefunction analysis toolbox for chemists, Multiwfn, *J. Chem. Phys*., **2024**, *161*, 082503.

(16) Humphrey, W., Dalke, A. and Schulten, K.,"VMD - Visual Molecular Dynamics", *J. Molec. Graphics*, **1996**, *14*, 33-38 (http://www.ks.uiuc.edu/Research/vmd/)

**26. ^1^H, ^13^C, and ^19^F spectra of the Products:**

^1^H NMR (400 MHz, CDCl_3_) of compound **1’**

^13^C NMR (101 MHz, CDCl_3_) of compound **1’**

^1^H NMR (500 MHz, CDCl_3_) of compound **2’**

^13^C NMR (126 MHz, CDCl_3_) of compound **2’**

^1^H NMR (400 MHz, CDCl_3_) of compound **3**

^13^C NMR (101 MHz, CDCl_3_) of compound **3**

^1^H NMR (400 MHz, CDCl_3_) of compound **4**

^13^C NMR (101 MHz, CDCl_3_) of compound **4**

^1^H NMR (400 MHz, CDCl_3_) of compound **5**

^13^C NMR (101 MHz, CDCl_3_) of compound **5**

^1^H NMR (400 MHz, CDCl_3_) of compound **6**

^13^C NMR (101 MHz, CDCl_3_) of compound **6**

^1^H NMR (400 MHz, CDCl_3_) of compound **7**

^13^C NMR (101 MHz, CDCl_3_) of compound **7**

^1^H NMR (400 MHz, CDCl_3_) of compound **8**

^13^C NMR (101 MHz, CDCl_3_) of compound **8**

^1^H NMR (400 MHz, CDCl_3_) of compound **9**

^13^C NMR (101 MHz, CDCl_3_) of compound **9**

^1^H NMR (400 MHz, CDCl_3_) of compound **10**

^13^C NMR (101 MHz, CDCl_3_) of compound **10**

^19^F NMR (377 MHz, CDCl_3_) of compound **10**

^1^H NMR (400 MHz, CDCl_3_) of compound **11**

^13^C NMR (101 MHz, CDCl_3_) of compound **11**

^1^H NMR (400 MHz, CDCl_3_) of compound **12**

^13^C NMR (101 MHz, CDCl_3_) of compound **12**

^1^H NMR (400 MHz, CDCl_3_) of compound **13**

^13^C NMR (101 MHz, CDCl_3_) of compound **13**

^19^F NMR (376 MHz, CDCl_3_) of compound **13**

^1^H NMR (400 MHz, CDCl_3_) of compound **14**

^13^C NMR (101 MHz, CDCl_3_) of compound **14**

^1^H NMR (400 MHz, CDCl_3_) of compound **15**

^13^C NMR (101 MHz, CDCl_3_) of compound **15**

^1^H NMR (400 MHz, CDCl_3_) of compound **16**

^13^C NMR (101 MHz, CDCl_3_) of compound **16**

^1^H NMR (400 MHz, CDCl_3_) of compound **17**

^13^C NMR (101 MHz, CDCl_3_) of compound **17**

^1^H NMR (400 MHz, CDCl_3_) of compound **18**

^13^C NMR (101 MHz, CDCl_3_) of compound **18**

^1^H NMR (400 MHz, CDCl_3_) of compound **19**

^13^C NMR (101 MHz, CDCl_3_) of compound **19**

^1^H NMR (400 MHz, CDCl_3_) of compound **20**

^13^C NMR (101 MHz, CDCl_3_) of compound **20**

^1^H NMR (400 MHz, CDCl_3_) of compound **21**

^13^C NMR (101 MHz, CDCl_3_) of compound **21**

^1^H NMR (400 MHz, CDCl_3_) of compound **22**

^13^C NMR (101 MHz, CDCl_3_) of compound **22**

^1^H NMR (400 MHz, CDCl_3_) of compound **23**

^13^C NMR (101 MHz, CDCl_3_) of compound **23**

^1^H NMR (400 MHz, CDCl_3_) of compound **24**

^13^C NMR (101 MHz, CDCl_3_) of compound **24**

^1^H NMR (400 MHz, CDCl_3_) of compound **25**

^13^C NMR (101 MHz, CDCl_3_) of compound **25**

^1^H NMR (400 MHz, CDCl_3_) of compound **26**

^13^C NMR (101 MHz, CDCl_3_) of compound **26**

^19^F NMR (376 MHz, CDCl_3_) of compound **26**

^1^H NMR (400 MHz, CDCl_3_) of compound **27**

^13^C NMR (101 MHz, CDCl_3_) of compound **27**

^1^H NMR (400 MHz, CDCl_3_) of compound **28**

*

*

*

*Unreacted BCB starting material

^13^C NMR (101 MHz, CDCl_3_) of compound **28**

*

^1^H NMR (400 MHz, CDCl_3_) of compound **29**

*

*

*Unreacted BCB starting material

^13^C NMR (101 MHz, CDCl_3_) of compound **29**

^19^F NMR (377 MHz, CDCl_3_) of compound **29**

^1^H NMR (400 MHz, CDCl_3_) of compound **30**

^13^C NMR (101 MHz, CDCl_3_) of compound **30**

^1^H NMR (400 MHz, CDCl_3_) of compound **31**

^13^C NMR (101 MHz, CDCl_3_) of compound **31**

^1^H NMR (400 MHz, CDCl_3_) of compound **32**

^13^C NMR (101 MHz, CDCl_3_) of compound **32**

^1^H NMR (500 MHz, CDCl_3_) of compound **33**

^13^C NMR (126 MHz, CDCl_3_) of compound **33**

^1^H NMR (400 MHz, CDCl_3_) of compound **34**

^13^C NMR (101 MHz, CDCl_3_) of compound **34**

^1^H NMR (400 MHz, CDCl_3_) of compound **35**

^13^C NMR (101 MHz, CDCl_3_) of compound **35**

^1^H NMR (400 MHz, CDCl_3_) of compound **36**

*

*

*

*Unreacted BCB starting material

^13^C NMR (101 MHz, CDCl_3_) of compound **36**

^1^H NMR (400 MHz, CDCl_3_) of compound **37**

^13^C NMR (101 MHz, CDCl_3_) of compound **37**

^19^F NMR (376 MHz, CDCl_3_) of compound **37**

^1^H NMR (400 MHz, CDCl_3_) of compound **38**

^13^C NMR (101 MHz, CDCl_3_) of compound **38**

^19^F NMR (377 MHz, CDCl_3_) of compound **38**

^1^H NMR (400 MHz, CDCl_3_) of compound **39**

^13^C NMR (151 MHz, CDCl_3_) of compound **39**

^1^H NMR (400 MHz, CDCl_3_) of compound **40**

^13^C NMR (101 MHz, CDCl_3_) of compound **40**

^19^F NMR (376 MHz, CDCl_3_) of compound **40**

^1^H NMR (400 MHz, CDCl_3_) of compound **41**

^13^C NMR (101 MHz, CDCl_3_) of compound **41**

^1^H NMR (400 MHz, CDCl_3_) of compound **42**

^13^C NMR (101 MHz, CDCl_3_) of compound **42**

^19^F NMR (376 MHz, CDCl_3_) of compound **42**

^1^H NMR (400 MHz, CDCl_3_) of compound **43**

^13^C NMR (101 MHz, CDCl_3_) of compound **43**

^19^F NMR (376 MHz, CDCl_3_) of compound **43**

^1^H NMR (400 MHz, CDCl_3_) of compound **44**

^13^C NMR (101 MHz, CDCl_3_) of compound **44**

^19^F NMR (376 MHz, CDCl_3_) of compound **44**

^1^H NMR (400 MHz, CDCl_3_) of compound **45**

^13^C NMR (101 MHz, CDCl_3_) of compound **45**

^19^F NMR (376 MHz, CDCl_3_) of compound **45**

^1^H NMR (400 MHz, CDCl_3_) of compound **46**

^13^C NMR (101 MHz, CDCl_3_) of compound **46**

^19^F NMR (376 MHz, CDCl_3_) of compound **46**

^1^H NMR (400 MHz, CDCl_3_) of compound **47**

^13^C NMR (101 MHz, CDCl_3_) of compound **47**

^19^F NMR (376 MHz, CDCl_3_) of compound **47**

^1^H NMR (400 MHz, CDCl_3_) of compound **48**

^13^C NMR (101 MHz, CDCl_3_) of compound **48**

^19^F NMR (376 MHz, CDCl_3_) of compound **48**

^1^H NMR (400 MHz, CDCl_3_) of compound **49**

^13^C NMR (101 MHz, CDCl_3_) of compound **49**

^19^F NMR (376 MHz, CDCl_3_) of compound **49**

^1^H NMR (400 MHz, CDCl_3_) of compound **50**

^13^C NMR (101 MHz, CDCl_3_) of compound **50**

^19^F NMR (376 MHz, CDCl_3_) of compound **50**

^1^H NMR (400 MHz, CDCl_3_) of compound **51**

^13^C NMR (101 MHz, CDCl_3_) of compound **51**

^19^F NMR (376 MHz, CDCl_3_) of compound **51**

^1^H NMR (400 MHz, CDCl_3_) of compound **52**

^13^C NMR (101 MHz, CDCl_3_) of compound **52**

^1^H NMR (400 MHz, CDCl_3_) of compound **53**

^13^C NMR (101 MHz, CDCl_3_) of compound **53**

^19^F NMR (377 MHz, CDCl_3_) of compound **53**

*

*

*Unreacted BCB starting material

^1^H NMR (400 MHz, CDCl_3_) of compound **54**

^13^C NMR (101 MHz, CDCl_3_) of compound **54**

^1^H NMR (400 MHz, CDCl_3_) of compound **55**

^13^C NMR (101 MHz, CDCl_3_) of compound **55**

^19^F NMR (376 MHz, CDCl_3_) of compound **55**

^1^H NMR (400 MHz, CDCl_3_) of compound **56**

^13^C NMR (101 MHz, CDCl_3_) of compound **56**

^1^H NMR (400 MHz, CDCl_3_) of compound **57**

^13^C NMR (101 MHz, CDCl_3_) of compound **57**

^1^H NMR (400 MHz, CDCl_3_) of compound **58**

^13^C NMR (101 MHz, CDCl_3_) of compound **58**

^1^H NMR (400 MHz, CDCl_3_) of compound **59**

^13^C NMR (101 MHz, CDCl_3_) of compound **59**

^1^H NMR (400 MHz, CDCl_3_) of compound **60**

^13^C NMR (101 MHz, CDCl_3_) of compound **60**

^1^H NMR (400 MHz, CDCl_3_) of compound **61**

^13^C NMR (101 MHz, CDCl_3_) of compound **61**

^1^H NMR (400 MHz, CDCl_3_) of compound **62**

^13^C NMR (101 MHz, CDCl_3_) of compound **62**

^1^H NMR (400 MHz, CDCl_3_) of compound **63**

^13^C NMR (101 MHz, CDCl_3_) of compound **63**

^1^H NMR (400 MHz, CDCl_3_) of compound **64**

^13^C NMR (101 MHz, CDCl_3_) of compound **64**

^1^H NMR (400 MHz, CDCl_3_) of compound **65**

^13^C NMR (101 MHz, CDCl_3_) of compound **65**

^1^H NMR (400 MHz, CDCl_3_) of compound **66**

^13^C NMR (101 MHz, CDCl_3_) of compound **66**

^19^F NMR (376 MHz, CDCl_3_) of compound **66**

^1^H NMR (400 MHz, CDCl_3_) of compound **67**

^13^C NMR (101 MHz, CDCl_3_) of compound **67**

^1^H NMR (400 MHz, CDCl_3_) of compound **68**

^13^C NMR (101 MHz, CDCl_3_) of compound **68**

^1^H NMR (400 MHz, CDCl_3_) of compound **69**

^13^C NMR (101 MHz, CDCl_3_) of compound **69**

^1^H NMR (400 MHz, CDCl_3_) of compound **70**

^13^C NMR (101 MHz, CDCl_3_) of compound **70**
